# Supplementary material for: WaSH CQI: Applying continuous quality improvement methods to water service delivery in four districts of rural northern Ghana
Source: PLoS One. 2020 Jul 15;15(7):e0233679. doi: 10.1371/journal.pone.0233679 (PMC7363065; doi:10.1371/journal.pone.0233679)
Supplement: S1 File — (DOCX) [file pone.0233679.s001.docx]

WaSH CQI: Applying Continuous Quality Improvement methods to Water Service Delivery in four districts of rural northern Ghana

Authors: Michael B. Fisher^1^*; Leslie Danquah^2^; Zakariah Seidu^3^ Allison N. Fechter^4^; Bansaga Saga^5^; Jamie K. Bartram^1^; Kaida M. Liang^1^; Rohit Ramaswamy^6^*

1. The Water Institute at UNC, Department of Environmental Sciences and Engineering, University of North Carolina at Chapel Hill, Chapel Hill, NC USA

2. School of Geosciences, University of Energy and Natural Resources, Sunyani, Ghana.

3. West African Centre for Cell Biology of Infectious Pathogens, University of Ghana, Legon, Ghana.

4. The Water Project, Concord, NH USA

5. Solidarites International, Clichy, FRANCE

6. Public Health Leadership Program, Gillings School of Global Public Health, University of North Carolina, Chapel Hill, NC USA

*Correspondence: mbfisher@gmail.com (MBF); ramaswam@email.unc.edu (RR); Tel.: +1-919-966-2480

## File S1. Data Collection Plan.

Data will be collected on water, sanitation, and hygiene (WaSH) conditions in approximately 230 communities across 4 districts using a cluster-randomized sampling approach with repeated measures.

Data will be collected on:

- Water points
- Sanitation facilities
- WaSH committees
- Households
- Water quality

Data collection will be conducted using the following instruments:

- Waterpoint Survey: collect facility-level data on water points and water quality through interviews with facility administrators and direct observation
- Sanitation Facility Survey: collect facility-level data on sanitation facilities through interviews with facility administrators/owners and direct observation
- Community Survey: collect community-level data on WaSH committee functionality and sanitation practices through interviews
- Household Survey: collect household-level data on access to WaSH services, household water treatment and storage, and basic demographic information through interviews and direct observation
- Microbiological Survey: collect data on *E. coli* concentrations in source water (directly from waterpoints) and from household stored water.

**Data Collection:**

| Instrument | Data level | Participant(s) | Method used | Number |
| --- | --- | --- | --- | --- |
| Waterpoint survey | Waterpoint | Waterpoint administrator | Interview, direct observation | All waterpoints in 230 communities |
| Community Survey | Community | WaSH committee if extant, else community leader | Interview | All 230 communities |
| Household Survey | Household | Adult respondent, pref. female head of HH | Interview, direct observation | 5-6 households per community across a random subset of 40% of communities |
| Sanitation Facility Survey | San. Facility | Sanitation facility owner/administrator | Interview, direct observation | All sanitation facilities in the subset of communities where HH surveys are conducted |
| Water quality | Community & HH |  | Chemical tests on site, microbial tests done using** | All waterpoints and all households included in waterpoint and household surveys |

Waterpoint surveys will be conducted at all public water points. Community Surveys will be conducted in all communities. Household surveys will be conducted in a subset of 40% of communities (approximately 92 communities) randomly selected from the total sample of 230 communities, with 6 households randomly selected in each of the 92 communities (500-600 total HHs).

**Variables:**

Outcome Variables:

- Water point functionality – measured by direct observation on day of visit
- WaSH committee functionality
- Stored water quality
- Use of improved sanitation

The study will also collect the following potential determinants and/or stratifying variables:

Demographic

- Geography (District)
- Household Education level
- Household Wealth
- Community population

Facility Characteristics

- Type of water point
- Age of water point
- Waterpoint hydrogeological characteristics (where available)
- Population per water point

Management

- Tariff collection
- Water points per WaSH committee

Seasonal

- Season in which data collection occurred

Other

- Reported responsiveness of external support personnel
- And others.

**Selection:**

The communities in which household surveys will be conducted will be randomly selected from the sample of 230 communities. The households to be surveyed in each community will be randomly selected as follows:

- All households in a community will be enumerated either directly or remotely (using satellite images via Google Earth), and 6 households to be visited (as well as 6 alternates) will be randomly selected from the list of all households in the community. Since households in this region are typically clustered in extended family compounds, this process will result in the identification of 6 compounds and 6 alternates. Within each compound, one household will be selected at random to be surveyed if >1 household in the compound.

In all cases, the same households will be followed for the course of the study.
