# Supplementary material for: WaSH CQI: Applying continuous quality improvement methods to water service delivery in four districts of rural northern Ghana
Source: PLoS One. 2020 Jul 15;15(7):e0233679. doi: 10.1371/journal.pone.0233679 (PMC7363065; doi:10.1371/journal.pone.0233679)
Supplement: S2 File — (DOCX) [file pone.0233679.s002.docx]

WaSH CQI: Applying Continuous Quality Improvement methods to Water Service Delivery in four districts of rural northern Ghana

Authors: Michael B. Fisher^1^*; Leslie Danquah^2^; Zakariah Seidu^3^ Allison N. Fechter^4^; Bansaga Saga^5^; Jamie K. Bartram^1^; Kaida M. Liang^1^; Rohit Ramaswamy^6^*

1. The Water Institute at UNC, Department of Environmental Sciences and Engineering, University of North Carolina at Chapel Hill, Chapel Hill, NC USA

2. School of Geosciences, University of Energy and Natural Resources, Sunyani, Ghana.

3. West African Centre for Cell Biology of Infectious Pathogens, University of Ghana, Legon, Ghana.

4. The Water Project, Concord, NH USA

5. Solidarites International, Clichy, FRANCE

6. Public Health Leadership Program, Gillings School of Global Public Health, University of North Carolina, Chapel Hill, NC USA

*Correspondence: mbfisher@gmail.com (MBF); ramaswam@email.unc.edu (RR); Tel.: +1-919-966-2480

## File S2. Detailed Description of CQI Process Steps.

DEFINE

In the define step, the CQI team formed, received training in basic CQI methods [[1](#_ENREF_1)], and developed a problem statement and team charter focused on improving stored household water quality and improving water source functionality (File S3). The team also conducted process mapping of current water source implementation and maintenance practices (Figure S6.1).

MEASURE

In the measure step, the CQI team reviewed their service delivery process and developed a targeted data collection plan to monitor its performance and potential determinants thereof (File S1). Survey tools (File S4) were developed and baseline data were collected in all four districts.

ANALYZE

Data were analyzed at UNC using STATA (Statacorp, College Station, TX). Variables measured are described in the data collection plan (File S1). Descriptive statistics were calculated, and univariable and multivariable regressions were performed on identified outcomes and potential determinants (File S6), and used to assess potential root causes identified in MEASURE.

IDENTIFY

The full CQI team leveraged members’ local and organization knowledge to brainstorm improvement options to address the root causes identified in ANALYZE, and used structured decision-making tools (e.g. Pugh Matrix) to identify suitable options for implementation (Table S6.1-6.16, S7.1, Figure S6.1-6.2, S7.1). These options comprised a preliminary improvement package (Table 2, File S8).

IMPLEMENT

A plan was created to iteratively test and refine the improvement package in the study setting (Table 1) before implementing across intervention communities.

Prototype safe water storage containers (SWSCs) were designed and constructed locally to prevent secondary contamination of HSW (File S8). An initial WSMT refresher training curriculum was developed to improve WSMTs’ capacities to operate and maintain water sources. These improvements, along with the delivery of replacement tools for water source repair, were tested in three randomly-selected intervention communities (Improvement package V 1.0). Briefly, WaSH committee training and any required water source maintenance tools (Figure 1) that WSMTs did not possess (based on a checklist developed by WVG) were delivered at the community level; the six randomly selected households surveyed at baseline received SWSCs and instruction on their use. Household- and community-level uptake surveys (File S4) were subsequently conducted in the three test communities, as well as three randomly selected control communities, to assess uptake and performance of the improvement package. Enumerators also collected observations and user feedback. The improvement package was refined based on these findings (V 1.1), and implemented in another three test communities. Uptake surveys and refinement were repeated as described above (Table 4). This iterative testing process continued until a final improvement package was identified (File S8).

The final improvement package was implemented in remaining intervention communities. This implementation included clustered WSMT trainings, as well as delivery of refined SWSCs (Figure 2) to the selected households in each intervention community (along with instructions on use [File S8]). Implementation was also scheduled to include delivery of replacement tools to WSMTs in each intervention community (File S8, Figure 1). However, many of these tools were unavailable in Ghana, and delays in international procurement led to their delivery after midline data collection.

A second round of post-implementation monitoring was then conducted (midline round, Table 6), with the same survey tools, communities, and households as at baseline, to assess impacts of the improvement package. Some loss to follow-up occurred in each subsequent round of monitoring, as some households visited at baseline were unavailable during subsequent monitoring rounds (Table 6). Delivery of tools was then conducted, and a final round of endline monitoring was carried out (Table 6).

SUSTAIN

Based on the results of the monitoring activities in IMPLEMENT, successful improvement package elements were standardized. Standardization included the development of standard operating procedures (SOPs) for the incorporation of these improvements into ongoing WaSH programs. Scale-up plans for implementing the improvement package at a regional level were developed based on the IHI Framework for Going to Full Scale [[2](#_ENREF_2)].

References

1. Michael B. Fisher, et al., *Continuous Quality Improvement in WaSH Manual and Implementation Guide*. 2017, University of North Carolina at Chapel Hill: Chapel Hill, NC, USA.

2. Barker, P.M., A. Reid, and M.W. Schall, *A framework for scaling up health interventions: lessons from large-scale improvement initiatives in Africa.* Implementation Science, 2015. **11**(1): p. 12.
