# Supplementary material for: WaSH CQI: Applying continuous quality improvement methods to water service delivery in four districts of rural northern Ghana
Source: PLoS One. 2020 Jul 15;15(7):e0233679. doi: 10.1371/journal.pone.0233679 (PMC7363065; doi:10.1371/journal.pone.0233679)
Supplement: S3 File — (DOCX) [file pone.0233679.s003.docx]

WaSH CQI: Applying Continuous Quality Improvement methods to Water Service Delivery in four districts of rural northern Ghana

Authors: Michael B. Fisher^1^*; Leslie Danquah^2^; Zakariah Seidu^3^ Allison N. Fechter^4^; Bansaga Saga^5^; Jamie K. Bartram^1^; Kaida M. Liang^1^; Rohit Ramaswamy^6^*

1. The Water Institute at UNC, Department of Environmental Sciences and Engineering, University of North Carolina at Chapel Hill, Chapel Hill, NC USA

2. School of Geosciences, University of Energy and Natural Resources, Sunyani, Ghana.

3. West African Centre for Cell Biology of Infectious Pathogens, University of Ghana, Legon, Ghana.

4. The Water Project, Concord, NH USA

5. Solidarites International, Clichy, FRANCE

6. Public Health Leadership Program, Gillings School of Global Public Health, University of North Carolina, Chapel Hill, NC USA

*Correspondence: mbfisher@gmail.com (MBF); ramaswam@email.unc.edu (RR); Tel.: +1-919-966-2480

## File S3: CQI Project Charter

# World Vision Ghana WaSH CQI Pilot Charter (Names Redacted)

| ***Product/Service*** | WaSH | | ***Process Leader*** | | | | Bansaga Saga | |
| --- | --- | --- | --- | --- | --- | --- | --- | --- |
| ***Organization*** | WV-G | | ***Phone Number for Team Leader*** | | | |  | |
| ***Regional Sponsor*** | Samuel Diarra | | ***Email for Team Leader*** | | | |  | |
|  |  |  | | | | | | |
| **Element** | **Description** | **Specifications** | | | | | | |
| **1. Process** | Name of process to be improved. | 1. Project Design 2. Software Implementation 3. Maintain Services 4. Service Utilization | | | | | | |
| **2. Project Description** | What practical problem will be solved? What is project’s purpose? | 1. Improve water source functionality 2. Improve water safety | | | | | | |
| **3. Objective** | What metrics will be improved, what is the current performance for those metrics and how much improvement is targeted? Provide specifics on how metrics are computed. | **Metrics** | | **Current** | **Goal** | **Improvement** | | **units** |
|  |  | WSMT Functionality | | X | X + 15% | 15% | | % |
|  |  | Source Functionality | | ~75% | 85% | 10% | | % |
|  |  | Downtime | | X | 0.7 X | 30% | | % |
|  |  | *E. coli* Nondetect | | X | X + 15% | 15% | | % |
|  |  | Safe Water Storage | | X | X + 15% | 15% | | % |
| **4. Process Scope** | Which process steps will be considered in this project? What is the first step and what is the last step? | 1. Project Design 2. Software Implementation 3. Maintain Services 4. Service Utilization | | | | | | |
| **5. Business Case** | Justification for this project. Why is it important? Why is it critical to business success? | To realize World Vision’s fundamental vision of “life in all its fullness,” every child must enjoy healthy development and the opportunity to pursue an adequate education. Sustainable year-round access to enough safe drinking water, adequate sanitation, and hygiene is critical to these outcomes. We have an opportunity to enhance this access by efficiently improving the functionality of the customer’s water points and the quality of their drinking water, as well as increasing the uptake of hygiene and sanitation practices. In addition, we can support these efforts by increasing WaSH committee functionality. | | | | | | |
| **6. Benefit to Internal and External Customers** | How will internal or external customers benefit from this project? How does improvement in the metrics that you have selected help them improve their performance? | Beneficiaries will enjoy improved health and livelihoods (life in all its fullness) as a result of: 1) improved access to sustainable safe water. Strengthening WSMTs will increase the sustainability of these benefits. | | | | | | |
| **7. Team members** | Names and roles of team members. | Samuel Diarra: Regional Sponsor  Attah Arhin: National Sponsor  **Bansaga Saga: Process Leader**  **John Karbo: Data Management Expert**  **Jarvis Ayamsegna: Water Quality Expert**  **Kwame Addo: Operations Expert (hardware)**  **Dominic Dapaah: Operations Expert (software)**  **Rohit Ramaswamy: Facilitator**  **Mike Fisher: Facilitator**  Charles Nachinab: Training and Validation Partner | | | | | | |
