# Supplementary material for: WaSH CQI: Applying continuous quality improvement methods to water service delivery in four districts of rural northern Ghana
Source: PLoS One. 2020 Jul 15;15(7):e0233679. doi: 10.1371/journal.pone.0233679 (PMC7363065; doi:10.1371/journal.pone.0233679)
Supplement: S4 File — (DOCX) [file pone.0233679.s004.docx]

WaSH CQI: Applying Continuous Quality Improvement methods to Water Service Delivery in four districts of rural northern Ghana

Authors: Michael B. Fisher^1^*; Leslie Danquah^2^; Zakariah Seidu^3^ Allison N. Fechter^4^; Bansaga Saga^5^; Jamie K. Bartram^1^; Kaida M. Liang^1^; Rohit Ramaswamy^6^*

1. The Water Institute at UNC, Department of Environmental Sciences and Engineering, University of North Carolina at Chapel Hill, Chapel Hill, NC USA

2. School of Geosciences, University of Energy and Natural Resources, Sunyani, Ghana.

3. West African Centre for Cell Biology of Infectious Pathogens, University of Ghana, Legon, Ghana.

4. The Water Project, Concord, NH USA

5. Solidarites International, Clichy, FRANCE

6. Public Health Leadership Program, Gillings School of Global Public Health, University of North Carolina, Chapel Hill, NC USA

*Correspondence: mbfisher@gmail.com (MBF); ramaswam@email.unc.edu (RR); Tel.: +1-919-966-2480

## File S4. Survey Tools.

Note: These CQI survey tools were adapted from monitoring and evaluation tools deployed in multiple settings. This is why multiple country options are listed in each survey. Furthermore, water sources implemented by a variety of different entities and organizations were encountered. This is why multiple implementer options are listed in each survey.

Community Survey

Question Response

Metadata

1. Date _________________________

2. Time: Enter hour _________________________

3. Time: Enter minutes _________________________

4. GPS Coordinates _________________________

5. Country

Burkina Faso______

Ethiopia______

Ghana______

India______

Mali______

Mexico______

Niger______

6. Region _________________________

7. District ID _________________________

8. Organization

CARE______

CRS______

One Drop______

UNC______

UNICEF______

WaterAid______

World Vision______

WSA______

9. Your name _________________________

10. Name of community _________________________

11. Community ID _________________________

Community Characteristics

12. How many people live in this community? _________________________

13. How many water points does this community have? _________________________

14. How many of these water points are currently functioning? _________________________

15. How many boreholes does this community have? _________________________

16. How many of these boreholes are currently functioning? _________________________

15. How many public taps or standpipes does this community have? _________________________

16. How many of these public taps or standpipes are currently functioning? _________________________

Sanitation and Hygiene Practices

17. Has this community been certified as ODF?

Yes______

No______

Not applicable______

Don't know______

Decline to state______

18. [Photo] Take a photo of the ODF sign or other ODF documentation _________________________

19. [Direct Observation] Year community certified ODF [If observation not possible ask] When year this community certified as ODF? _________________________

20. How many toilet facilities/latrines are there in this community? _________________________

21. How are children's feces disposed of in this community?

Child used toilet/latrine______

Put/rinsed into toilet or latrine______

Put/rinsed into drain or ditch______

Thrown into garbage bin or pile______

Buried______

Left in the open______

Not applicable______

Don't know______

Decline to state.______

22. [Direct Observation] Are visible excreta present in the community?

Yes______

No______

Not applicable______

Don't know______

Decline to state______

23. Is there anyone in this community who is responsible for promoting hygiene?

Yes______

No______

Not applicable______

Don't know______

Decline to state______

Only answer if you responded Yes to Q23

24. Have any hygiene promotion activities been conducted in this community?

Yes______

No______

Not applicable______

Don't know______

Decline to state______

Only answer if you responded Yes to Q24

25. How recently have hygiene promotion activities been conducted?

Within the last two weeks______

Within the last month______

Within the last year______

Over a year ago______

Not applicable______

Don't know______

Decline to state______

WaSH Committee Module

26. Is there a WaSH/Watsan committee in this community that manages drinking water facilities?

Yes______

No______

Not applicable______

Don't know______

Decline to state______

27. What is the name of the WaSH committee chairperson or most senior WaSH committee member? _________________________

28. List the name of one WaSH committee member with a mobile phone who can act as a contact _________________________

29. Please provide the full mobile number of this committee member _________________________

30. List the name of another WaSH committee member with a mobile phone who can act as a contact _________________________

31. Please provide the full mobile number of this committee member _________________________

Only answer if you responded Yes to Q26

32. Does the WaSH committee have a system manager?

Yes______

No______

Not Applicable______

Don't Know______

Decline to state______

Only answer if you responded Yes to Q26

33. Does the WaSH committee have a system operator?

Yes______

No______

Not Applicable______

Don't Know______

Decline to state______

Only answer if you responded Yes to Q33

34. Has the system operator received technical training?

Yes______

No______

Not Applicable______

Don't Know______

Decline to State______

Only answer if you responded Yes to Q26

35. Does the WaSH committee have an administrative or financial clerk?

Yes______

No______

Not Applicable______

Don't Know______

Decline to state______

Only answer if you responded Yes to Q26

36. Does the WaSH committee have a revenue collector?

Yes______

No______

Not Applicable______

Don't Know______

Decline to state______

37. Does each water point have a vendor?

Yes______

No______

Not Applicable______

Don't Know______

Decline to state______

Only answer if you responded Yes to Q26

38. Does the WaSH/Watsan committee meet regularly and manage the facilities?

Yes______

No______

Not applicable______

Don't know______

Decline to state______

39. When was the last time that the WaSH/Watsan committee met to discuss the WaSH facilities in this community? _________________________

Only answer if you responded Yes to Q26

40. Days, weeks, months or years?

Day(s)______

Week(s)______

Month(s)______

Year(s)______

Only answer if you responded Yes to Q26

41. Are meeting records available? (check)

Yes (records observed)______

Yes (records not observed)______

No (records not observed)______

Not Applicable______

Don't Know______

Decline to State______

42. [Direct Observation] Take a photo of most recent meeting records, if possible _________________________

Only answer if you responded Yes to Q26

43. Are maintenance records available? (check)

Yes (records observed)______

Yes (records not observed)______

No (records not observed)______

Not Applicable______

Don't Know______

Decline to State______

44. [Direct Observation] Take a photo of most recent maintenance records, if possible _________________________

Only answer if you responded Yes to Q26

45. Does the WaSH committee prepare maintenance schedules?

Yes______

No______

Not Applicable______

Don't Know______

Decline to State______

Only answer if you responded Yes to Q45

46. Does the WaSH committee undertake routine maintenance according to the maintenance schedule?

Yes______

No______

Not Applicable______

Don't Know______

Decline to State______

47. For how many water points is the WaSH committee responsible? _________________________

Only answer if you responded No to Q26

How are water systems in this community managed? _Insert Management Options

Maintenance

48. Is there anyone in the community who is responsible for repairing the community's water facilities when they break down or have a problem?

Yes______

No______

Not Applicable______

Don't Know______

Decline to State______

Only answer if you responded Yes to Q48

49. Who in the community is responsible for repairing the facility when it breaks or has a problem?

WaSH Committee______

Community leader______

Private maintenance person______

No one______

Not applicable______

Don't Know______

Decline to state______

Only answer if you responded WaSH Committee|Community leader|Private maintenance person|District/Local government to Q49

50. Has the person/persons who maintains the facility received training in this type of maintenance?

Yes______

No______

Not applicable______

Don't know______

Decline to state______

51. How long ago did the maintenance person/team in this community receive training in pump maintenance and repair? _________________________

Only answer if you responded Yes to Q50

52. Days, weeks, months or years?

Day(s)______

Week(s)______

Month(s)______

Year(s)______

53. How many people are there living in this community who have been trained to repair this water point? _________________________

Only answer if you responded WaSH Committee|Community leader|Private maintenance person|District/Local government to Q49

54. Is this community able to get the spare parts and materials needed to keep this water point functioning?

Yes______

No______

Not applicable______

Don't know______

Decline to state______

Only answer if you responded No to Q54

55. Why not?

No supplier______

Part was not in stock______

Lack of funds______

No one available to go get part______

Did not know which part to buy______

Not applicable______

Don't know______

Decline to state______

Only answer if you responded WaSH Committee|Community leader|Private maintenance person|District/Local government to Q49

56. Does the person/persons who maintains the facility possess all the necessary tools?

Yes______

No______

Not applicable______

Don't know______

Decline to state______

[Direct observation] Which tools are present List all necessary tools

Only answer if you responded WaSH Committee|Community leader|Private maintenance person|District/Local government to Q49

57. Did the maintenance person/team come the last time they were called?

Yes______

No______

Not applicable______

Don't know______

Decline to state______

58. The last time the facility needed repairs, how long did you have to wait between when the problem was first discovered and the time that the facility was repaired? _________________________

Only answer if you responded WaSH Committee|Community leader|Private maintenance person|District/Local government to Q49

59. Days, weeks, months or years?

Day(s)______

Week(s)______

Month(s)______

Year(s)______

60. Is there someone outside the community that you can call if a water facility is broken down or has a problem?

Yes______

No______

Not Applicable______

Don't Know______

Decline to State______

Only answer if you responded Yes to Q60

61. Who can the community call if they are unable to repair a water facility?

Area mechanic______

Private maintenance person______

District/Local government______

NGO or development organization______

No one______

Not applicable______

Don't Know______

Decline to state______

Only answer if you responded Yes to Q60

62. Do you have a phone number for this outside support person or team?

Yes______

No______

Not Applicable______

Don't Know______

Decline to State______

63. Could you please provide this phone number? _________________________

Only answer if you responded Yes to Q60

64. Have you ever called this outside support person/team to come repair a water facility?

Yes______

No______

Not Applicable______

Don't Know______

Decline to State______

65. When was the last time the community called this outside support person/team? _________________________

Only answer if you responded Yes to Q64

66. Days, weeks, months or years?

Day(s)______

Week(s)______

Month(s)______

Year(s)______

Only answer if you responded Yes to Q64

67. Did the support person/team come the last time they were called?

Yes______

No______

Not applicable______

Don't know______

Decline to state______

68. How long did it take them to come the last time they were called? _________________________

Only answer if you responded Yes to Q67

69. Days, weeks, months or years?

Day(s)______

Week(s)______

Month(s)______

Year(s)______

Financial

70. Do people pay to fetch water in this community?

Yes______

No______

Not applicable______

Don't know______

Decline to state______

Only answer if you responded Yes to Q70

71. How often do people pay for water in this community? Does each person pay as they fetch, or do people pay at certain times every month or year?

Every time they fetch______

daily______

weekly______

monthly______

yearly______

when the system breaks______

no fixed schedule (when they have money)______

Not applicable______

Don't Know______

Decline to state______

72. How much do people pay to fill a 20-L container once? _________________________

73. How much do people pay each day? _________________________

74. How much do people pay each week? _________________________

75. How much do people pay each month? _________________________

76. How much do people pay each year? _________________________

Only answer if you responded Every time they fetch|daily|weekly|monthly|yearly to Q71

77. Cedis or Pesewas?

Cedis______

Pesewas______

78. What percentage of people pay something for water? _________________________

79. Does the WaSH committee/community have money saved for repairing/replacing the facility when needed?

Yes______

No______

Not applicable______

Don't know______

Decline to state______

80. What is the balance that the WaSH committee/community has available for repairing/replacing the facility? _________________________

Only answer if you responded Yes to Q79

81. Cedis or Pesewas?

Cedis______

Pesewas______

82. What is the amount of funds that the WaSH committee/community collected in the last year for repairing/replacing the facility? _________________________

83. Cedis or Pesewas?

Cedis______

Pesewas______

84. What is the amount of funds that the WaSH committee/community spent in the last year on repairing/replacing the facility? _________________________

85. Cedis or Pesewas?

Cedis______

Pesewas______

Only answer if you responded Yes to Q79

86. Where are these funds kept?

Bank account______

Cash box______

With a committee member but not in a cash box______

Not applicable______

Don't Know______

Decline to State______

Only answer if you responded Yes to Q79

87. Is the WaSH committee able to access these funds when needed?

Yes______

No______

Not Applicable______

Don't Know______

Decline to State______

Only answer if you responded No to Q87

88. Why not?

Account holder died or moved away______

Key to cash box lost______

Bank refuses to allow committee to access funds______

Not Applicable______

Don't Know______

Declines to State______

Only answer if you responded Yes to Q26

89. Does the WaSH committee have a cash book or other financial records? (check)

Yes (records observed)______

Yes (records not observed)______

No (records not observed)______

Not Applicable______

Don't Know______

Decline to State______

Only answer if you responded Yes (records observed) to Q89

90. Were monthly revenue records kept last year?

Yes (records observed)______

No (records not observed)______

Not Applicable______

Don't Know______

Decline to State______

Only answer if you responded Yes (records observed) to Q89

91. Are financial records up-to-date?

Yes______

No______

Not applicable______

Don't know______

Decline to state______

91. Are all expenses and income accounted for?

Yes______

No______

Not applicable______

Don't know______

Decline to state______

92. [Photo] Take a photo of the financial records _________________________

94. In the last year, has any government person come to monitor the operation and maintenance of the waterpoints in this community?

Yes______

No______

Not Applicable______

Don't Know______

Decline to State______

93. Thank the respondent for their time. [Record your notes here] _________________________

95. End time: hour _________________________

96. End time: minute _________________________

Waterpoint Survey

Question Response

Metadata

1. Date _________________________

2. Time: Enter hour _________________________

3. Time: Enter minutes _________________________

4. GPS Coordinates _________________________

5. Country

Burkina Faso______

Ethiopia______

Ghana______

India______

Mali______

Mexico______

Niger______

6. Region _________________________

7. District ID _________________________

8. Organization

CARE______

CRS______

One Drop______

UNC______

UNICEF______

WaterAid______

World Vision______

WSA______

9. Your name _________________________

10. Community ID _________________________

11. Name of community _________________________

Facility Functionality

12. [Direct Observation] Source type

Piped water into dwelling______

Piped water to yard/plot______

Public tap/standpipe______

Mechanized borehole______

Borehole with manual pump______

Protected dug well______

Unprotected dug well______

Protected spring______

Unprotected spring______

Rainwater collection______

Pay another person to fetch/ buy filled containers from a vendor______

Bottled water- sachet water- or "pure water (sachet water)"______

Cart with small tank/drum______

Tanker-truck______

Surface water (river- dam- lake- pond- stream- canal- irrigation channels)______

13. [Direct Observation] Is water available from this source?

Yes______

No______

Not applicable______

Don't know______

Decline to state______

Only answer if you responded No to Q13

14. Has water been available from this source on any day in the past year?

Yes______

No______

Not applicable______

Don't know______

Decline to state______

15. [Direct Observation] If the water point is functional, how many pump strokes are needed before water begins to flow? _________________________

16. [Direct Observation] Use the timer to record the time required to fill the 20 liter container: minutes _________________________

17. [Direct Observation] Use the timer to record the time required to fill the 20 liter container: seconds _________________________

18. [Photo] Take a photograph of the waterpoint _________________________

Only answer if you responded No to Q13

19. Why is water not available from this source?

Water Source Inadequate______

motorized pumps inadequate______

Treatment plants inadequate______

Main storage inadequate______

Rising main inadequate______

Distribution storage inadequate______

Water points (taps) inadequate______

Utilization exceeds theoretical demand______

Pipes broken______

Tap broken______

Animals contaminated water______

Broken/missing pump handle______

Broken/missing chain______

Broken/missing pump cylinder______

Broken/missing valve______

Broken/missing rod______

Broken/missing gasket______

Broken Handpump (other above ground failure)______

Broken handpump (below-ground failure)______

Lack of electricity or fuel______

Water Vendor Did not come______

Facility locked______

Not applicable______

Don't know______

Decline to state______

Only answer if you responded No to Q13

20. Why has the system not yet been repaired?

Don't know whom to call______

Repair person did not come______

Parts not available______

Repair person unable to fix system______

Lack of funds to repair or pay fuel/electricity ______

Not applicable______

Don't know______

Decline to state______

Other_____

Only answer if you responded Piped water into dwelling| Piped water to yard/plot|Public tap/standpipe |Mechanized borehole|Borehole with manual pump |Rainwater collection|Cart with small tank/drum |Tanker-truck to Q12

21. In the past year, has the water system been broken down for more than one day (apart from seasonality problems)? If the water system is currently broken down, mark yes.

Yes______

No______

Not applicable______

Don't know______

Decline to state______

22. When did this water system last break down? _________________________

Only answer if you responded Yes to Q21

23. Days, weeks, months or years?

Day(s)______

Week(s)______

Month(s)______

Year(s)______

24. For how long was water not available from this source the last time it broke down? [If system is still broken, record time since the system broke.] _________________________

Only answer if you responded Yes to Q21

25. Days, weeks, months or years?

Day(s)______

Week(s)______

Month(s)______

Year(s)______

Only answer if you responded Borehole with manual pump to Q12

26. What was done to repair the water source the last time it broke?

Nothing______

replace chain______

replace gasket/rubber ring______

replace valve______

replace leather or rubber cup______

replace Cylinder______

replace rod______

replace bearing ________

replace metal bottom cage _________

Replace hand pump______

Retrieve fallen cylinder______

Rehabilitate borehole______

Only answer if you responded Piped water into dwelling|Piped water to yard/plot|Public tap/standpipe |Mechanized borehole to Q12

27. What was done to repair the water source the last time it broke?

Nothing______

Replace pipe______

Replace valve______

Replace pump______

Replace switch______

Repair pipe ______

28. Scan the barcode of the watersample _________________________

29. Water sample ID _________________________

Only answer if you responded Yes to Q13

30. Are you taking a duplicate sample at this water point?

Yes______

No______

31. Scan the barcode of the duplicate watersample _________________________

32. Water sample ID for duplicate _________________________

Only answer if you responded Yes to Q13

33. Are you taking a field blank sample at this water point?

Yes______

No______

34. Scan the barcode of the field blank _________________________

35. Water sample ID for field blank _________________________

Facility Characteristics - questions

36. Who is the facility administrator you are interviewing?

WaSH committee member______

Community leader______

School or institution administrator______

Private individual______

Head of household______

37. [Direct Observation] Year the water point was constructed, if visible _________________________

38. What year was this water point constructed? _________________________

39. [Direct Observation] Organization that constructed the water point, if visible

World Vision______

UNICEF______

WaterAid______

CARE______

other NGO______

Local Government______

Community______

Not applicable______

Don't know______

Decline to state______

40. Which organization constructed this water point?

World Vision______

UNICEF______

WaterAid______

CARE______

other NGO______

Local Government______

Community______

Not applicable______

Don't know______

Decline to state______

41. Does more than one family use this facility?

Yes______

No______

Not applicable______

Don't know______

Decline to state______

42. How many households use this facility? _________________________

43. How many people use this facility? _________________________

44. How many people were using this facility the last time it was working? _________________________

Only answer if you responded Yes to Q13

45. Does anyone use this water source for drinking?

Yes______

No______

Not applicable______

Don't know______

Decline to state______

Only answer if you responded No to Q13

46. Did anyone use this water source for drinking the last time water was available from this source?

Yes______

No______

Not applicable______

Don't know______

Decline to state______

47. Who in this community manages this water point?

WaSH Committee______

Community Leader______

Private person______

District/local government______

Church______

School______

Vendor______

No one______

Not applicable______

Don't Know______

Decline to State______

Only answer if you responded Piped water into dwelling|Piped water to yard/plot|Public tap/standpipe |Mechanized borehole to Q12

48. Has this community experienced any pipe breaks in the last week?

Yes______

No______

Not applicable______

Don't know______

Decline to state______

Reliability

49. Is water available from this source at all times?

Yes______

No______

Not applicable______

Don't know______

Decline to state______

50. In the past two weeks, have there been any times when water was not available for a full day or more?

Yes______

No______

Not applicable______

Don't know______

Decline to state______

51. For how many days in the last two weeks was water not available? _________________________

Only answer if you responded Yes to Q50

52. Are you able to predict which days water will be available from this source?

Yes______

No______

Not applicable______

Don't know______

Decline to state______

53. Is water available from this source at all hours of the day?

Yes______

No______

Not applicable______

Don't know______

Decline to state______

Only answer if you responded No to Q53

54. For how many hours was water not available yesterday? _________________________

Only answer if you responded No to Q53

55. Are you able to predict which hours water will be available from this source?

Yes______

No______

Not applicable______

Don't know______

Decline to state______

56. Are there months during the year that water is not available from this source?

Yes______

No______

Not applicable______

Don't know______

Decline to state______

Only answer if you responded Yes to Q56

57. During which months of the year is water not available from this source?

January______

February______

March______

April______

May______

June______

July______

August______

September______

October______

November______

December______

Not applicable______

Don't know______

Decline to state______

Do people pay to use this water point?

Yes______

No______

Not applicable______

Don't know______

Decline to state______

Facility Characteristics - observations

58. [Direct Observation] Unique water point ID _________________________

59. [Direct Observation] Implementer's source ID, if different _________________________

60. [Direct Observation] Type of water point

School WASH point______

Community WASH point______

Health Center______

Private WASH point______

Only answer if you responded Borehole with manual pump to Q12

61. [Direct Observation] What is the pump type?

India Mk II______

Afridev______

Vergnet______

Nira______

Water4______

Only answer if you responded Piped water into dwelling|Piped water to yard/plot|Public tap/standpipe|Mechanized borehole|Borehole with manual pump|Protected dug well|Unprotected dug well|Protected spring|Unprotected spring|Rainwater collection|Surface water (river, dam, lake, pond, stream, canal,) to Q12

62. [Direct Observation] Is there a latrine within 10 meters of the water point?

Yes______

No______

Not applicable______

Don't know______

Decline to state______

Only answer if you responded Piped water into dwelling|Piped water to yard/plot|Public tap/standpipe|Mechanized borehole|Borehole with manual pump|Protected dug well|Unprotected dug well|Protected spring|Unprotected spring|Surface water (river, dam, lake, pond, stream, canal,) to Q12

63. [Direct Observation] Is the nearest latrine on higher ground than the water point?

Yes______

No______

Not applicable______

Don't know______

Decline to state______

Only answer if you responded Piped water into dwelling|Piped water to yard/plot|Public tap/standpipe|Mechanized borehole|Borehole with manual pump|Protected dug well|Unprotected dug well|Protected spring|Unprotected spring|Surface water (river, dam, lake, pond, stream, canal,) to Q12

64. [Direct Observation] Is there human excreta on the ground within 10 meters of the water point?

Yes______

No______

Not applicable______

Don't know______

Decline to state______

Only answer if you responded Piped water into dwelling|Piped water to yard/plot|Public tap/standpipe|Mechanized borehole|Borehole with manual pump|Protected dug well|Unprotected dug well|Protected spring|Unprotected spring|Surface water (river, dam, lake, pond, stream, canal,) to Q12

[Direct Observation] Is there animal excreta on the ground within 10 meters of the water point?

Yes______

No______

Not applicable______

Don't know______

Decline to state______

Only answer if you responded Piped water into dwelling|Piped water to yard/plot|Public tap/standpipe|Mechanized borehole|Borehole with manual pump|Protected dug well|Unprotected dug well|Protected spring|Unprotected spring|Surface water (river, dam, lake, pond, stream, canal,) to Q12

65. [Direct Observation] Is there a sewer or gutter receiving sewage within 10 meters of the water point?

Yes______

No______

Not applicable______

Don't know______

Decline to state______

Only answer if you responded Piped water to yard/plot|Public tap/standpipe|Mechanized borehole|Borehole with manual pump|Protected dug well|Unprotected dug well|Protected spring|Unprotected spring to Q12

66. [Direct Observation] Is there any ponding of stagnant water within 2 meters of the cement floor of the water point?

Yes______

No______

Not applicable______

Don't know______

Decline to state______

Only answer if you responded Piped water to yard/plot|Public tap/standpipe|Mechanized borehole|Borehole with manual pump|Protected dug well|Unprotected dug well to Q12

67. [Direct Observation] Does the water point have a drainage channel?

Yes______

No______

Not applicable______

Don't know______

Decline to state______

Only answer if you responded Yes to Q68

68. [Direct Observation] Is the water point's drainage channel broken, cracked, in need of cleaning?

Yes______

No______

Not applicable______

Don't know______

Decline to state______

Only answer if you responded Yes to Q68

69. [Direct Observation] Is the drainage channel filled with stagnant water?

Yes______

No______

Not applicable______

Don't know______

Decline to state______

Only answer if you responded Piped water to yard/plot|Public tap/standpipe|Mechanized borehole|Borehole with manual pump|Protected dug well|Unprotected dug well|Protected spring|Unprotected spring|Surface water (river, dam, lake, pond, stream, canal,) to Q12

70. [Direct Observation] Is there fencing around the installation adequate to keep animals out?

Yes______

No______

Not applicable______

Don't know______

Decline to state______

Only answer if you responded Piped water to yard/plot|Public tap/standpipe |Mechanized borehole|Borehole with manual pump|Protected dug well to Q12

71. [Direct Observation] Does the water point have a cement floor?

Yes______

No______

Not applicable______

Don't know______

Decline to state______

Only answer if you responded Yes to Q72

72. [Direct Observation] Are there visible cracks on the cement floor around the water point?

Yes______

No______

Not applicable______

Don't know______

Decline to state______

Only answer if you responded Piped water into dwelling|Piped water to yard/plot|Public tap/standpipe |Mechanized borehole to Q12

73. [Direct Observation] Are there signs of leaks in the mains pipes feeding this system?

Yes______

No______

Not applicable______

Don't know______

Decline to state______

Only answer if you responded Piped water into dwelling|Piped water to yard/plot|Public tap/standpipe |Mechanized borehole to Q12

74. [Direct Observation] Are pipes exposed within 10 m of this waterpoint?

Yes______

No______

Not applicable______

Don't know______

Decline to state______

Only answer if you responded Mechanized borehole|Borehole with manual pump|Protected dug well to Q12

75. [Direct Observation] Are there any cracks in the walls of the water point?

Yes______

No______

Not applicable______

Don't know______

Decline to state______

Only answer if you responded Borehole with manual pump|Protected dug well to Q12

76. [Direct Observation] Do the walls of the water point's concrete pad extend below the surface of the ground at all points?

Yes______

No______

Not applicable______

Don't know______

Decline to state______

Only answer if you responded Mechanized borehole|Borehole with manual pump to Q12

77. [Direct Observation] Are the above-ground parts of the water point hardware loose at the point of attachment to base (which could permit water to enter the casing)?

Yes______

No______

Not applicable______

Don't know______

Decline to state______

Only answer if you responded Borehole with manual pump to Q12

78. [Direct Observation] Is the base of the water point adequately sealed to the concrete pad, so that water cannot enter into the borehole?

Yes______

No______

Not applicable______

Don't know______

Decline to state______

Water Safety

79. [Measure] Concentration of arsenic (ppb) _________________________

80. [Measure] Concentration of fluoride (ppm) _________________________

Water Safety - Duplicate Sample

81. [Measure] Concentration of arsenic (ppb) _________________________

82. [Measure] Concentration of fluoride (ppm) _________________________

Water Safety - Field blank

83. [Measure] Concentration of arsenic (ppb) _________________________

84. [Measure] Concentration of fluoride (ppm) _________________________

Metadata II

85. End time: hour _________________________

86. End time: minute _________________________

87. Write any of your notes here _________________________

Household Survey

Question Response

Metadata

1. Date _________________________

2. Time: hours _________________________

3. Time: minutes _________________________

4. GPS coordinates _________________________

5. Country

Burkina Faso______

Ethiopia______

Ghana______

India______

Mali______

Mexico______

Niger______

6. Region _________________________

7. District ID _________________________

8. Your name _________________________

9. Organization

CARE______

CRS______

One Drop______

UNC______

UNICEF______

WaterAid______

World Vision______

WSA______

10. Community name _________________________

11. Unique community ID _________________________

12. Household ID. If no ID flag is present, ask the respondent's permission to place an ID flag on the house so you can find it again later. _________________________

13. Full name of respondent _________________________

14. Has informed consent been obtained?

Yes______

No______

Document consent (photo)

Household characteristics - I

15. How many people live in your household? Household means the number of people living under this roof, including you. _________________________

16. [Direct Observation] Does the respondent live in a multi-household compound?

Yes______

No______

Not applicable______

Don't know______

Decline to state______

17. What is the total number of people living in this compound including yourself? _________________________

18. How many children under the age of 5 live in your household? _________________________

19. Has one or more of these children under the age of 5 had diarrhea in the past two weeks? Diarrhea means having three or more loose or liquid stools within 24 hours.

Yes______

No______

Not applicable______

Don't know______

Decline to state______

[If yes to 19] How many of the children under 5 in your household have had diarrhea in the past two weeks?

Participant

20. [Direct Observation] Is the participant male or female?

Male ______

Female______

21. How old are you? _________________________

22. Did you go fetch water yesterday?

Yes______

No______

Not applicable______

Don't know______

Decline to state______

23. How many times did you go to fetch water yesterday? _________________________

24. When you went to fetch water yesterday, which container did you use? [Estimate container size in Liters] _________________________

25. Each time you went to fetch water, how many containers like this did you carry and fill? _________________________

Person 1

26. Who else lives in this household? [Record first name only; ask respondent to begin with any other adults in the household, then list all children from oldest to youngest]. _________________________

27. Is [NAME] male or female?

Male______

Female______

28. How old is [NAME]? _________________________

29. Years, months, weeks, days?

Years______

Months______

Weeks______

Days______

30. Did [NAME] go to fetch water yesterday?

Yes______

No______

Not applicable______

Don't know______

Decline to state______

31. How many times did [NAME] go to fetch water yesterday? _________________________

32. When [NAME] went to fetch water, which container did [NAME] use? [Estimate container size in Liters] _________________________

33. Each time [NAME] goes to fetch water, how many containers like this does [NAME] carry and fill? _________________________

34. Is there another person in this house who fetched water yesterday?

Yes______

No______

Not applicable______

Don't know______

Decline to state______

Person 2

Only answer if you responded Yes to Q34

35. Who else in this household fetched water yesterday? [Record first name only; ask respondent to begin with any other adults in the household, then list all children from oldest to youngest]. _________________________

36. Is [NAME] male or female?

Male______

Female______

37. How old is [NAME]? _________________________

38. Years, months, weeks, days?

Years______

Months______

Weeks______

Days______

39. Did [NAME] go to fetch water yesterday?

Yes______

No______

Not applicable______

Don't know______

Decline to state______

40. How many times did [NAME] go to fetch water yesterday? _________________________

41. When [NAME] went to fetch water, which container did [NAME] use? [Estimate container size in Liters] _________________________

42. Each time [NAME] goes to fetch water, how many containers like this does [NAME] carry and fill? _________________________

43. Is there another person in this house who fetched water yesterday?

Yes______

No______

Not applicable______

Don't know______

Decline to state______

Person 3

Only answer if you responded Yes to Q43

44. Who else in this household fetched water yesterday? [Record first name only; ask respondent to begin with any other adults in the household, then list all children from oldest to youngest]. _________________________

45. Is [NAME] male or female?

Male______

Female______

46. How old is [NAME]? _________________________

47. Years, months, weeks, days?

Years______

Months______

Weeks______

Days______

48. Did [NAME] go to fetch water yesterday?

Yes______

No______

Not applicable______

Don't know______

Decline to state______

49. How many times did [NAME] go to fetch water yesterday? _________________________

50. When [NAME] went to fetch water, which container did [NAME] use? [Estimate container size in Liters] _________________________

51. Each time [NAME] goes to fetch water, how many containers like this does [NAME] carry and fill? _________________________

52. Is there another person in this house who fetched water yesterday?

Yes______

No______

Not applicable______

Don't know______

Decline to state______

Person 4

Only answer if you responded Yes to Q52

53. Who else in this household fetched water yesterday? [Record first name only; ask respondent to begin with any other adults in the household, then list all children from oldest to youngest]. _________________________

54. Is [NAME] male or female?

Male______

Female______

55. How old is [NAME]? _________________________

56. Years, months, weeks, days?

Years______

Months______

Weeks______

Days______

57. Did [NAME] go to fetch water yesterday?

Yes______

No______

Not applicable______

Don't know______

Decline to state______

58. How many times did [NAME] go to fetch water yesterday? _________________________

59. When [NAME] went to fetch water, which container did [NAME] use? [Estimate container size in Liters] _________________________

60. Each time [NAME] goes to fetch water, how many containers like this does [NAME] carry and fill? _________________________

61. Is there another person in this house who fetched water yesterday?

Yes______

No______

Not applicable______

Don't know______

Decline to state______

Person 5

Only answer if you responded Yes to Q61

62. Who else in this household fetched water yesterday? [Record first name only; ask respondent to begin with any other adults in the household, then list all children from oldest to youngest]. _________________________

63. Is [NAME] male or female?

Male______

Female______

64. How old is [NAME]? _________________________

65. Years, months, weeks, days?

Years______

Months______

Weeks______

Days______

66. Did [NAME] go to fetch water yesterday?

Yes______

No______

Not applicable______

Don't know______

Decline to state______

67. How many times did [NAME] go to fetch water yesterday? _________________________

68. When [NAME] went to fetch water, which container did [NAME] use? [Estimate container size in Liters] _________________________

69. Each time [NAME] goes to fetch water, how many containers like this does [NAME] carry and fill? _________________________

70. Is there another person in this house who fetched water yesterday?

Yes______

No______

Not applicable______

Don't know______

Decline to state______

Person 6

Only answer if you responded Yes to Q70

71. Who else in this household fetched water yesterday? [Record first name only; ask respondent to begin with any other adults in the household, then list all children from oldest to youngest]. _________________________

72. Is [NAME] male or female?

Male______

Female______

73. How old is [NAME]? _________________________

74. Years, months, weeks, days?

Years______

Months______

Weeks______

Days______

75. Did [NAME] go to fetch water yesterday?

Yes______

No______

Not applicable______

Don't know______

Decline to state______

76. How many times did [NAME] go to fetch water yesterday? _________________________

77. When [NAME] went to fetch water, which container did [NAME] use? [Estimate container size in Liters] _________________________

78. Each time [NAME] goes to fetch water, how many containers like this does [NAME] carry and fill? _________________________

79. Is there another person in this house who fetched water yesterday?

Yes______

No______

Not applicable______

Don't know______

Decline to state______

Person 7

Only answer if you responded Yes to Q79

80. Who else in this household fetched water yesterday? [Record first name only; ask respondent to begin with any other adults in the household, then list all children from oldest to youngest]. _________________________

81. Is [NAME] male or female?

Male______

Female______

82. How old is [NAME]? _________________________

83. Years, months, weeks, days?

Years______

Months______

Weeks______

Days______

84. Did [NAME] go to fetch water yesterday?

Yes______

No______

Not applicable______

Don't know______

Decline to state______

85. How many times did [NAME] go to fetch water yesterday? _________________________

86. When [NAME] went to fetch water, which container did [NAME] use? [Estimate container size in Liters] _________________________

87. Each time [NAME] goes to fetch water, how many containers like this does [NAME] carry and fill? _________________________

88. Is there another person in this house who fetched water yesterday?

Yes______

No______

Not applicable______

Don't know______

Decline to state______

Person 8

Only answer if you responded Yes to Q88

89. Who else in this household fetched water yesterday? [Record first name only; ask respondent to begin with any other adults in the household, then list all children from oldest to youngest]. _________________________

90. Is [NAME] male or female?

Male______

Female______

91. How old is [NAME]? _________________________

92. Years, months, weeks, days?

Years______

Months______

Weeks______

Days______

93. Did [NAME] go to fetch water yesterday?

Yes______

No______

Not applicable______

Don't know______

Decline to state______

94. How many times did [NAME] go to fetch water yesterday? _________________________

95. When [NAME] went to fetch water, which container did [NAME] use? [Estimate container size in Liters] _________________________

96. Each time [NAME] goes to fetch water, how many containers like this does [NAME] carry and fill? _________________________

97. Is there another person in this house who fetched water yesterday?

Yes______

No______

Not applicable______

Don't know______

Decline to state______

Person 9

Only answer if you responded Yes to Q97

98. Who else in this household fetched water yesterday? [Record first name only; ask respondent to begin with any other adults in the household, then list all children from oldest to youngest]. _________________________

99. Is [NAME] male or female?

Male______

Female______

100. How old is [NAME]? _________________________

101. Years, months, weeks, days?

Years______

Months______

Weeks______

Days______

102. Did [NAME] go to fetch water yesterday?

Yes______

No______

Not applicable______

Don't know______

Decline to state______

103. How many times did [NAME] go to fetch water yesterday? _________________________

104. When [NAME] went to fetch water, which container did [NAME] use? [Estimate container size in Liters] _________________________

105. Each time [NAME] goes to fetch water, how many containers like this does [NAME] carry and fill? _________________________

106. Is there another person in this house who fetched water yesterday?

Yes______

No______

Not applicable______

Don't know______

Decline to state______

Person 10

Only answer if you responded Yes to Q106

107. Who else in this household fetched water yesterday? [Record first name only; ask respondent to begin with any other adults in the household, then list all children from oldest to youngest]. _________________________

108. Is [NAME] male or female?

Male______

Female______

109. How old is [NAME]? _________________________

110. Years, months, weeks, days?

Years______

Months______

Weeks______

Days______

111. Did [NAME] go to fetch water yesterday?

Yes______

No______

Not applicable______

Don't know______

Decline to state______

112. How many times did [NAME] go to fetch water yesterday? _________________________

113. When [NAME] went to fetch water, which container did [NAME] use? [Estimate container size in Liters] _________________________

114. Each time [NAME] goes to fetch water, how many containers like this does [NAME] carry and fill? _________________________

115. Is there another person in this house who fetched water yesterday?

Yes______

No______

Not applicable______

Don't know______

Decline to state______

Person 11

Only answer if you responded Yes to Q115

116. Who else in this household fetched water yesterday? [Record first name only; ask respondent to begin with any other adults in the household, then list all children from oldest to youngest]. _________________________

117. Is [NAME] male or female?

Male______

Female______

118. How old is [NAME]? _________________________

119. Years, months, weeks, days?

Years______

Months______

Weeks______

Days______

120. Did [NAME] go to fetch water yesterday?

Yes______

No______

Not applicable______

Don't know______

Decline to state______

121. How many times did [NAME] go to fetch water yesterday? _________________________

122. When [NAME] went to fetch water, which container did [NAME] use? [Estimate container size in Liters] _________________________

123. Each time [NAME] goes to fetch water, how many containers like this does [NAME] carry and fill? _________________________

124. Is there another person in this house who fetched water yesterday?

Yes______

No______

Not applicable______

Don't know______

Decline to state______

Person 12

Only answer if you responded Yes to Q124

125. Who else in this household fetched water yesterday? [Record first name only; ask respondent to begin with any other adults in the household, then list all children from oldest to youngest]. _________________________

126. Is [NAME] male or female?

Male______

Female______

127. How old is [NAME]? _________________________

128. Years, months, weeks, days?

Years______

Months______

Weeks______

Days______

129. Did [NAME] go to fetch water yesterday?

Yes______

No______

Not applicable______

Don't know______

Decline to state______

130. How many times did [NAME] go to fetch water yesterday? _________________________

131. When [NAME] went to fetch water, which container did [NAME] use? [Estimate container size in Liters] _________________________

132. Each time [NAME] goes to fetch water, how many containers like this does [NAME] carry and fill? _________________________

133. Is there another person in this house who fetched water yesterday?

Yes______

No______

Not applicable______

Don't know______

Decline to state______

134. Are there any more people to add? How many? _________________________

Water Source

135. What is the main source of drinking-water for members of your household during the dry season?

Piped water into dwelling______

Piped water to yard/plot______

Public tap/standpipe______

Mechanized borehole______

Borehole with manual pump______

Protected dug well______

Unprotected dug well______

Protected spring______

Unprotected spring______

Rainwater collection______

Pay another person to fetch/ buy filled containers from a vendor______

Bottled water- sachet water- or "pure water (sachet water)"______

Cart with small tank/drum______

Tanker-truck______

Surface water (river- dam- lake- pond- stream- canal- irrigation channels)______

Not applicable______

Don't know______

Decline to state______

136. Are there ever times during the dry season when water is not available from [SOURCE]?

Yes______

No______

Not applicable______

Don't know______

Decline to state______

Only answer if you responded Yes to Q136

137. When your main source is not available, what other source of drinking-water for members of your household do you use in the dry season?

Piped water into dwelling______

Piped water to yard/plot______

Public tap/standpipe______

Mechanized borehole______

Borehole with manual pump______

Protected dug well______

Unprotected dug well______

Protected spring______

Unprotected spring______

Rainwater collection______

Pay another person to fetch/ buy filled containers from a vendor______

Bottled water- sachet water- or "pure water (sachet water)"______

Cart with small tank/drum______

Tanker-truck______

Surface water (river- dam- lake- pond- stream- canal- irrigation channels)______

Not applicable______

Don't know______

Decline to state______

138. [Direct Observation] Is the household's main dry season water source on-plot?

Yes______

No______

Not applicable______

Don't know______

Decline to state______

139. What is the main source of drinking-water for members of your household during the wet season?

Piped water into dwelling______

Piped water to yard/plot______

Public tap/standpipe______

Mechanized borehole______

Borehole with manual pump______

Protected dug well______

Unprotected dug well______

Protected spring______

Unprotected spring______

Rainwater collection______

Pay another person to fetch/ buy filled containers from a vendor______

Bottled water- sachet water- or "pure water (sachet water)"______

Cart with small tank/drum______

Tanker-truck______

Surface water (river- dam- lake- pond- stream- canal- irrigation channels)______

Not applicable______

Don't know______

Decline to state______

140. [Direct Observation] Is the household's main wet season water source on-plot?

Yes______

No______

Not applicable______

Don't know______

Decline to state______

141. Are there ever times during the wet season when water is not available from [SOURCE]?

Yes______

No______

Not applicable______

Don't know______

Decline to state______

Only answer if you responded Yes to Q141

142. When your main source is not available, what other source of drinking-water for members of your household do you use in the wet season?

Piped water into dwelling______

Piped water to yard/plot______

Public tap/standpipe______

Mechanized borehole______

Borehole with manual pump______

Protected dug well______

Unprotected dug well______

Protected spring______

Unprotected spring______

Rainwater collection______

Pay another person to fetch/ buy filled containers from a vendor______

Bottled water- sachet water- or "pure water (sachet water)"______

Cart with small tank/drum______

Tanker-truck______

Surface water (river- dam- lake- pond- stream- canal- irrigation channels)______

Not applicable______

Don't know______

Decline to state______

Water reliability

143. What water source did you most recently fetch water from?

Piped water into dwelling______

Piped water to yard/plot______

Public tap/standpipe______

Mechanized borehole______

Borehole with handpump______

Protected dug well______

Unprotected dug well______

Protected spring______

Unprotected spring______

Rainwater collection______

Bottled water- sachet water- or "pure water (sachet water)"______

Cart with small tank/drum______

Tanker-truck______

Surface water (river- dam- lake- pond- stream- canal- irrigation channels)______

Not applicable______

Don't know______

Decline to state______

Only answer if you responded No to Q143

144. Has there been any time in the last two weeks that you could not get any water from [source] for a full day or more?

Yes______

No______

Not applicable______

Don't know______

Decline to state______

145. For how many days in the last two weeks was water not available? _________________________

Only answer if you responded Yes to Q144

146. Are you able to predict which days water will be available from this source?

Yes______

No______

Not applicable______

Don't know______

Decline to state______

Only answer if you responded No to Q146

147. Is water available from this source at all hours of the day?

Yes______

No______

Not applicable______

Don't know______

Decline to state______

148. For how many hours was water not available yesterday? _________________________

Only answer if you responded No to Q147

149. Are you able to predict which hours water will be available from this source?

Yes______

No______

Not applicable______

Don't know______

Decline to state______

150. Are there months during the year that water is not available from this source?

Yes______

No______

Not applicable______

Don't know______

Decline to state______

Water Functionality

151. Has there been any time in the last year that you could not get any water from [source] for a full day or more (including today)?

Yes______

No______

Not applicable______

Don't know______

Decline to state______

Only answer if you responded Yes to Q151

152. For how long was water not available from your main source the last time it broke down? [If system is still broken, record time since the system broke.] _________________________

153. Days, weeks, months, years?

Day(s)______

Week(s)______

Month(s)______

Year(s)______

154. How many times has this water point broken down in the past year? _________________________

Water Quantity

155. Do you use water from your main water source for a farm or garden?

Farm______

Garden______

Both______

None______

156. Do you use your main water source for a business? [If yes, ask what type of business: mark all that apply]

no______

restaurant______

prepared food or drinks______

washing cars______

washing clothes for money______

construction______

fetching water for others for money______

Water Accessibility

157. Can you take me to the water source that you most recently fetched water from?

Yes______

No______

Sends other person to show water source______

Only answer if you responded Yes or "Sends other person to show water source" to Q151

158. Record time you start water walk: hour _________________________

159. Record time you leave for water walk: minute _________________________

160. Record GPS coordinates of water point _________________________

161. [After taking GPS coordinates, return to the house] Record time of return: hour _________________________

162. Record time of return: minute _________________________

Household water

163. Do you treat your water to make it safer for drinking?

Yes______

No______

Not applicable______

Don't know______

Decline to state______

Only answer if you responded Yes to Q163

164. What do you usually do to the water to make it safer to drink? Anything else? [Do not read choices, record all items mentioned]

boil______

Add bleach/chlorine______

Strain it through a cloth______

Use a water filter (ceramic- sand- composite- etc.)______

Solar disinfection______

Let it stand and settle______

Not applicable______

Don't know______

Decline to state______

Only answer if you responded Yes to Q163

165. In the last two weeks, how often have you treated your water:

Everyday______

Most of the days______

Half of the days______

Less than half of the days______

Not at all______

Not applicable______

Don't know______

Decline to state______

166. Scan the barcode of the household water sample _________________________

167. Water sample ID _________________________

168. Can you serve me some water the way you normally take it? [Direct Observation] Does/is the drinking-water storage container: (mark all that apply)

Have a lid that is completely covering it?______

Have a narrow opening______

Have a tap or spigot______

Beyond reach of animals (1 meter or more from the ground)?______

Clean (free of dirt- debris- garbage- faecal matter- etc.)?______

Not applicable______

Don't know______

Decline to state______

169. [Direct Observation] What was used to take water from the storage container?

Nothing (water directly poured or dispensed through a spigot or spout)______

Dipper or ladle______

Bucket______

Hands______

Cup- bowl- jar- or can______

170. What is the source of this [the water that is sampled] water?

Piped water into dwelling______

Piped water to yard/plot______

Public tap/standpipe______

Mechanized borehole______

Borehole with handpump______

Protected dug well______

Unprotected dug well______

Protected spring______

Unprotected spring______

Rainwater collection______

Bottled water- sachet water- or "pure water (sachet water)"______

Cart with small tank/drum______

Tanker-truck______

Surface water (river- dam- lake- pond- stream- canal- irrigation channels)______

Not applicable______

Don't know______

Decline to state______

XXX. [Photo] Take a photo of the water storage container

171. [Direct Observation] What is the main material of the floors inside all the rooms of the house?

Earth / Sand______

Dung______

Wood planks______

Palm / Bamboo______

Parquet or polished wood______

Vinyl or asphalt strips______

Ceramic tiles______

Cement______

Carpet______

Not applicable______

Don't know______

Decline to state______

172. [Direct Observation] What is the main material of the dwelling walls?

No walls______

Dirt/earth______

Cement______

Dung______

Cane/Palm/Tree trunks______

Bamboo with mud______

Stone with mud______

Uncovered adobe______

Plywood______

Cardboard______

Reused wood______

Stone with lime/cement______

Bricks______

Cement blocks______

Covered adobe______

Wood planks/shingles______

Not applicable______

Don't know______

Decline to state______

173. [Direct Observation] What is the main material of the dwelling roof?

No Roof______

Thatch/straw/Palm leaf______

Metal______

Wood planks______

Sod/grass and earth______

Rustic mat/woven plant material______

Palm / Bamboo______

Cardboard______

Finished Wood boards______

Calamine / Cement fibre______

Ceramic tiles______

Cement slab______

Roofing shingles______

Plastic______

Not applicable______

Don't know______

Decline to state______

Sanitation Facility characteristics

174. [Do not read answers out loud] Some people prefer to defecate in the bush or the open, some prefer to defecate in a latrine, and some prefer other places. What are the places that adult men and women in this household defecate? (mark all that apply) Probe to ask "Is there any other place?" until they finish

[Latrine]______

[Bush- field- no sanitation facilities]______

[In water body- river or lake]______

175. [Do not read answers out loud] Some people prefer to defecate in the bush or the open, some prefer to defecate in a latrine, and some prefer other places. Where are the places that boys and girls over the age of 3 in this household go to defecate (mark all that apply) Probe to ask "Is there any other place?" until they finish

[Latrine]______

[Bush- field- no sanitation facilities]______

[In water body- river or lake]______

176. [Direct Observation] According to the answers of the two previous questions, does anyone in this household defecate in a latrine?

Yes______

No______

Not applicable______

Don't know______

Decline to state______

Only answer if you responded Yes to Q176

177. Can you show me the toilet facility that you use?

Yes______

No______

Sends other person to show sanitation facility______

Only answer if you responded Yes|Sends other person to show sanitation facility to Q177

178. Sanitation facility ID _________________________

Scan barcode (Sanitation facility ID)

179. [Direct Observation] GPS coordinates of primary sanitation facility _________________________

180. [Direct Observation] Is the sanitation-facility on-plot?

Yes - in own dwelling______

Yes - in own yard/plot______

No - facility is off-plot______

Not observed______

Only answer if you responded Yes - in own dwelling|Yes - in own yard/plot to Q180

181. [Direct Observation] What type of toilet facility is it? [If “flush” or “pour-flush” and you cannot tell where the waste goes, probe] Where does it flush to?*

Flush/pour flush to piped sewer system______

Flush/pour flush to piped septic tank______

Flush/pour flush to pit latrine______

Flush/pour flush to elsewhere______

Flush/pour flush to unknown place/not sure/don't know______

Ventilated improved pit latrine (VIP)______

Pit latrine with slab______

Pit latrine without slab/open pit______

Composting toilet______

Hanging toilet/hanging latrine______

Only answer if you responded No - facility is off-plot|Not observed to Q180

182. [Direct Observation] What type of toilet facility is it? [If “flush” or “pour-flush” and you cannot tell where the waste goes, probe] Where does it flush to?

Flush/pour flush to piped sewer system______

Flush/pour flush to piped septic tank______

Flush/pour flush to pit latrine______

Flush/pour flush to elsewhere______

Flush/pour flush to unknown place/not sure/don't know______

Ventilated improved pit latrine (VIP)______

Pit latrine with slab______

Pit latrine without slab/open pit______

Composting toilet______

Hanging toilet/hanging latrine______

183. Are designated handwashing facilities present within 10 meters of the sanitation facility?

Only answer if you responded Yes|Sends other person to show sanitation facility to Q177

184. [Direct Observation] Does the facility shows signs of recent use?

Yes______

No______

Not applicable______

Don't know______

Decline to state______

Only answer if you responded Yes - in own dwelling|Yes - in own yard/plot to Q180

185. [Direct Observation] Is this facility accessible to disabled people?

Yes______

No______

186. [Photo] Take a photo of the sanitation facility _________________________

Only answer if you responded Yes - in own dwelling|Yes - in own yard/plot to Q180

187. [Direct Observation] Is the sanitation facility in working order?

In working order______

Not in working order______

Only answer if you responded Not in working order to Q187

188. [Direct Observation] Why is the sanitation facility not functioning as intended?

Facilities unreliable______

Facilities unhygienic______

Facilities poorly constructed______

Pit full______

Facilities flooded______

No water______

Locked______

Only answer if you responded Yes - in own dwelling|Yes - in own yard/plot to Q180

189. [Direct Observation] Is the inside of the sanitation facility soiled with feces?

Yes______

No______

Only answer if you responded Yes - in own dwelling|Yes - in own yard/plot to Q180

190. [Direct Observation] Is there evidence of feces on the ground within 10 meters of the sanitation facility?

Yes______

No______

Only answer if you responded Yes - in own dwelling|Yes - in own yard/plot to Q180

191. [Direct Observation] Is there an unpleasant or offensive smell within the sanitation facility which could discourage use of the facility.

Yes______

No______

Only answer if you responded Flush/pour flush to piped sewer system|Flush/pour flush to piped septic tank|Flush/pour flush to pit latrine|Flush/pour flush to elsewhere|Flush/pour flush to unknown place/not sure/don't know|Ventilated improved pit latrine (VIP)|Pit latrine with slab|Composting toilet to Q182

192. [Direct Observation] Is there evidence of cracking or damage to the toilet pedestal or squat-slab?

Yes______

No______

Only answer if you responded Flush/pour flush to piped sewer system|Flush/pour flush to piped septic tank|Flush/pour flush to pit latrine|Flush/pour flush to elsewhere|Flush/pour flush to unknown place/not sure/don't know to Q182

193. [Direct Observation] Is there any damage to the pipes or plumbing?

Yes______

No______

Only answer if you responded Flush/pour flush to pit latrine|Ventilated improved pit latrine (VIP)|Pit latrine with slab|Pit latrine without slab/open pit|Composting toilet to Q182

194. [Direct Observation] Is the pit uncovered?

Yes______

No______

Only answer if you responded No to Q194

195. [Direct Observation] Is the cover slab incompletely sealed?

Yes______

No______

Only answer if you responded Flush/pour flush to piped sewer system|Flush/pour flush to piped septic tank|Flush/pour flush to pit latrine|Ventilated improved pit latrine (VIP)|Pit latrine with slab|Pit latrine without slab/open pit|Composting toilet to Q182

196. [Direct Observation] Is there evidence that the pit or septic tank is full, overflowing or allowing wastes to leak onto the ground?

Yes______

No______

Only answer if you responded Flush/pour flush to elsewhere|Flush/pour flush to unknown place/not sure/don't know to Q182

197. [Direct Observation] Are excreta discharged directly to the ground or to an open sewer or gutter

Directly to the ground______

To an open sewer or gutter______

Other_______________

Only answer if you responded No to Q177

198. What type of toilet facility do you use? [If “flush” or “pour-flush” probe] Where does it flush to?

Flush/pour flush to piped sewer system______

Flush/pour flush to piped septic tank______

Flush/pour flush to pit latrine______

Ventilated improved pit latrine (VIP)______

pit latrine with slab______

Pit latrine without slab/open pit______

composting toilet______

bucket______

hanging toilet/hanging latrine______

No facilities or bush or field______

Not applicable______

Don't know______

Decline to state______

Only answer if you responded Yes to Q176

199. Is this facility shared with other families who are not relatives?

Yes______

No______

Not applicable______

Don't know______

Decline to state______

200. How many households (including your own) use this facility? _________________________

Only answer if you responded Yes to Q176

201. Is the sanitation facility for your household functional?

Yes______

No______

Not applicable______

Don't know______

Decline to state______

Only answer if you responded Yes|Sends other person to show sanitation facility to Q177

202. Is your household using this sanitation facility?

Yes______

No______

Not applicable______

Don't know______

Decline to state______

Sanitation reliability

Only answer if you responded Yes|Sends other person to show sanitation facility to Q177

203. Are you able to use this facility at all times?

Yes______

No______

Not applicable______

Don't know______

Decline to state______

Sanitation excreta disposal

204. Does any child younger than 3 years old live in this household?

Yes______

No______

Not applicable______

Don't know______

Decline to state______

Only answer if you responded Yes to Q204

205. [Do not read answer choices] The last time the youngest child (less than 3 years) passed feces, what was done to dispose of the feces?

[Child used toilet/latrine]______

[Put/rinsed into toilet or latrine]______

[Put/rinsed into drain or ditch]______

[Thrown into garbage bin or pile]______

[Buried]______

[Threw feces away in the open/threw in bush]______

[Left in the open]______

Not applicable______

Don't know______

Decline to state.______

206. [Direct Observation] Are excreta present in the house or yard?

Yes______

No______

Not applicable______

Don't know______

Decline to state______

207. Has any member of your household seen a person openly defecate in this community in the past two weeks?

Yes______

No______

Not applicable______

Don't know______

Decline to state______

Hygiene

208. [Direct Obervation] Are soap (or its equivalent) and water present in the household?

Present (observed)______

Present (not observed)______

Not present (observed)______

Not applicable______

Don't know______

Decline to state______

Only answer if you responded Present (observed) to Q208

209. [Direct Observation] What type of detergent or cleanser is used? Mark all that apply.

Soap______

Ash______

Mud or sand (specifically for hand hygiene)______

None______

Not applicable______

Don't know______

Decline to show______

210. Can you show me how you wash your hands? [Direct Observation] How does the respondent wash their hands? Mark all that apply

Use of water______

Use of soap______

Use of ash or other cleanser______

Rubbing motion______

Not shown______

211. [Direct Observation] Is there a fixed location for handwashing?

Yes______

No______

Not observed______

212. Photo of Hygiene facilities

213. [Do not read the options] When do you wash your hands? [Mark all that apply]

[After defecation]______

[After cleaning or changing a baby]______

[Before food preparation]______

[Before eating]______

[Before feeding a child]______

Not applicable______

Don't know______

Decline to state______

214. [Do not read the options] Are there any other times that you wash your hands? [Mark all that apply]

[After defecation]______

[After cleaning or changing a baby]______

[Before food preparation]______

[Before eating]______

[Before feeding a child]______

Not applicable______

Don't know______

Decline to state______

Household characteristics - II

215. Is there one or more able-bodied adults in the household capable of performing physical labor?

Yes______

No______

Not applicable______

Don't know______

Decline to state______

216. What is the primary occupation of the highest-earning member of your household (including yourself)?

No occupation______

farming______

raising livestock to sell______

labor or construction______

selling agricultural products______

selling other goods______

teaching______

office worker______

secretary______

government employee/civil servant______

driver______

craftsman (carpenter- metal worker- electrician- etc.)______

Banking- finance______

owns a food stall or restaurant______

selling food______

Owns a business that is not a farm or restaurant______

Pastor or other religious position ______

Not applicable______

Don't know______

Decline to state______

217. Does any member of this household have a bank account?

Yes______

No______

Not applicable______

Don't know______

Decline to state______

218. Do you or someone living in this household own this dwelling? If “no”, then ask: do you rent this dwelling from someone not living in this household?

Own______

Rent______

Neither own nor rent______

Not applicable______

Don't know______

Decline to state______

219. Does this household own any livestock, herds, other farm animals, or poultry?

Yes______

No______

Not applicable______

Don't know______

Decline to state______

Only answer if you responded Yes to Q219

220. Which animals do you own?

Cows______

goats______

Sheep______

Chickens/Guinea Fowl/poultry______

Other______

Not applicable______

Don't know______

Decline to state______

221. What other animals do you own? (list all) _________________________

222. Does any member of this household own any land that can be used for agriculture?

Yes______

No______

Not applicable______

Don't know______

Decline to state______

223. How much land does this household own? _________________________

Only answer if you responded Yes to Q223

224. Acres or hectares?

Acre(s)______

Hectare(s)______

225. Does this house have electricity?

Yes______

No______

Not applicable______

Don't know______

Decline to state______

226. Does any member of this household pay to fetch water?

Yes______

No______

Not applicable______

Don't know______

Decline to state______

Only answer if you responded Yes to Q226

227. How often do members of this household pay for water? Do you pay as you fetch, or do you pay at certain times every month or year?

Every time they fetch______

daily______

weekly______

monthly______

yearly______

when the system breaks______

no fixed schedule (when they have money)______

Not applicable______

Don't Know______

Decline to state______

228. How much do you pay each time to fill the container you showed me earlier? _________________________

229. How much do you pay each day? _________________________

230. How much do you pay each week? _________________________

231. How much do you pay each month? _________________________

232. How much do you pay each year? _________________________

Only answer if you responded Every time they fetch|daily|weekly|monthly|yearly to Q232

233. Denomination (adapt as needed to local currency)

Cedis______

Pesewas______

234. What type of fuel does your household mainly use for cooking?

Charcoal______

Wood______

Straw/Shrubs/Grass______

Electricity (electric stove)______

Liquefied Petroleum Gas (LPG)______

Natural gas______

Biogas______

Kerosene______

Coal / Lignite______

Animal dung______

Agricultural crop residue______

No food cooked in household______

Not applicable______

Don't know______

Decline to state______

235. Does any member of your household own: A working bicycle?

Yes ______

No______

Not applicable______

Don't know______

Decline to state______

236. Does any member of your household own: A working motorbike?

Yes ______

No______

Not applicable______

Don't know______

Decline to state______

237. Does your household have: A Working Car?

Yes______

No______

Not applicable______

Don't know______

Decline to state______

238. Does any member of your household own: A working Mobile Telephone?

Yes ______

No______

Not applicable______

Don't know______

Decline to state______

239. Does your household have: A working radio?

Yes ______

No______

Not applicable______

Don't know______

Decline to state______

240. Does your household have: A Working Television?

Yes ______

No______

Not applicable______

Don't know______

Decline to state______

241. Does your household have: A working refrigerator?

Yes ______

No______

Not applicable______

Don't know______

Decline to state______

242. Thank the respondent for their time [Record your notes here] _________________________

243. End time: hour _________________________

244. End time: minute _________________________

Sanitation Facility Survey

Question Response

Metadata

1. Date _________________________

2. Time: hour _________________________

3. Time: minute _________________________

4. GPS coordinates _________________________

5. Country

Burkina Faso______

Ethiopia______

Ghana______

India______

Mali______

Mexico______

Niger______

6. Region _________________________

7. District ID _________________________

8. Organization collecting the data

CARE______

CRS______

One Drop______

UNC______

UNICEF______

WaterAid______

World Vision______

WSA______

9. Your name _________________________

10. Name of community _________________________

11. Community ID _________________________

12. Sanitation Facility ID _________________________

Facility Characteristics

13. [Direct Observation] What type of sanitation facility is this (private, community, school, health center, other)?

Private sanitation facility______

Community sanitation facility______

School sanitation facility______

Health center sanitation facility______

Other institutional facility______

Other type of facility______

14. [Direct Observation] What is the institution where the facility is located? _________________________

15. [Direct Observation] What other type of sanitation facility is this? Please specify _________________________

16. [Direct Observation] What kind of toilet facility is present?

Flush/pour flush to piped sewer system______

Flush/pour flush to piped septic tank______

Flush/pour flush to pit latrine______

Flush/pour flush to elsewhere______

Flush/pour flush to unknown place/not sure/don't know______

Ventilated improved pit latrine (VIP)______

Pit latrine with slab______

Pit latrine without slab/open pit______

Composting toilet______

Hanging toilet/hanging latrine______

17. [Direct Observation] Determine whether facility shows signs of recent use

Yes______

No______

18. [Direct Observation] Is this facility accessible to disabled people?

Yes______

No______

19. Is there a handwashing station within 10 meters of the facility?

Yes______

No______

Not Applicable______

Don't Know______

Decline to State______

20. [Photo] Take a photo of the sanitation facility _________________________

Sanitary Inspection

21. [Direct Observation] Is the sanitation facility in working order?

In working order______

Not in working order______

Only answer if you responded Not in working order to Q21

22. Direct Observation] Why is the sanitation facility not functioning as intended?

Facilities unreliable______

Facilities unhygienic______

Facilities poorly constructed______

Pit full______

Facilities flooded______

No water______

Locked______

23. [Direct Observation] Is the inside of the sanitation facility soiled with feces?

Yes______

No______

24. [Direct Observation] Is there evidence of feces on the ground within 10 meters of the sanitation facility?

Yes______

No______

25. [Direct Observation] Is there an unpleasant or offensive smell within the sanitation facility which could discourage use of the facility.

Yes______

No______

26. [Direct Observation] Is there evidence of cracking or damage to the toilet pedestal or squat-slab?

Yes______

No______

27. [Direct Observation] Is there any damage to the pipes or plumbing?

Yes______

No______

28. [Direct Observation] Is the pit uncovered?

Yes______

No______

Only answer if you responded No to Q28

29. [Direct Observation] Is the cover slab incompletely sealed?

Pit uncovered______

Cover slab incompletely sealed______

30. [Direct Observation] Is there evidence that the pit or septic tank is full, overflowing or allowing wastes to leak onto the ground?

Yes______

No______

31. [Direct Observation] Are excreta discharged directly to the ground or to an open sewer or gutter

Directly to the ground______

To an open sewer or gutter______

32. [Write any of your notes here] _________________________

33. End time: hour _________________________

34. End time: minute _________________________

Community Uptake Survey

Uptake Survey Community (v. 3.0)

Question Response

Metadata

1. Date _________________________

2. Time: Enter hour _________________________

3. Time: Enter minutes _________________________

4. GPS Coordinates _________________________

5. Country

Burkina Faso______

Ethiopia______

Ghana______

India______

Mali______

Mexico______

Niger______

6. Region _________________________

7. District ID _________________________

8. Organization

CARE______

CRS______

One Drop______

UNC______

UNICEF______

WaterAid______

World Vision______

WSA______

9. Your name _________________________

10. Name of community _________________________

11. Community ID _________________________

Community Characteristics

12. How many water points does this community have? _________________________

13. How many of these water points are currently functioning? _________________________

14. How many boreholes does this community have? _________________________

15. How many of these boreholes are currently functioning? _________________________

WaSH Committee

16. Is there a WaSH/Watsan committee in this community that manages drinking water facilities?

Yes______

No______

Not applicable______

Don't know______

Decline to state______

17. How many members does the WaSH committee have? _________________________

18. Have any new members been added to the WaSH committee in this community within the last three months?

Yes______

No______

Only answer if you responded Yes to Q18

19. How many new members were added? _________________________

Only answer if you responded Yes to Q18

20. Which positions were added? (Select all that apply)

System manager______

System operator______

Administrative/financial clerk______

Revenue collector______

Vendor______

Other______

Only answer if you responded Other to Q20

21. Which positions were added (other) _________________________

22. When was the last time that the WaSH/Watsan committee met to discuss the WaSH facilities in this community? _________________________

Only answer if you responded Yes to Q16

23. Days, weeks, months or years?

Day(s)______

Week(s)______

Month(s)______

Year(s)______

24. Did the WaSH committee record any written records in the last month?

Yes______

No______

Only answer if you responded Yes to Q16

25. [Direct Observation] Take a photo of most recent meeting records, if possible _________________________

26. Has the WaSH committee received any training in the last three months?

Yes______

No______

Only answer if you responded Yes to Q26

27. How long ago was training received? _________________________

Only answer if you responded Yes to Q26

28. Days, weeks, months or years?

Days______

Weeks______

Months______

Years______

29. Has the WaSH committee used any skills or knowledge from this training in the last three months?

Yes______

No______

Only answer if you responded Yes to Q29

30. Which skills or knowledge did the committee use? _________________________

Maintenance

31. Does this community have one or more pump maintenance volunteers (PMVs)?

Yes______

No______

Only answer if you responded Yes to Q31

32. How many PMVs does this community have? _________________________

Only answer if you responded Yes to Q31

33. Were any new PMVs added in the last three months?

Yes______

No______

Only answer if you responded Yes to Q33

34. How many PMVs were added? _________________________

Only answer if you responded Yes to Q33

35. How long ago was the most recent PMV added? _________________________

Only answer if you responded Yes to Q33

36. Days, weeks, months or years?

Days______

Weeks______

Months______

Years______

37. Has the community called a PMV to repair a water system in the last three months?

Yes______

No______

38. Have the PMVs in this community received training in water system repair within the last three months?

Yes______

No______

Not applicable______

Don't Know______

Decline to state______

Only answer if you responded Yes to Q38

39. How long ago did the PMV or PMVs in this community receive training? _________________________

Only answer if you responded Yes to Q38

40. Days, weeks, months or years?

Day(s)______

Week(s)______

Month(s)______

Year(s)______

Only answer if you responded Yes to Q38

41. Has the PMV or PMVs in this community used any skills or knowledge from this training in the last three months?

Yes______

No______

Not applicable______

Don't know______

Decline to state______

Only answer if you responded Yes to Q41

42. What skills or knowledge did the PMV or PMVs use? _________________________

43. Has this community bought or received any new tools for repairing water systems in the last three months?

Yes______

No______

Not applicable______

Don't know______

Decline to state______

44. Which tools does this community have for repairing water systems

17/19-mm Combination flat spanner______

19-mm Combination spanner______

Rod lifter______

Rod Clamp______

Pipe Wrench______

Pipe lifter (Pair of two)______

Pipe clamp______

Grip pliers______

Crank spanner______

22/24-mm Ring spanner______

Axle punch______

Chain fork______

Chain Support______

Bearing Presser______

Afridev Socket Spanner______

Afridev Fishing tool______

Nira Allen Key (10mm)______

Nira F- Key______

Only answer if you responded Yes to Q43

45. Which tools were bought or received?

17/19-mm Combination flat spanner______

19-mm Combination spanner______

Rod lifter______

Rod Clamp______

Pipe Wrench______

Pipe lifter (Pair of two)______

Pipe clamp______

Grip pliers______

Crank spanner______

22/24-mm Ring spanner______

Axle punch______

Chain fork______

Chain Support______

Bearing Presser______

Afridev Socket Spanner______

Afridev Fishing tool______

Nira Allen Key (10mm)______

Nira F- Key______

Only answer if you responded Yes to Q43

46. How long ago were these tools bought or received? _________________________

Only answer if you responded Yes to Q43

47. Days, weeks, months, or years?

Days______

Weeks______

Months______

Years______

Only answer if you responded Yes to Q43

48. Does the community still have all the tools that were bought or received for water system repair within the last three months?

Yes______

No______

Not applicable______

Don't know______

Decline to state______

Only answer if you responded No to Q48

49. Why not?

Lost______

Stolen______

Sold______

Broken______

Fell down borehole______

Not applicable______

Don't know______

Decline to state______

Only answer if you responded Yes to Q43

50. Have any of these new tools been used within the last three months?

Yes______

No______

Not applicable______

Don't know______

Decline to state______

Only answer if you responded Yes to Q50

51. Which tools were used within the last three months (list all that apply)?

Rod Clamp______

Pipe Clamp______

Rod lifter______

Pipe lifter______

Pipe wrench______

Fork______

Axle punch______

19-inch combination spanner______

19/17 combination spanner______

24-inch spanner______

Grip pliers______

Chain support______

Fishing tool______

52. Are there any tools that are needed for repairing the water systems that the community still does not have?

Yes______

No______

Not applicable______

Don't know______

Decline to state______

Only answer if you responded Yes to Q52

53. Which tools are still needed for repairing the water systems that the community does not currently have?

Rod Clamp______

Pipe Clamp______

Rod lifter______

Pipe lifter______

Pipe wrench______

Fork______

Axle punch______

19-inch combination spanner______

19/17 combination spanner______

24-inch spanner______

Grip pliers______

Chain support______

Fishing tool______

54. Has there been any time in the last three months that a repair could not be completed because of the lack of tools?

Yes______

No______

Not applicable______

Don't know______

Decline to state______

Financial

55. Do people pay to fetch water in this community?

Yes______

No______

Not applicable______

Don't know______

Decline to state______

Only answer if you responded Yes to Q55

56. How often do people pay for water in this community? Does each person pay as they fetch, or do people pay at certain times every month or year?

Every time they fetch______

daily______

weekly______

monthly______

yearly______

when the system breaks______

no fixed schedule (when they have money)______

Not applicable______

Don't Know______

Decline to state______

Only answer if you responded Every time they fetch to Q56

57. How much do people pay to fill a 20-L container once? _________________________

Only answer if you responded daily to Q56

58. How much do people pay each day? _________________________

Only answer if you responded weekly to Q56

59. How much do people pay each week? _________________________

Only answer if you responded monthly to Q56

60. How much do people pay each month? _________________________

Only answer if you responded yearly to Q56

61. How much do people pay each year? _________________________

Only answer if you responded Every time they fetch|daily|weekly|monthly|yearly to Q56

62. Cedis or Pesewas?

Cedis______

Pesewas______

Only answer if you responded Every time they fetch|daily|weekly|monthly|yearly|when the system breaks|no fixed schedule (when they have money) to Q56

63. What percentage of people pay something for water? _________________________

64. Is there a revenue collector at each water point in the community?

Yes______

No______

Not applicable______

Don't know______

Decline to state______

65. Does the WaSH committee/community have money saved for repairing/replacing the facility when needed?

Yes______

No______

Not applicable______

Don't know______

Decline to state______

Only answer if you responded Yes to Q65

66. What is the balance that the WaSH committee/community has available for repairing/replacing the facility? _________________________

Only answer if you responded Yes to Q65

67. Cedis or Pesewas?

Cedis______

Pesewas______

68. Are financial records up-to-date, and are all expenses and income accounted for?

Yes______

No______

Not applicable______

Don't know______

Decline to state______

69. [Photo] Take a photo of the financial records _________________________

70. Thank the respondent for their time. [Record your notes here] _________________________

71. End time: hour _________________________

72. End time: minute _________________________

Household Uptake Survey

Uptake Survey Household (v. 2.0)

Question Response

Metadata

1. Date _________________________

2. Time: hours _________________________

3. Time: minutes _________________________

4. GPS coordinates _________________________

5. Country

Burkina Faso______

Ethiopia______

Ghana______

India______

Mali______

Mexico______

Niger______

6. Region _________________________

7. District ID _________________________

8. Your name _________________________

9. Organization

CARE______

CRS______

One Drop______

UNC______

UNICEF______

WaterAid______

World Vision______

WSA______

10. Community name _________________________

11. Unique community ID _________________________

12. Household ID. If no ID flag is present, ask the respondent's permission to place an ID flag on the house so you can find it again later. _________________________

13. Full name of respondent _________________________

14. Has informed consent been obtained?

Yes______

No______

Household characteristics - I

15. How many people live in your household? Household means the number of people living under this roof, including you. _________________________

16. [Direct Observation] Does the respondent live in a multi-household compound?

Yes______

No______

Not applicable______

Don't know______

Decline to state______

Only answer if you responded Yes to Q16

17. What is the total number of people living in this compound including yourself? _________________________

18. How many children under the age of 5 live in your household? _________________________

19. Has one or more of these children under the age of 5 had diarrhea in the past two weeks? Diarrhea means having three or more loose or liquid stools within 24 hours.

Yes______

No______

Not applicable______

Don't know______

Decline to state______

20. Are there any children in your house who are attending primary or secondary school?

Yes______

No______

Not applicable______

Don't know______

Decline to state______

Only answer if you responded Yes to Q20

21. Has one or more of these children who attend school missed one ore more days of school in the past two weeks due to illness?

Yes______

No______

Not applicable______

Don't know______

Decline to state______

22. Has any child younger than 5 who lived in this household died in the last year?

Yes______

No______

Not applicable______

Don't know______

Decline to state______

Household water

23. Do you treat your water to make it safer for drinking?

Yes______

No______

Not applicable______

Don't know______

Decline to state______

24. Has anyone brought you a container for storing drinking water in the last three months?

Yes______

No______

Only answer if you responded Yes to Q23

25. What do you usually do to the water to make it safer to drink? Anything else? [Do not read choices, record all items mentioned]

boil______

Add bleach/chlorine______

Strain it through a cloth______

Use a water filter (ceramic- sand- composite- etc.)______

Solar disinfection______

Let it stand and settle______

Not applicable______

Don't know______

Decline to state______

Only answer if you responded Yes to Q24

26. Can you show me the container?

Yes______

No______

Not applicable______

Don't know______

Decline to state______

27. [Observe] Is a safe water storage container present in the home?

Yes______

No______

Not applicable______

Don't know______

Decline to state______

Only answer if you responded Yes to Q23

28. In the last two weeks, have you treated your water:

Everyday______

Most of the days______

Half of the days______

Less than half of the days______

Not at all______

Not applicable______

Don't know______

Decline to state______

Only answer if you responded Yes to Q27

29. [Observe] Does the safe water storage container have water in it?

Yes______

No______

Not applicable______

Don't know______

Decline to state______

Only answer if you responded Yes to Q27

30. [Observe] Is the safe water storage container completely covered?

Yes______

No______

Not applicable______

Don't know______

Decline to state______

Only answer if you responded Yes to Q27

31. [Observe] Is the safe water storage contaner on a stand (not on the ground)?

Yes______

No______

Not applicable______

Don't know______

Decline to state______

32. Can you serve me some water the way you normally take it? [Direct Observation] Does/is the drinking-water storage container: (mark all that apply)

Have a lid that is completely covering it?______

Have a narrow opening______

Have a tap or spigot______

Beyond reach of animals (1 meter or more from the ground)?______

Clean (free of dirt- debris- garbage- faecal matter- etc.)?______

Not applicable______

Don't know______

Decline to state______

33. Scan the barcode of the household water sample _________________________

34. [Direct Observation] What was used to take water from the storage container?

Nothing (water directly poured or dispensed through a spigot or spout)______

Dipper or ladle______

Bucket______

Hands______

Cup- bowl- jar- or can______

35. Water sample ID _________________________

Only answer if you responded Yes to Q27

36. [Observe] Is any part of the safe water storage container cracked or broken?

Yes______

No______

Not applicable______

Don't know______

Decline to state______

37. What is the source of this [the water that is sampled] water?

Piped water into dwelling______

Piped water to yard/plot______

Public tap/standpipe______

Mechanized borehole______

Borehole with handpump______

Protected dug well______

Unprotected dug well______

Protected spring______

Unprotected spring______

Rainwater collection______

Bottled water- sachet water- or "pure water (sachet water)"______

Cart with small tank/drum______

Tanker-truck______

Surface water (river- dam- lake- pond- stream- canal- irrigation channels)______

Not applicable______

Don't know______

Decline to state______

Only answer if you responded Yes to Q36

38. [Observe] Which parts of the safe water storage container are cracked or broken?

Tap______

Container reservoir______

Stand______

Other part______

Not applicable______

Don't know______

Decline to state______

Only answer if you responded Other part to Q38

39. [Observe] What other parts of the container are cracked or broken? _________________________

40. [Direct Observation] What is the main material of the floors inside all the rooms of the house?

Earth / Sand______

Dung______

Wood planks______

Palm / Bamboo______

Parquet or polished wood______

Vinyl or asphalt strips______

Ceramic tiles______

Cement______

Carpet______

Not applicable______

Don't know______

Decline to state______

41. [Direct Observation] What is the main material of the dwelling walls?

No walls______

Dirt/earth______

Cement______

Dung______

Cane/Palm/Tree trunks______

Bamboo with mud______

Stone with mud______

Uncovered adobe______

Plywood______

Cardboard______

Reused wood______

Stone with lime/cement______

Bricks______

Cement blocks______

Covered adobe______

Wood planks/shingles______

Not applicable______

Don't know______

Decline to state______

Only answer if you responded Yes to Q27

42. [Observe] Does the safe water storage container show signs of recent use?

Yes______

No______

Not applicable______

Don't know______

Decline to state______

43. [Direct Observation] What is the main material of the dwelling roof?

No Roof______

Thatch/straw/Palm leaf______

Metal______

Wood planks______

Sod/grass and earth______

Rustic mat/woven plant material______

Palm / Bamboo______

Cardboard______

Finished Wood boards______

Calamine / Cement fibre______

Ceramic tiles______

Cement slab______

Roofing shingles______

Plastic______

Not applicable______

Don't know______

Decline to state______

Only answer if you responded Yes to Q24

44. Is your family using this container?

Yes______

No______

Not applicable______

Don't know______

Decline to state______

45. For what purpose is your family using the container?

Storing water for drinking______

Storing water for other purposes______

Storing things other than water______

For a stool______

46. Where do adults in this household mainly take water to drink when they are at home?

Safe water storage container______

Other container______

Only answer if you responded Safe water storage container to Q46

47. Do adults in this household also drink from other containers when they are at home?

Yes______

No______

Not applicable______

Don't know______

Decline to state______

48. Where do children over the age of five years old in this household mainly take water to drink when they are at home?

Safe storage container______

Other container______

Only answer if you responded Safe storage container to Q48

49. Do children in this household also drink from other containers when they are at home?

Yes______

No______

Not applicable______

Don't know______

Decline to state______

50. When children younger than five years old in this household drink water at home, where is this water taken from?

Safe storage container______

Other container______

Only answer if you responded Safe storage container to Q50

51. Is water for children under five years old in this household also taken from other containers?

Yes______

No______

Not applicable______

Don't know______

Decline to state______

Only answer if you responded Yes to Q44

52. Can you show me how you take water from the container to drink?

Fetched from tap______

Pouring______

Dipping cup or other container with hand______

Fetching with ladle______

Only answer if you responded No to Q44

53. Why is your family not using the container currently?

Tap broke______

Container reservoir broke______

Stand broke______

Other part broke______

Container lost______

Container stolen______

Container borrowed and not returned______

Container used for another purpose______

Stand used for another purpose______

Water in container had bad taste______

Water in container was too hot______

Container was too hard to fill______

Container was too hard to dispense water from______

Container was too unstable______

Other reason______

Only answer if you responded Other part broke to Q53

54. Which part broke? _________________________

Only answer if you responded Container used for another purpose to Q53

55. What other purpose was the container used for? _________________________

Only answer if you responded Stand used for another purpose to Q53

56. What other purpose was the stand used for? _________________________

Only answer if you responded Other reason to Q53

57. Other reason that family is not using safe storage container _________________________

Only answer if you responded Yes to Q44

58. How long ago was the last time you took water from the safe storage container to drink? _________________________

Only answer if you responded Yes to Q44

59. Days, weeks, months, years

Days______

Weeks______

Months______

Years______
