# Supplementary material for: WaSH CQI: Applying continuous quality improvement methods to water service delivery in four districts of rural northern Ghana
Source: PLoS One. 2020 Jul 15;15(7):e0233679. doi: 10.1371/journal.pone.0233679 (PMC7363065; doi:10.1371/journal.pone.0233679)
Supplement: S5 File — (DOCX) [file pone.0233679.s005.docx]

WaSH CQI: Applying Continuous Quality Improvement methods to Water Service Delivery in four districts of rural northern Ghana

Authors: Michael B. Fisher^1^*; Leslie Danquah^2^; Zakariah Seidu^3^ Allison N. Fechter^4^; Bansaga Saga^5^; Jamie K. Bartram^1^; Kaida M. Liang^1^; Rohit Ramaswamy^6^*

1. The Water Institute at UNC, Department of Environmental Sciences and Engineering, University of North Carolina at Chapel Hill, Chapel Hill, NC USA

2. School of Geosciences, University of Energy and Natural Resources, Sunyani, Ghana.

3. West African Centre for Cell Biology of Infectious Pathogens, University of Ghana, Legon, Ghana.

4. The Water Project, Concord, NH USA

5. Solidarites International, Clichy, FRANCE

6. Public Health Leadership Program, Gillings School of Global Public Health, University of North Carolina, Chapel Hill, NC USA

*Correspondence: mbfisher@gmail.com (MBF); ramaswam@email.unc.edu (RR); Tel.: +1-919-966-2480

## File S5. Selected Field Protocols and Operational Definitions

Note: this is an abbreviated version of the tools used for this work; protocols and operational definitions included are unchanged.

Contents

[File S5. Selected Field Protocols and Operational Definitions 1](#_Toc9883827)

[1. Selected Terms and Operational Definitions 3](#_Toc9883828)

[• Water sources 3](#_Toc9883829)

[• Sanitation facilities 4](#_Toc9883830)

[• Service Continuity 5](#_Toc9883831)

[• Operational Facility 5](#_Toc9883832)

[• On-plot Facility 5](#_Toc9883833)

[• Private facility 6](#_Toc9883834)

[• Signs of Use 6](#_Toc9883835)

[• Household 6](#_Toc9883836)

[• Safe water storage 6](#_Toc9883837)

[• Hygiene supplies 6](#_Toc9883838)

[• Handwashing facilities 7](#_Toc9883839)

[• Knowledge of critical handwashing times 7](#_Toc9883840)

[• Open-defecation free (ODF) 7](#_Toc9883841)

[1. Measurement procedures 8](#_Toc9883842)

[▪ Flow rate measurement 8](#_Toc9883843)

[▪ Distance measurement 8](#_Toc9883844)

[▪ Travel time measurement 8](#_Toc9883845)

[▪ Water point inspection 9](#_Toc9883846)

[2. Water Quality Testing Procedures 11](#_Toc9883847)

[Collection of Samples 11](#_Toc9883848)

[Materials Needed 11](#_Toc9883849)

[Procedure for Collection of Samples 11](#_Toc9883850)

[Sample storage and transport *(For off-site analysis only)* 12](#_Toc9883851)

[Chain of Custody *(For central laboratory analysis only)* 12](#_Toc9883852)

[Sample Analysis 12](#_Toc9883853)

[Microbiological Analysis 13](#_Toc9883854)

[Compartment Bag Test (Source: Aquagenx LLC) 13](#_Toc9883855)

[Physical/Chemical Analysis 16](#_Toc9883856)

[Core Parameters Measured in the Field 16](#_Toc9883857)

[A. Arsenic 16](#_Toc9883858)

[Field-based ITS Arsenic Quick™ Kit Method (Source: Industrial Test Systems, Inc.) 16](#_Toc9883859)

[B. Fluoride 19](#_Toc9883860)

[Non-core Parameters Measured in the Field 20](#_Toc9883861)

[C. pH 20](#_Toc9883862)

[pH meter method (Source: Hanna Instruments, Inc.) 20](#_Toc9883863)

[D. Conductivity/Total Dissolved Solids 21](#_Toc9883864)

[Conductivity/TDS meter method (Source: Hanna Instruments, Inc.) 21](#_Toc9883865)

[E. Turbidity 22](#_Toc9883866)

[Portable turbidimeter method (Source: Hanna Instruments, Inc.) 22](#_Toc9883867)

# Selected Terms and Operational Definitions

Terms Defined:

## Water sources

The standard WHO/UNICEF definitions for different types of water sources are used here:

Improved Water Sources

- Piped water into dwelling, also called a household connection, is defined as a water service pipe connected with in-house plumbing to one or more taps (e.g. in the kitchen and bathroom).
- Piped water to yard/plot, also called a yard connection, is defined as a piped water connection to a tap placed in the yard or plot outside the house.
- Public tap or standpipe is a public water point from which people can collect water. A standpipe is also known as a public fountain or public tap. Public standpipes can have one or more taps and are typically made of brickwork, masonry or concrete.
- Tubewell or borehole is a deep hole that has been driven, bored or drilled, with the purpose of reaching groundwater supplies. Boreholes/tubewells are constructed with casing, or pipes, which prevent the small diameter hole from caving in and protects the water source from infiltration by run-off water. Water is delivered from a tubewell or borehole through a pump, which may be powered by human, animal, wind, electric, diesel or solar means. Boreholes/tubewells are usually protected by a platform around the well, which leads spilled water away from the borehole and prevents infiltration of run-off water at the well head.
- Protected dug well is a dug well that is protected from runoff water by a well lining or casing that is raised above ground level and a platform that diverts spilled water away from the well. A protected dug well is also covered, so that bird droppings and animals cannot fall into the well.
- Protected spring. The spring is typically protected from runoff, bird droppings and animals by a "spring box", which is constructed of brick, masonry, or concrete and is built around the spring so that water flows directly out of the box into a pipe or cistern, without being exposed to outside pollution.
- Rainwater refers to rain that is collected or harvested from surfaces (by roof or ground catchment) and stored in a container, tank or cistern until used.

Unimproved Water Sources

- Unprotected spring. This is a spring that is subject to runoff, bird droppings, or the entry of animals. Unprotected springs typically do not have a "spring box".
- Unprotected dug well. This is a dug well for which one of the following conditions is true: 1) the well is not protected from runoff water; or 2) the well is not protected from bird droppings and animals. If at least one of these conditions is true, the well is unprotected.
- Cart with small tank/drum. This refers to water sold by a provider who transports water into a community. The types of transportation used include donkey carts, motorized vehicles and other means.
- Tanker-truck. The water is trucked into a community and sold from the water truck.
- Surface water is water located above ground and includes rivers, dams, lakes, ponds, streams, canals, and irrigation channels.
- Bottled water is a category that includes any type of packaged water, whether sold in a bottle, bag, sachet, or other sealed container. In some countries users may use the term “Pure water” to refer to packaged water sold in bags or sachets. Bottled water is considered to be improved only when the household uses drinking-water from an improved source for cooking and personal hygiene; where this information is not available, bottled water is classified on a case-by-case basis.

## Sanitation facilities

The standard WHO/UNICEF definitions for different types of sanitation facilities are used here:

Improved Sanitation

- Flush toilet uses a cistern or holding tank for flushing water, and a water seal (which is a U-shaped pipe below the seat or squatting pan) that prevents the passage of flies and odours. A pour flush toilet uses a water seal, but unlike a flush toilet, a pour flush toilet uses water poured by hand for flushing (no cistern is used).
- Piped sewer system is a system of sewer pipes, also called sewerage, that is designed to collect human excreta (faeces and urine) and wastewater and remove them from the household environment. Sewerage systems consist of facilities for collection, pumping, treating and disposing of human excreta and wastewater.
- Septic tank is an excreta collection device consisting of a water-tight settling tank, which is normally located underground, away from the house or toilet. The treated effluent of a septic tank usually seeps into the ground through a leaching pit. It can also be discharged into a sewerage system.
- Flush/pour flush to pit latrine refers to a system that flushes excreta to a hole in the ground or leaching pit (protected, covered).
- Ventilated improved pit latrine (VIP) is a dry pit latrine ventilated by a pipe that extends above the latrine roof. The open end of the vent pipe is covered with gauze mesh or fly-proof netting and the inside of the superstructure is kept dark.
- Pit latrine with slab is a dry pit latrine that uses a hole in the ground to collect the excreta and a squatting slab or platform that is firmly supported on all sides, easy to clean and raised above the surrounding ground level to prevent surface water from entering the pit. The platform has a squatting hole, or is fitted with a seat.
- Composting toilet is a dry toilet into which carbon-rich material (vegetable wastes, straw, grass, sawdust, ash) are added to the excreta and special conditions maintained to produce inoffensive compost. A composting latrine may or may not have a urine separation device.
- Special case. A response of "flush/pour flush to unknown place/not sure/DK where" is taken to indicate that the household sanitation facility is improved, as respondents might not know if their toilet is connected to a sewer or septic tank.

Improved sanitation options are functioning options within 200m of respondent's home

Unimproved Sanitation

- Flush/pour flush to elsewhere refers to excreta being deposited in or nearby the household environment (not into a pit, septic tank, or sewer). Excreta may be flushed to the street, yard/plot, open sewer, a ditch, a drainage way or other location.
- Pit latrine without slab uses a hole in the ground for excreta collection and does not have a squatting slab, platform or seat. An open pit is a rudimentary hole.
- Bucket refers to the use of a bucket or other container for the retention of faeces (and sometimes urine and anal cleaning material), which are periodically removed for treatment, disposal, or use as fertilizer.
- Hanging toilet or hanging latrine is a toilet built over the sea, a river, or other body of water, into which excreta drops directly.
- No facilities or bush or field includes defecation in the bush or field or ditch; excreta deposited on the ground and covered with a layer of earth (cat method); excreta wrapped and thrown into garbage; and defecation into surface water (drainage channel, beach, river, stream or sea).

## Service Continuity

- - Continuous service means that facilities are operational at any time, day or night, that users might wish to access them. A continuously operational facility to which access is restricted during certain hours (e.g. a latrine or water source that is locked at night) is not considered to provide continuous service, even if the service disruption is not due to any technical problems.
  - Service that is not continuous means that users cannot access the facility due to malfunction, closure, etc. A borehole that stops producing water for several hours each day after heavy pumping does not offer continuous service. However, a borehole that continues to produce water, but has long lines that prevent users from accessing it at certain times, would still be said to provide continuous service.

## Operational Facility

- - Water point: An operational water point is one with observable flow at the time of inspection.
  - An operational sanitation facility is one that can be used for its intended purpose by a typical individual. If a facility shows signs of regular use (see below), it is considered operational. If it does not show signs of use, but the enumerator perceives that it could be used by a typical individual, it is also considered operational. If the facility is not in use and could not be easily used by a typical individual, it is not considered to be operational.

## **On-plot Facility**

- - An on-plot facility is defined as a facility within 50 meters of the entrance to the respondents dwelling that the respondent has the right to use at all times.

## Private facility

- - A private facility is defined as a facility that is used by the respondent and the respondent’s family members (up to a total of 10 households) but not by the general public, or by more than a total of 10 households.

## Signs of Use

- - Water
    - Facility is operational at the time of visit
    - Community members observed fetching water from the facility
    - Community member and WaSH committee members (if present) report that the facility is regularly used
  - Sanitation
    - Facility is operational at the time of visit
    - Community members observed using the facility
    - Community member and WaSH committee members (if present) report that the facility is regularly used
    - Facility is not in “like-new” condition: some dirt, footprints, or other signs of wear
    - Evidence of excreta in pit, if pit is visible

## Household

- Household: A family unit consisting of one or more individuals cohabitating in a single dwelling.
- Female Head of household: An adult female household member who is recognized as the female leader of the family unit by its other members (if any). If no such household member exists, a male head of household may be interviewed.

## Safe water storage

Safe water storage is defined as the storage of water for drinking purposes in a container meeting all of the following criteria (From Mintz et al., 1995):

- Narrow mouth (<10 cm) to prevent users from introducing their hands into the opening
- Container is covered with a tightly-fitting lid at the time of the visit
- The container has a tap or spigot that is used for dispensing water
- The container is stored in an elevated location (not directly on the ground)

## Hygiene supplies

Hygiene supplies are defined as:

- Presence of water for handwashing on the premises and accessible to all users
- Soap (or its equivalent)
  - Presence of soap (or its equivalent): liquid or solid soap are available and accessible to all users upon inspection. If soap is not found, the enumerator should ask the main respondent, and note whether soap or its equivalent are produced, and whether the place in which they were stored is accessible to all family members/ users
    - Acceptable equivalents to soap include:
      - Ash stored expressly for the purpose of handwashing; Ash in a hearth or cooking space is not considered the equivalent of soap.]
      - Waterless hand sanitizer such as gelled alcohol (note, if waterless hand sanitizer is present and accessible, hygiene supplies shall be considered to be present.)

## Handwashing facilities

- A Handwashing facility/station includes any fixed location where handwashing takes place. In cultural contexts where handwashing supplies are brought to the point of use, the presence of these supplies in an accessible location shall constitute a movable “HW station.”
- For a sanitation facility to include handwashing facilities, soap (or its equivalent) and water must be within 3 m of the intended point of use. Note: if these supplies are not present within 3 m, the sanitation facility is not considered to include handwashing facilities, even if one or more households using the sanitation facility possess movable HW station.

## Knowledge of critical handwashing times

An individual has adequate knowledge of handwashing practices when they can mention the following pieces of information without prompting

- - Knowledge of critical times for handwashing: Before cooking, before eating, after using the bathroom, after changing diapers/cleaning a child who has defecated (must mention 3/4).
  - Knowledge of proper handwashing technique: use of soap (or its equivalent) and water

## Open-defecation free (ODF)

A community is considered to be open-defecation free if a competent authority has certified that all of the following conditions are met:

- Community verification and certification committees report that the community is open-defecation free
- No feces are observed in the open, particularly at former open defecation sites
- Observation at dawn and dusk does not detect anyone practicing open defecation
- Observers from neighboring communities do not detect any open defecation
- Latrines show evidence of use (see above)
- No reports of open defecation in conversations with community members, including children.

For more information, refer to the CLTS handbook: <http://www.communityledtotalsanitation.org/sites/communityledtotalsanitation.org/files/media/cltshandbook.pdf>

# Measurement procedures

## Flow rate measurement

- - Materials: 20-L graduated container with markings in liters; stopwatch
  - Manual boreholes and other sources where water is extracted via manual labor: Instruct a community member to draw water as they usually do. When water is flowing at a steady rate, begin filling the 20-L container. Measure the time required to fill the 20-liter container, in seconds. Divide 1200 by the number of seconds required to fill the container. This is the flow rate in liters per minute.
  - Mechanized, gravity-fed, and piped systems: same as above, except that the faucet should be opened completely to fill the container. Wait until the water is flowing at a steady rate, then begin filling the 20-L container. Measure the time required to fill the 20-liter container, in seconds. Divide 1200 by the number of seconds required to fill the container. This is the flow rate in liters per minute.
  - For open wells, streams, and other sources for which a flow rate cannot practically be measured, mark 333 for “not applicable”
  - For open wells, streams, and other sources for which a flow rate cannot practically be measured, mark 333 for “not applicable”

## Distance measurement

- - Materials: 25-m tape measure, GPS device
  - Distances less than 25 m may be measured using the tape measure.
  - Distances greater than 25 m should be measured using the GPS device according to the manufacturer’s instructions. Sample instructions for the Garmin E-trex 10 GPS are provided below.
    - Open the device’s Trip Computer: The trip computer displays your current speed, average speed, maximum speed, trip odometer, and other helpful statistics.
    - Clear the previous trip and start a new trip
    - Walk the distance you wish to measure
    - Record the distance from the trip odometer, in meters
    - Clear the trip.

## Travel time measurement

- Materials: stopwatch, respondent’s water container (if available)
- Start the stopwatch
- Begin walking with the survey respondent to the water point in question
- Allow the survey respondent to take the lead and walk at their normal pace, to avoid setting the pace yourself.
- Ask the respondent to fetch water as they normally would.
- Return with the respondent to their home, again allowing them to take the lead.
- Record the round-trip travel time, including fetching water and queuing.

## Water point inspection

Water point inspections should be conducted using the World Health Organization’s sanitary inspection procedures (<http://www.who.int/water_sanitation_health/dwq/wsp170805AppC.pdf>), adapted according to the source type. Example inspection questions are presented below:

Borehole with hand pump

1. Is there a latrine within 10 meters of the water point?
2. Is the nearest latrine on higher ground than the water point?
3. Is there any other source of pollution within 10 meters of the water point (e.g. animal excreta, rubbish, etc.)?
4. Is there any ponding of stagnant water within 2 meters of the cement floor of the water point?
5. Is the water point’s drainage channel broken, cracked, in need of cleaning, or not present?
6. Is there inadequate fencing around the installation, which would allow animals in?
7. Is the cement apron less than 1 meter in radius all around the water point?
8. Is there any ponding on the cement apron around the water point?
9. Are there any cracks on the cement floor around the water point?
10. Is a bucket or common container in use and left in a place where it could be contaminated?
11. Is the above-ground handpump, tap, or other water point hardware loose at the point of attachment to base (which could permit water to enter the casing)?
12. Is the cover or base of the water point visibly dirty?
13. Are the walls of the water point’s concrete pad inadequately sealed for any point for 3 meters below ground?

Total score of risks ___/13

Piped distribution system

1. Do any tapstands leak?
2. Does surface water collect around any tapstand?
3. Is the area uphill of any tapstand eroded?
4. Are pipes exposed within 10 m of any tapstand?
5. Is human excreta on the ground within 10 m of any tapstand?
6. Is there a sewer within 30 m of any tapstand?
7. Has there been a time in the last 10 days when water was not available at any tapstand?
8. Are there signs of leaks in the mains pipes feeding this system?
9. Has the community experienced any pipe breaks in the last week?
10. Is the main pipe exposed anywhere in the community?

Total score of risks ___/10

# Water Quality Testing Procedures

##

## Collection of Samples

### Materials Needed

- Sterile Whirlpak® (Nasco, Fort Atkinson, WI) bags (or sterilized autoclavable plastic bottles)
  - Where sources may have residual chlorine, Whilpak® Thiobags or sterilized bottles with added sodium thiosulfate should be used
- Cooler with ice packs
- Permanent markers
- Latex or nitrile Gloves
- Alcohol-based hand sanitizer
- Sterile distilled water (at least one fresh bottle per sample collector per day)

### Procedure for Collection of Samples

1. Label each collected sample or blank with a unique barcode ID and scan before collecting sample.
   1. If barcodes are unavailable, label with sample ID, date and time using permanent marker
2. Apply a fresh pair of gloves and sterilize hands with hand sanitizer
3. For water samples for microbiological analysis:
   1. Open labeled sterile 500-mL Whirlpak® bag without touching the lip or the inside of the bag
   2. For source samples: take sample directly from pipe, hose, or other source outlet; for household samples, ask the female head of household to bring you a glass of water just as she would normally drink or serve it, and collect the sample in the sterile sample container.
   3. Fill container with sample without touching anything to the lip or the inside of the container (no hands, pipes, glasses, or other objects of any kind)
   4. If using a Whirlpak® bag:
      1. Whirl the bag quickly three times and pinch the sides closed
      2. Twist the yellow tabs together to seal the bag shut
   5. Place sample container upright in cooler with ice
4. For Physical/Chemical Analysis samples (Arsenic and Fluoride):
   1. For on-site analysis: collect as described above and test immediately
   2. For off-site analysis: collect samples not requiring acidification (Fluoride, etc.) as described above.
   3. For samples needing to be acidified (Arsenic, etc. if not to be tested immediately on-site), add the requisite amount of acid to the containers either before or immediately after sample collection. Typically this will be 1 mL of 1 N HCl for every 100 mL of sample collected.
   4. Seal the containers
   5. Place upright in cooler with ice
5. During each day on which samples are collected, collect a field blank as follows (if a relatively small number of samples are collected each day, blanks can be collected on alternate days):
   1. Scan the barcode of the field blank.
   2. Open a fresh bottle of sterile distilled water without touching the mouth of the bottle (a reliable brand of bottled water may be used if sterile distilled water is unavailable)
   3. Fill the sample container with sterile distilled water using the same procedure as rest of samples
6. During each day on which samples are collected, collect a duplicate sample as follows (if a relatively small number of samples are collected each day, duplicates can be collected on alternate days):
   1. Scan the barcode of the duplicate sample.
   2. Collect a second sample alongside the primary sample. All sample collection and analysis procedures should be identical.

## Sample storage and transport *(For off-site analysis only)*

1. All samples should be analyzed immediately or stored inside secondary containers (clean and sterile plastic bags) in a cooler with ice packs within 15 minutes of collection. The cooler temperature should not exceed 5 degrees C. Cooler temperature should be monitored with cold chain indicator strips such as 3M Monitor Mark® or similar (if available).
2. Analyze samples on-site or transport to field lab or central laboratory for analysis within 12 hours.
3. The cold chain must remain unbroken during transport to the laboratory. If the temperature of a batch of samples exceeds 5 C for a cumulative total of one hour or more, or if the total hold time is >24 hours, discard the batch of samples.

## Chain of Custody *(For central laboratory analysis only)*

1. Every day, each field team transporting sample to a central laboratory for testing must obtain a fresh chain of custody (COC) form when they check out their cooler.
2. The top portion of the COC form must be completed by the field team and transported with all samples to the lab.
3. Any transfer of samples between field teams or between a field team and other workers must be documented on the COC form.
4. No samples can be processed until the lab technicians verify that the cold chain has not been broken and that the contents of the cooler match the COC form. Once these details have been verified, the lab technicians can sign off on the COC form to accept custody of the samples.
5. Once received by the laboratory, samples and cold chain indicators must be immediately transferred to a refrigerator and must not exceed 5 degrees C. All samples must be processed within 12 hours of the time they are received by the laboratory.
6. If the total time between collection and analysis exceeds 24 hours, or the temperature of the samples exceeds 5 degrees C for a cumulative total of one hour or more, all samples in the batch must be discarded, along with all analytical results for those samples.

# Sample Analysis

Analysis Template

| **Parameter** | **Perform for:** | **Method** | **Sample type** |
| --- | --- | --- | --- |
| *E. coli* | Core (All samples) | Compartment Bag Test | Microbiological Samples |
| Arsenic | Core (Source water samples) | ITS Arsenic Quick II Test Kit | Test on-site or else acidify samples at time of collection: 3 mL HCl/300 mL sample (pH ≤ 2) |
| Fluoride | Core (Source water samples) | Ion Selective Exlectrode (Extech FL 700) | Non-acidified samples |
| pH | Non –core | pH Meter (Hanna HI 98129) | Measure on-site |
| Turbidity | Non -core | Turbidimeter (Hanna HI 98703) | Non-acidified samples |
| Conductivity | Non -core | Conductivity Meter (Hanna HI 98129) | Measure on-site |

Field Duplicates and Blanks

| Field Duplicates | 10% of samples, at least 1/day | Reprocess a standard sample as if the duplicate were a second sample. |
| --- | --- | --- |
| Field Blanks | 10% of samples, at least 1/day | Process sterile distilled/deionized water (or a reliable brand of bottled water if sterile DI unavailable) as a normal sample. |

## Microbiological Analysis

## Compartment Bag Test (Source: Aquagenx LLC)

1. Pour 100 mL of sample into sample bottle
2. Mix water sample with growth medium by dispensing the Hi E. coli Test Bud into the sample water (without touching the Test Bud with hands or fingers). Put the sample bottle lid on and allow 15 minutes for the medium to dissolve; periodically swirl to mix. Note: The medium dissolves from the Test Bud, leaving the Test Bud in the sample. Test Bud turns white in color once medium has dissolved into the sample.
3. Open and fill the CBT with the 100mL water sample with dissolved medium, and take care to leave the Test Bud in the bottle. Use one or both of the white tabs at the top of the bag to hold the bag open while pouring in the sample with dissolved medium. Manually adjust the volumes in each compartment to their fill mark. All water levels should be even (at the same level) in all compartments.
4. Seal the filled compartment bag to isolate the individual compartments of water using a two-piece spring clip. To seal, place the U-shaped part of the clip against the back of the bag, above the water levels in the compartments but below the top openings of the compartments, to ensure the compartments stay full. Snap the rod-shaped part of the clip on the front of the bag into the back of the clip to lock it in place.
5. Incubate the sealed compartment bag for bacterial growth by placing it in an incubator or holding at ambient temperature overnight. Incubation time and temperature recommendations: For temperatures of 35—44.5°C, incubate at 20—24 hours; for temperatures 30—35°C, incubate at 24—30 hours; and for temperatures of 25—30°C, incubate overnight again (a total of 40—48 hours). For temperatures below 25°C, an incubator is recommended.
6. Score and record results: Examine the cultured water in each separate compartment of the incubated bag for the presence (positive/blue) or absence (negative/yellow) of the distinctive color change (blue or blue-green) and record the results for water volumes of each compartment as positive or negative. See the MPN chart for outcomes of positive and negative results to determine the MPN concentration of E. coli for the positive and negative compartment volumes of your sample. Record the MPN result of this sample in a suitable location.
7. Decontaminate by opening the CBT and adding 3 chlorine tablets. Agitate the bag periodically until the chlorine tablets fully dissolve and let bag stand for 45 minutes. After 45 minutes, pour liquid contents into a sink, toilet or hole in the ground and safely dispose of the empty bag. Retain plastic clip for reuse.


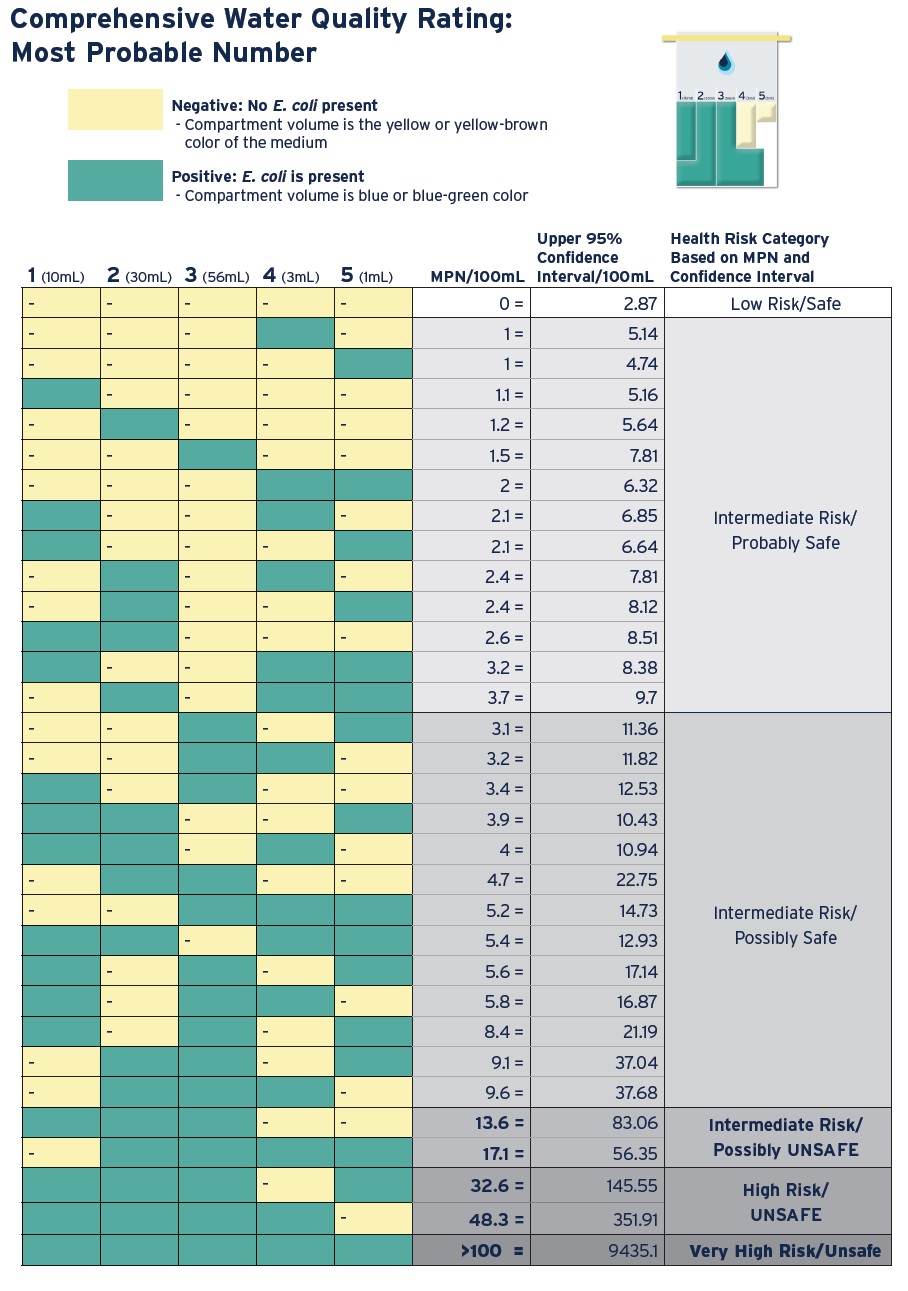


## Physical/Chemical Analysis

###

### Core Parameters Measured in the Field

### Arsenic

All samples must be analyzed via EPA method 7061 (AAS), A rapid test method such as ITS Arsenic Quick™ Kit, or comparable method may also be used if adequately validated. Note: the method used will depend greatly on the instrumentation available to the analytical laboratory or field team.

## Field-based ITS Arsenic Quick™ Kit Method (Source: Industrial Test Systems, Inc.)

#### MATERIALS

ITS Arsenic Quick™ Kit, including:

2 Reaction Bottles, clear PVC, with 20ml (lower) and 100ml (upper) lines with yellow caps

2 White Caps, with white turret, for holding test strip

• 3 Plastic Spoons (one large pink spoon for First Reagent; one small red spoon for Second

Reagent; and one small white spoon for Third Reagent)

• Easy-Read™ Color Chart

##### REAGENTS

• 1 Large Bottle of First Reagent (395gm)

• 3 Small Bottles of Second Reagent (26gm ea.)

• 5 Small Bottles of Third Reagent (28gm ea.)

• Arsenic Strips

#### SAMPLE COLLECTION, PRESERVATION, AND HANDLING

1. All samples must have been collected according to the sampling plan described above.
2. If Whirlpak disposable sample bags are used, no cleaning is required. If reusable sample containers are used, all sample containers must be prewashed with detergents, acids, and water. Plastic and glass containers are both suitable.
3. Aqueous samples must be acidified to a pH of < 2 with reagent-grade Hydrochloric acid (or similar) at the time of collection (approximately 1 mL of acid for every 100 mL of sample) if analysis is not performed on-site.

#### PROCEDURE

For best results, the water temperature should be between 22°C to 28°C / 72ºF - 84ºF .

1. Use a thermometer to verify the temperature of the sample.

2. To the Reaction Bottle, slowly add the water sample to the marked line on the bottle (100 mL).

3. Add 3 level pink spoonfuls of the First Reagent to the Reaction Bottle. Cap the bottle securely with the yellow mixing cap and shake vigorously for **15 seconds**.

4. Uncap the Reaction Bottle; add 3 level red spoonfuls of the Second Reagent . Cap the bottle securely with yellow mixing cap and shake vigorously with bottle upright for **15 seconds**. Allow the sample to sit for **2 minutes** to minimize Sulfide interference.

5. Uncap the Reaction Bottle and add 3 level white spoonfuls of the Third Reagent . Cap the bottle securely with yellow mixing cap and shake vigorously for **5 seconds**.

6. Remove yellow mixing cap. Recap the bottle immediately and securely using the white cap (must be dry) with turret up (open).

7. Remove one Arsenic test strip from the test strip bottle and immediately recap the test strip bottle. In order for the results to be accurate, the test strip must be oriented correctly, and inserted to the correct depth. Insert the test strip into the turret as illustrated in Figure 1 and Figure 2:


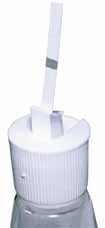

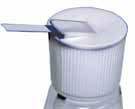


Figure 1 Figure 2

a) Position the strip so that the test pad and red line are facing the back of the white cap (see Figure 1).

b) Insert the strip into the turret until the red line is even with the top of the turret, and close (flip down) the turret (see Figure 2). This will hold the test strip in place. *(****Note:*** *Steps 6 & 7 should be completed within* ***30 seconds****.)*

8. Using a timer, allow the reaction to occur in an undisturbed, well-ventilated area for 10 minutes. Reaction generates small hydrogen gas bubbles.

9. After the 10 minute wait (but before 12 minutes), pull up the turret and carefully remove the test strip. Do not touch the reaction pad. Use the Quick™ Easy-Read™ Color Chart to match the reaction pad color.

Position the reacted test strip pad behind the punched holes, view center of test strip pad through the hole, and confirm precise color match and Arsenic level. COMPLETE MATCHING IMMEDIATELY (WAIT NO LONGER THAN 30 SECONDS). After 30 seconds have elapsed, the colors begin to change (yellow colors fade and browns turn grey or black). For best color matching results use natural daylight; avoid direct sunlight.

10. Record your result.

NOTE: Arsenic test strips will not work if they are wet! Be sure to keep strips dry!

***NOTE:*** If your Arsenic level is 200 ppb or above, you can confirm the elevated levels by diluting the water sample 1 to 5. Fill the Reaction Bottle to the bottom marked line with water sample (20 mL) and add Arsenic-free deionized or distilled water to the upper marked line. Now run steps 3 through 10. For your true Arsenic value, multiply the result by 5 to correct for dilution and record the value.

***ATTENTION:*** Soon after testing is completed, decant liquid from the bottle down a drain that is not used for food preparation and flush with water. Wet Zinc should be collected and disposed of according to local regulations. Rinse the bottle, white cap, and yellow cap with clean deionized or distilled water. Shake off any excess water and dry the white cap with turret with a soft tissue. Drying the white turret cap is especially important if you plan to run the next test immediately. Store the used strips in the plastic bag marked "Used Mercuric Bromide (HgBr2) Test Strips". Keep the used strips inaccessible to children and pets, and dispose according to local environmental regulations.

**1.** To gain confidence in using this test kit for unknown samples, it is highly recommended that you use the kit on a sample with a known inorganic Arsenic concentration value, or with a sample that has been prepared using an Arsenic standard. By making a “practice run” of the test, you will familiarize yourself with all of the procedures necessary to ensure accurate testing results. Additionally, you will have the opportunity to become familiar with the process of color matching, which will help to ensure accurate test results. ITS suggests the test be run in duplicate for better accuracy.

**2.** The water sample must not be preserved with Nitric Acid or any other preservation method. Small amounts of strong acids will interfere with the test results; and therefore it is best that the water sample be freshly drawn and run within 24 hours. Some water samples held for over 24 hours may read low. The water sample should not contain any significant amount of buffers. If you are planning to send a duplicate sample for ICP laboratory verification, follow preservation requirements for that sample only.

**3.** The water and ambient temperature are very important to ensure accurate results. As an example, a water temperature of 15°C / 59ºF can result in the color development on the testing pad to be lighter than the actual Arsenic concentration in the tested sample (a false low reading occurs). When the water is cold, warm water sample to 22°C to 28°C / 72ºF - 84ºF before testing (using a microwave is acceptable). If the water temperature is above 28°C / 84ºF your result may read low (accelerator chemistry reacts too fast). Consideration must also be made for the air temperature when running the test. Best results are from 22°C to 28°C / 72ºF - 84ºF (water and air). The color chart and Arsenic Scan chart are calibrated at 24°C / 75ºF.

**4.** After the test has been run, try to rinse out the reaction bottle with clean tap water as soon as possible. When the reaction chemicals are allowed to sit in the reaction bottle after the reaction time, the zinc may begin to adhere to the bottom of the bottle. When this occurs, you may need to clean the reaction bottle with a bottlebrush. Another method for zinc removal is to use a 20% Hydrochloric Acid (reusable) rinse. Be sure to rinse the reaction bottle with clean tap water before running the next test.

**5.** When matching your test strip pad with the colors on the Easy-Read™ color chart, it may be helpful to find a color that is clearly lighter than the test strip pad and make note of it (as an example, we will use a value of 10 ppb). Next, find a color that is clearly darker than the test strip pad (as an example, we will use a value of 30 ppb). By defining a lowest and highest possible value range we can assume that the correct color match is 20 ppb. If the 20 ppb color matches, then you have determined your Arsenic level. In some cases, however, an exact color match will not be available. As an example, if your test strip pad is slightly darker than 20 ppb and slightly lighter than 30 ppb, you can estimate a value of 25 ppb as your result. Following these easy steps can make color matching more precise. Careful color matching will assure the best possible result.

**6.** Levels of Hydrogen Sulfide above 2 mg/L can interfere with this test, resulting in elevated Arsenic readings. Our test kit will eliminate up to 2 mg/L of Sulfide interference. To overcome Hydrogen Sulfide levels above 2 mg/L, allow the water sample to sit at room temperature, uncovered and exposed to air for 8 hours (about 50% of the H2S gas dissipates for every 8 hours).

CAUTION: Arsine is very toxic. Precautions must be taken to avoid inhaling arsine gas.

### Fluoride

All samples must be analyzed via EPA method 340.2 (AAS), 340.1 (colorimetry), or comparable method such as the use of a fluoride ion selective electrode, with adequate validation.

#### Ion-selective Electrode Method (Source: FLIR Systems)

#### Materials

##### Apparatus

- Extech ExStik FL700 Fluoride Meter

##### Reagents

- Sodium fluoride, stock solution: 1.0 mL = 0.1 mg F. Dissolve 0.2210 g of sodium fluoride in distilled water and dilute to 1 liter in a volumetric flask. Store in chemical-resistant glass or polyethylene.
- Sodium fluoride, standard solution: 1.0 mL = 0.01 mg F. Dilute 100.0 mL of sodium fluoride stock solution (6.2) to 1000 mL with distilled water.
- TISAB tablets or solution

#### Calibration

**Calibration**

The Fluoride Meter should be calibrated daily.

1. Prepare a 1 ppm fluoride standard and TISAB reagent, or use pre made mixed TISAB

and 1 ppm standard

2. Pour 15 – 20 mls of this standard solution into the sample cup

3. Rinse the end of the FL700 module in TISAB solution and wipe thoroughly with paper

tissue

4. Place the FL700 into the 1 ppm standard

5. Switch the instrument on using the **ON/OFF** key. The instrument will now go through its

internal calibration

6. 1.0 ppm reading will stabilizes in ~35 seconds and the instrument will enter HOLD

mode

7. When in HOLD mode press the **CAL** key and hold until 1.0 ppm and CAL appear in the

display. Release the **CAL** key

8. Wait until the display stops blinking; the instrument will enter the HOLD mode

9. The instrument is now calibrated and ready for use

#### Procedure

1. Prepare unknown solution by adding TISAB reagent to the sample in the sample cup. Thoroughly wipe

the end of the FL700

2. Place the FL700 into the prepared unknown sample

3. If the display is indicating HOLD press the **HOLD** key to enter the Measure mode (the

HOLD display will switch off)

4. After ~ 35 seconds the instrument will display the value of the unknown concentration

5. Record the results

6. Rinse the fluoride meter and sample cup with deionized/distilled water

7. Recap the fluoride meter and switch it off.

### Non-core Parameters Measured in the Field

These parameters are optional, but add significant information to the core parameters listed above.

### pH

All samples must be analyzed via EPA method 150.1 or comparable method to be approved by the Water Institute team. The field team should measure pH in the field, as this parameter can change significantly over time.

## pH meter method (Source: Hanna Instruments, Inc.)

#### Materials

- pH/EC/TDS meter and electrode
- 50-mL plastic sample cup or beaker
- pH calibration standards (pH 4.00, 7.00, 10.00)
- Wash bottle with distilled or deionized water

#### Procedure

1. Before analyzing samples, calibrate the pH meter each morning according to the manufacturer’s instructions.
2. Rinse the electrode and sample cup/beaker with distilled/deionized water
3. Fill the sample cup/beaker with an appropriate amount of sample, per the pH meter manufacturer’s instructions (usually 50 mL)
4. Select the pH mode with the SET/HOLD button.
5. Submerge the electrode in the solution to be tested while gently stirring.
6. The measurements should be taken when the reading stabilizes and the stability symbol (clock) on the top left of the LCD disappears.
7. Record the sample pH

### Conductivity/Total Dissolved Solids

All samples must be analyzed via EPA method 120.1 or comparable method to be approved by the Water Institute team. The field team should measure conductivity/TDS in the field, as this parameter can easily be measured at the same time as pH.

## Conductivity/TDS meter method (Source: Hanna Instruments, Inc.)

#### Materials

- pH/EC/TDS meter and electrode
- 50-mL plastic Sample cup or beaker
- Conductivity calibration standards (Standard potassium chloride solutions, 0.01 M: Dissolve 0.7456 gm of pre-dried [2 hour at 105°C] KCl in distilled water and dilute to 1 liter at 25°C)
- Wash bottle with distilled or deionized water

#### Procedure

1. Before analyzing samples, calibrate the TDS meter each morning according to the manufacturer’s instructions
2. Allow all samples to come to room temperature (23-27°C) if possible
3. Rinse the electrode and sample cup/beaker with distilled/deionized water
4. Fill the sample cup/beaker with an appropriate amount of sample
5. Select the TDS mode with the SET/HOLD button.
6. Submerge the electrode in the solution to be tested while gently stirring.
7. Use plastic beakers to minimize any electromagnetic interferences.
8. The measurements should be taken when the reading stabilizes and the stability symbol (clock) on the top left of the LCD disappears.
9. Record the sample TDS.

### Turbidity

All samples must be analyzed via EPA method 180.1 or comparable method to be approved by the WI team.

## Portable turbidimeter method (Source: Hanna Instruments, Inc.)

#### Materials

- Turbidimeter
- Turbidity calibration standards (0.1, 1.0, and 10.0 NTU, or similar)
- Turbidity-free water
- Wash bottle with turbidity-free distilled or deionized water

#### Procedure

1. Before analyzing samples, calibrate the turbidimeter according to the manufacturer’s instructions.
2. Rinse the sample cell with turbidity-free distilled/deionized water
3. Fill the sample cell with an appropriate amount of turbidity-free water
4. Zero the turbidimeter
5. Agitate the water sample to be measured: this will resuspend any particles that may have sedimented out.
6. Fill the sample cell with an appropriate amount of sample
7. Measure and record the sample turbidity
8. Discard the sample and rinse the sample cell with turbidity-free distilled/deionized water
