# Supplementary material for: WaSH CQI: Applying continuous quality improvement methods to water service delivery in four districts of rural northern Ghana
Source: PLoS One. 2020 Jul 15;15(7):e0233679. doi: 10.1371/journal.pone.0233679 (PMC7363065; doi:10.1371/journal.pone.0233679)

WaSH CQI: Applying Continuous Quality Improvement methods to Water Service Delivery in four districts of rural northern Ghana

Authors: Michael B. Fisher^1^*; Leslie Danquah^2^; Zakariah Seidu^3^ Allison N. Fechter^4^; Bansaga Saga^5^; Jamie K. Bartram^1^; Kaida M. Liang^1^; Rohit Ramaswamy^6^*

1. The Water Institute at UNC, Department of Environmental Sciences and Engineering, University of North Carolina at Chapel Hill, Chapel Hill, NC USA

2. School of Geosciences, University of Energy and Natural Resources, Sunyani, Ghana.

3. West African Centre for Cell Biology of Infectious Pathogens, University of Ghana, Legon, Ghana.

4. The Water Project, Concord, NH USA

5. Solidarites International, Clichy, FRANCE

6. Public Health Leadership Program, Gillings School of Global Public Health, University of North Carolina, Chapel Hill, NC USA

*Correspondence: mbfisher@gmail.com (MBF); ramaswam@email.unc.edu (RR); Tel.: +1-919-966-2480

## File S6. Supporting tables and Figures.

16 Tables

2 Figures

Table S6.1. Baseline summary statistics by treatment arm (Household)

| Variable | Intervention (n=229) | Control (n=295) | P |
| --- | --- | --- | --- |
| Mean household population | 5.62 | 5.49 | 0.4544 |
| Proportion of households with a school-aged child | 82.1% | 80.0% | 0.5456 |
| Current main water source is improved | 59.1% | 61.9% | 0.5277 |
| Dry season main water source is on-plot | 0.4% | 0.6% | 0.72 |
| Wet season main water source is on-plot | 8.4% | 10.3% | 0.51 |
| Current main water source is continuously available | 70.4% | 80.2% | 0.0092** |
| Current main water source has failed in the past 2 weeks | 40.3% | 32.8% | 0.3874 |
| Stored water at time of visit is from an improved source | 65.0% | 70.1% | 0.2225 |
| Detectable *E. coli* in stored water | 83.2% | 86.9% | 0.2444 |
| Storage container visibly clean | 6.1% | 4.4% | 0.3817 |
| Storage container covered | 59.4% | 57.3% | 0.6295 |
| Storage container has narrow (<15 cm) opening | 10.0% | 8.5% | 0.5377 |
| Storage container out of the reach of animals | 53.3% | 60.1% | 0.0894 |
| HH pays to fetch water | 32.3% | 27.8% | 0.2628 |
| HH treats water | 24.6% | 16.8% | 0.0298* |
| A child under 5 has suffered from diarrhea in the last 2 weeks | 39.2% | 37.3% | 0.6629 |
| A child under 5 has missed school due to illness in the last 2 weeks | 32.3% | 26.4% | 0.1878 |
| A child under 5 has died in the last year | 12.2% | 14.6% | 0.4367 |
| Human excreta observed in home | 4.8% | 5.8% | 0.6290 |

*Results significant at 95% confidence level **Results significant at 99% confidence level

Table S6.2. Baseline summary statistics by treatment arm (Water Source)

| Variable | Intervention (n=217) | Control (n=271) | P |
| --- | --- | --- | --- |
| Average age of water source (y) | 10 | 10 | 0.6846 |
| Average number of households using each water source | 50.9 | 51.9 | 0.8552 |
| Water source functional | 66.6% | 62.3% | 0.1781 |
| Borehole functional | 68.0% | 64.0% | 0.3650 |
| Water source has failed in the last 2 weeks | 37.3% | 39.3% | 0.5457 |
| Water source has failed in the last year | 57.4% | 50.8% | 0.1054 |
| Borehole has failed in the last 2 weeks | 32.5% | 33.8% | 0.7662 |
| Broken-down borehole repaired within 7 days | 49.7% | 46.0% | 0.4586 |
| Average water source flow rate (L/min) | 24.6 | 24.7 | 0.9413 |

Table S6.3. Baseline households who report treating water: treatment method

| Treatment Method | Control | Intervention |
| --- | --- | --- |
| Add bleach/chlorine | 5 | 5 |
| Use a water filter (ceramic, sand, composite) | 9 | 11 |
| Boil | 2 | 2 |
| Strain it through a cloth | 33 | 38 |

Table S6.4. Baseline sample size calculations

1. Functionality

| Functional Water Points | |  |  |  |
| --- | --- | --- | --- | --- |
|  |  |  |  |  |
| Assumptions |  |  |  |  |
| 70% | water points functional | |  |  |
| 10% | percentage point increase in proportion of functional water points | | | |
| 0% | loss to follow-up | |  |  |
| 15% | ICC for water points in same community* | | |  |
| 60% | Autocorrelation coefficient for water points in the same community | | | |
|  |  |  |  |  |
| 1 | Sampling rounds per phase | | |  |
| 2.7 | water points per community | | |  |
| 110 | communities (per arm) | |  |  |
| 297 | water points total (per arm) | | |  |
| 1.26 | design effect | |  |  |
| 236.6533865 | water points effective accounting for loss to follow-up | | | |
| 236.6533865 | data points effective accounting for autocorrelation | | | |
|  |  |  |  |  |
| Alpha (one-sided) | p1 | p2 | n1 | n2 |
| **0.05** | **70%** | **80%** | **236.6533865** | **236.6533865** |
|  |  |  |  |  |
| prop.n2 | 1 |  |  |  |
| Pbar | 0.75 |  |  |  |
| p1-p2 | 0.1 |  |  |  |
| sd.null | 0.03980698 |  |  |  |
| sd.alt | 0.03954071 |  |  |  |
| Zalpha | 1.64485363 |  |  |  |
| Zbeta | -0.8731086 |  |  |  |
|  |  |  |  |  |
| POWER | 0.80869807 |  |  |  |

*Conservative assumption based on preliminary data from this work, as well as data from Fisher, Michael B., et al. "Understanding handpump sustainability: determinants of rural water source functionality in the Greater Afram Plains region of Ghana." *Water resources research* 51.10 (2015): 8431-8449.

1. Stored Water Quality

| Stored Water Samples Positive for E.coli | | |  |  |
| --- | --- | --- | --- | --- |
|  |  |  |  |  |
| Assumptions |  |  |  |  |
| 80% | stored water samples positive for E.coli | | |  |
| 20% | reduction in proportion of stored water samples positive for E.coli | | | |
| 10% | loss to follow-up | |  |  |
| 15% | ICC for HHs in same community** | | |  |
| 60% | Autocorrelation coefficient for water points in the same community | | | |
|  |  |  |  |  |
| 1 | Sampling rounds per phase | |  |  |
| 6 | HHs per community | |  |  |
| 110 | communities (per arm) | |  |  |
| 40% | Proportion of communities in which household data are collected (random subset) | | | |
| 44 | communities (per arm) | |  |  |
| 264 | HHs total (per arm) | |  |  |
| 1.75 | design effect | |  |  |
| 135.7714286 | HHs effective accounting for loss to follow-up | | | |
| 135.7714286 | data points effective accounting for autocorrelation | | | |
|  |  |  |  |  |
| Alpha (one-sided) | p1 | p2 | n1 | n2 |
| **0.05** | **80%** | **64%** | **135.7714286** | **135.7714286** |
|  |  |  |  |  |
| prop.n2 | 1 |  |  |  |
| pbar | 0.72 |  |  |  |
| p1-p2 | 0.16 |  |  |  |
| sd.null | 0.05449493 |  |  |  |
| sd.alt | 0.05362295 |  |  |  |
| zalpha | 1.95996398 |  |  |  |
| zbeta | -0.9919616 |  |  |  |
|  |  |  |  |  |
| POWER | 0.83939187 |  |  |  |
|  |  |  |  |  |

**Conservative assumption based on our preliminary data from this work and other studies in Ghana, as well as on ICC estimates used in Huda TMN, Unicomb L, Johnston RB, Halder AK, Yushuf Sharker MA, Luby SP. Interim evaluation of a large scale sanitation, hygiene and water improvement programme on childhood diarrhea and respiratory disease in rural Bangladesh. Soc Sci Med. 2012;75: 604–611. pmid:22197292

Table S6.5. Proportion of WaSH committees reporting that they have all necessary tools for routine water system maintenance by treatment group

| Time point | Baseline | Midline | Endline (tools delivered) |
| --- | --- | --- | --- |
| Intervention | 55% (90) | 36% (70) | 79% (n=96) |
| Control | 58% (100) | 47% (76) | 43% (n=93) |
| P (t-test) | 0.6885 | 0.1559 | 0.000** |

*Results significant at 95% confidence level **Results significant at 99% confidence level

Table S6.6. Proportion of WaSH committees that reported holding a meeting in the past 30 days

| Time point | Baseline | Midline | Endline |
| --- | --- | --- | --- |
| Intervention | 48% (89) | 69% (84) | 49% (n=104) |
| Control | 51% (103) | 56% (93) | 44% (n=119) |
| P (t-test) | 0.6661 | 0.0727 | 0.4271 |

*Results significant at 95% confidence level **Results significant at 99% confidence level

Table S6.7. Proportion of household water samples in high-risk category by source type and storage type

| Time point | Baseline | Midline | Endline |
| --- | --- | --- | --- |
| Safe storage improved | N/A | 27% (n=152) | 23% (n=87) |
| All others | 53% | 48% (n=271) | 42% (n=324) |
| P (t-test) | N/A | 0.0000** | 0.0011** |

*Results significant at 95% confidence level **Results significant at 99% confidence level

Table S6.8. Multivariable logistic regression of borehole functionality in intervention vs control communities controlling for district, week, and number of users per waterpoint n=999 across 3 monitoring rounds)

| Variable | Odds Ratio | P>z | 95% CI |
| --- | --- | --- | --- |
| Water points per community (+1) | 0.8521 | 0.000** | 0.812-0.895 |
| Seasonality (Seasonal Unavailability) | 0.1403 | 0.000** | 0.0787-0.250 |
| Savings balance | 1.266 | 0.181 | 0.896-1.790 |
| Tools | 1.530 | 0.015** | 1.086-2.156 |
| Parts | 1.266 | 0.418 | 0.715-2.240 |

*Results significant at 95% confidence level **Results significant at 99% confidence level

Table S6.9. Water source functionality at baseline

| Variable | All sources | Boreholes | Piped sources | WV sources |
| --- | --- | --- | --- | --- |
| % of water points that are functional (source survey) | 69.30% | 65.88% | 57.46% | 77% |
| % of water points functional on any day in past year (Nonworking) | 71.52% | 53.35% | 93% | 68% |
| % of water points that have failed in last 2 weeks (source survey) | 38.62% | 33.55% | 50.76% | 22.68% |
| % of water points that have failed in last year (source survey) | 54.33% | 55.89% | 47.01% | 45.88% |
| % of water points with continuous supply (source survey) | 53.23% | 64.94% | 46.97% | 73.33% |
| % of water points <2 years old that are functional | 68.75% | 83.33% | 55.56% | N/A |
| % of water points >5 years old that are functional | 71.61% | 65.27% | N/A | 78.13% |
| % of water points >10 years old that are functional | 66.67% | 61.82% | N/A | 75.71% |
| % of water points >20 years old that are functional | N/A | 58.16% | N/A | N/A |

Table S6.10. Community Summary Statistics at baseline

| Variable | Value | n |
| --- | --- | --- |
| % of communities certified as ODF | 2.1% | 244 |
| % of communities with a tariff | 38.1% | 244 |
| % of communities with savings | 58.8% | 238 |
| % of communities with savings balance > USD 100 | 37.9% | 116 |
| % of communities with records up to date | 8.23% | 243 |
| % of communities with a WaSH committee | 95.9% | 244 |
| % of Watsans that have met in the last 30 days (com_watsan_meet) | 51.1% | 225 |
| % of Watsans with >2 members trained (com_mnumtrain>2) | 70.5% | 190 |
| % of Watsans that have both met in last 30 days and have >2 members trained | 32.4% | 210 |
| Median Number of years since WaSH committee was trained | 4 | 186 |
| Mean time to repair water point | 44.4 | 180 |
| % of committees reporting waterpoints repaired within 7 days | 64.4% | 180 |
| % of committees reporting support comes within 7 days | 91.4% | 139 |
| % of committees reporting spare parts available | 88.9% | 189 |
| % of committees reporting access to all required tools | 57.1% | 212 |

Table S6.11. Water Source Summary Statistics at baseline

| Variable | Percent | N |
| --- | --- | --- |
| Water within 4 strokes (handpumps) | 74.43% | 305 |
| Median Flow rate | 19.05 | 380 |
| Flow Rate > 14L/min | 77.63% | 380 |
| % of waterpoints with <300 users | 76.94% | 915 |
| % of communities certified as ODF | 2.05% | 244 |
| % of communities with a Tariff | 38.11% | 244 |
| % of communities with savings | 58.82% | 238 |
| % of communities with savings balance > USD 100 | 37.93% | 116 |
| % of communities with records up to date | 8.23% | 243 |
| % of communities with a WaSH committee | 95.90% | 244 |
| % of Watsans that have met in the last 30 days (com_watsan_meet) | 51.11% | 225 |
| % of Watsans with >2 members trained (com_mnumtrain>2) | 70.53% | 190 |
| % of Watsans that have both met in last 30 days and have >2 members trained | 32.38% | 210 |
| Median Number of years since WaSH committee was trained | 4 | 186 |
| Mean time to repair water point | 44.42 | 180 |
| % of committees reporting waterpoints repaired within 7 days | 64.44% | 180 |
| % of committees reporting support comes within 7 days | 91.37% | 139 |
| % of committees reporting spare parts available | 88.89% | 189 |
| % of committees reporting access to all required tools | 57.08% | 212 |
| % of water sources in compliance with E. coli standards (0 CFU/100 mL) | 40.11% | 566 |
| % of water sources in High-risk category for E. coli (>100 CFU/100 mL) | 36.00% | 566 |

Table S6.12. Multivariable regression: determinants of water source functionality at baseline (boreholes with handpumps)

|  | Odds Ratio | P>z | [95% Conf. | Interval] |
| --- | --- | --- | --- | --- |
| District |  |  |  |  |
| Gushiegu | 1 |  |  |  |
| Karaga | 0.164752 | 0.004** | 0.048067 | 0.564693 |
| Savelugu | 0.788262 | 0.561 | 0.353748 | 1.756496 |
| Tolon | 0.810508 | 0.635 | 0.34042 | 1.929741 |
| Water source age | 1.02686 | 0.368 | 0.969262 | 1.087881 |
| Number of water points | 0.736547 | 0.000** | 0.650725 | 0.833687 |
| All tools available | 1.430125 | 0.236 | 0.790907 | 2.585967 |
| Spare parts available | 0.943885 | 0.898 | 0.390793 | 2.279774 |
| Committee has records | 1.132671 | 0.673 | 0.63479 | 2.021052 |
| Committee has savings > USD 100 | 1.82107 | 0.064 | 0.966841 | 3.430032 |
| Community population (+1 person) | 1.000207 | 0.055 | 0.999996 | 1.000418 |
| Who provides external support? |  |  |  |  |
| Area Mechanic | 1 |  |  |  |
| District/Local Government | 0.607262 | 0.328 | 0.223748 | 1.648135 |
| NGO | 0.562044 | 0.158 | 0.25243 | 1.251411 |
| No one | 0.225279 | 0.008** | 0.075346 | 0.673561 |
| Private maint. Person | 0.71733 | 0.482 | 0.284204 | 1.810538 |

*Significant at 95% Confidence Level (CL) ** Significant at 99% CL

Table S6.13. Determinants of household stored water quality at baseline

|  | Odds Ratio | P>z | [95% Conf. | Interval] |
| --- | --- | --- | --- | --- |
| District* |  |  |  |  |
| Gushiegu | 1 |  |  |  |
| Karaga | 44.76 | 0.069 | 0.743-2694.4 |  |
| Savelugu | 2.33E7 | 0.993 | 0-. |  |
| Tolon | 1.5E7 | 0.994 | 0-. |  |
| Source Type*** |  |  |  |  |
| 3 | 1 |  |  |  |
| 4 | 3.45E-8 | 0.985 | 0 | . |
| 5 | 1 |  |  |  |
| 6 | 4.956 | 0.008** | 1.530-16.053 |  |
| 7 | 0.687 | 0.415 | 0.278-1.695 |  |
| 9 | 2.509 | 0.086 | 0.877-7.181 |  |
| 11 | 1.414 | 0.608 | 0.376-5.313 |  |
| 15 | 1 |  |  |  |
| Water storage container with lid | 0.468 | 0.047* | 0.222-0.988 |  |
| Where children defecate | 3.657 | 0.004** | 1.505-8.881 |  |
| 1 | 1 |  |  |  |
| 16 | 2.452 | 0.576 | 0.106-56.80 |  |
| 20 | 0.587 | 0.671 | 0.050-6.856 |  |
| 22 | 1.667 | 0.707 | 0.116-23.91 |  |
| Household latrine | 0.294 | 0.011* | 0.115-0.752 |  |
| Hand wash with soap | 0.667 | 0.233 | 0.344-1.296 |  |
| Hand wash with rubbing | 0.137 | 0.000** | 0.050-0.379 |  |
| Soap present for hand wash | 0.665 | 0.033* | 0.458-0.970 |  |
| Household size bin | 1.760 | 0.084 | 0.928-3.334 |  |
| Week |  |  |  |  |
| 1 | 8.31E-07 | 0.987 | 0-. |  |
| 2 | 2.44E-06 | 0.988 | 0-. |  |
| 3 | 1.30E-06 | 0.988 | 0-. |  |
| 4 | 2.53E-07 | 0.986 | 0-. |  |
| 5 | 3.14E-08 | 0.984 | 0-. |  |
| 6 | 3.79E-07 | 0.987 | 0-. |  |
| 7 | 1.88E-07 | 0.986 | 0-. |  |
| 8 | 3.33E-08 | 0.985 | 0-. |  |
| 9 | 7.25E-08 | 0.985 | 0-. |  |
| 10 | 1.18E-07 | 0.986 | 0-. |  |
| 11 | 12.41196 | 0.999 | 0-. |  |
| 12 | 9.919471 | 0.999 | 0-. |  |
| 13 | 8.426531 | 0.999 | 0-. |  |
| 14 | 1 |  |  |  |
| 15 | 0.904803 | 1 | 0-. |  |
| 16 | 2.617248 | 1 | 0-. |  |
| 17 | 3.514334 | 1 | 0-. |  |
| 18 | 4.285177 | 0.999 | 0-. |  |
| 19 | 3.758114 | 1 | 0-. |  |
| 20 | 1 |  |  |  |
| 21 | 0.327322 | 1 | 0-. |  |
| 22 | 17.78387 | 0.999 | 0-. |  |
| 23 | 0.086382 | 0.999 | 0-. |  |
| 24 | 1 |  |  |  |

*Significant at 95% Confidence Level (CL) ** Significant at 99% CL

Table S6.14. Household Stored Water Quality vs. Presence of Safe Water Storage Container

| Safe water storage container present | Yes | No | P |
| --- | --- | --- | --- |
| Detectable *E. coli* (>= 1 CFU/100 mL) in household stored water | 28.9% (204) | 16.9% (249) | 0.0021** |

*Significant at the 95% CI **Significant at the 99% confidence interval

Table S6.15. Water Source Functionality (Improved sources only)

| Community | Intervention | Control | P |
| --- | --- | --- | --- |
| Proportion of improved sources functional on the day of the visit | 68.1% (110) | 55.0% (129) | 0.04* |

*Significant at the 95% CI **Significant at the 99% confidence interval

Table S6.16. Comparison of selected descriptive statistics between treatment arms and monitoring rounds.

| Variable | Baseline | | | Endline | | |
| --- | --- | --- | --- | --- | --- | --- |
|  | Intervention | Control | All | Intervention | Control | All |
| HH size (median) | 5 | 5 | 5 | 6 | 6 | 6 |
| Compound size (median) | 14 | 15 | 15 | 14.5 | 15 | 15 |
| Number of children <5 (mean) | 1.68 | 1.78 | 1.73 | 1.41 | 1.68 | 1.56 |
| Has children in school | 82.1% | 80.0% | 80.9% | 79.7% | 82.9% | 81.4% |
| Main source (dry season) | Borehole 52.8%  Tap 10.9%  Dug well: 1.7%  Surface Water: 30.6% | Borehole 67.1%  Tap 4.4%  Dug well 4.7%  Surface Water: 20.7% | Borehole 60.9%  Tap 7.3%  Dug well: 3.4%  Surface Water: 25.0% | Borehole 69.5%  Tap 15.2%  Dug well: 0%  Surface Water: 13.2% | Borehole 65.8%  Tap 9.8%  Dug well: 0.9%  Surface Water: 23.5% | Borehole 67.5%  Tap 12.3%  Dug well: 0.5%  Surface Water: 18.8% |
| Main source (wet season) | Borehole 38.2%  Tap 12.7%  Rainwater: 8.8%  Dug well: 15.8%  Surface Water: 20.6% | Borehole 49.8%  Tap 4.7%  Rainwater: 8.1%  Dug well: 18.0%  Surface Water: 17.6% | Borehole 44.8%  Tap 8.2%  Rainwater: 8.4%  Dug well: 17.0%  Surface Water: 18.9% | Borehole 44.2%  Tap 14.7%  Rainwater: 9.1%  Dug well: 15.8%  Surface Water: 18.3% | Borehole: 51.2%  Tap: 6.0%  Rainwater: 6.0%  Dug well: 18.0%  Surface Water: 22.25% | Borehole: 48.0%  Tap: 10.0%  Rainwater: 7.4%  Dug well: 17.0%  Surface Water: 20.4% |
| Most recent source | Borehole 41.6%  Tap 13.7%  Rainwater: 4.0%  Dug well: 12.8%  Surface Water: 23.5% | Borehole 54.6%  Tap 5.1%  Rainwater: 4.1%  Dug well: 13.7%  Surface Water: 19.5% | Borehole 48.9%  Tap 8.9%  Rainwater: 4.0%  Dug well: 13.3%  Surface Water: 21.2% | Borehole 66.5%  Tap 15.2%  Surface Water: 16.2% | Borehole 61.5%  Tap 11.1%  Surface Water: 24.8% | Borehole 63.8%  Tap 13.0%  Surface Water: 20.9% |
| Time to get water (median) | 6 min | 6 min | 6 min | 13.5 min | 14 min | 14 min |
| Water per-capita (median L) | 24.2 | 21.3 | 22.9 | 23.6 | 24.2 | 24.0 |
| Wealth quintile (mean) | 3.16 | 2.86 | 3.00 | 3.07 | 2.91 | 2.99 |
| Respondent’s level of Education | No school: 89.5%  Some Primary: 6.1%  Completed Primary: 4.4% | No school: 86.4%  Some Primary: 6.8%  Completed Primary: 5.4% | No school: 87.8%  Some Primary: 6.5%  Completed Primary: 5.0% | No school: 87.3%  Some Primary: 7.6%  Completed Primary: 4.1% | No school: 88.9%  Some Primary: 6.8%  Completed Primary: 4.3% | No school: 88.2%  Some Primary: 7.2%  Completed Primary: 4.2% |
| Respondent’s age (median) | 30 | 30 | 30 | 35 | 35 | 35 |

Figures

Figure

Figure S6.1 CQI process Map


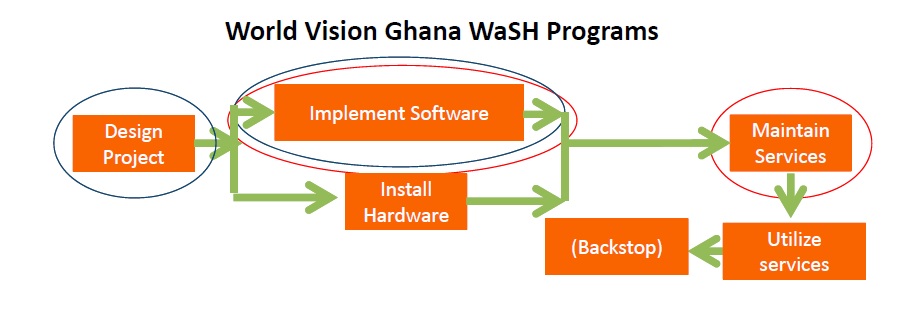


Figure S6.2. Traditional Water Storage Container in Study Setting


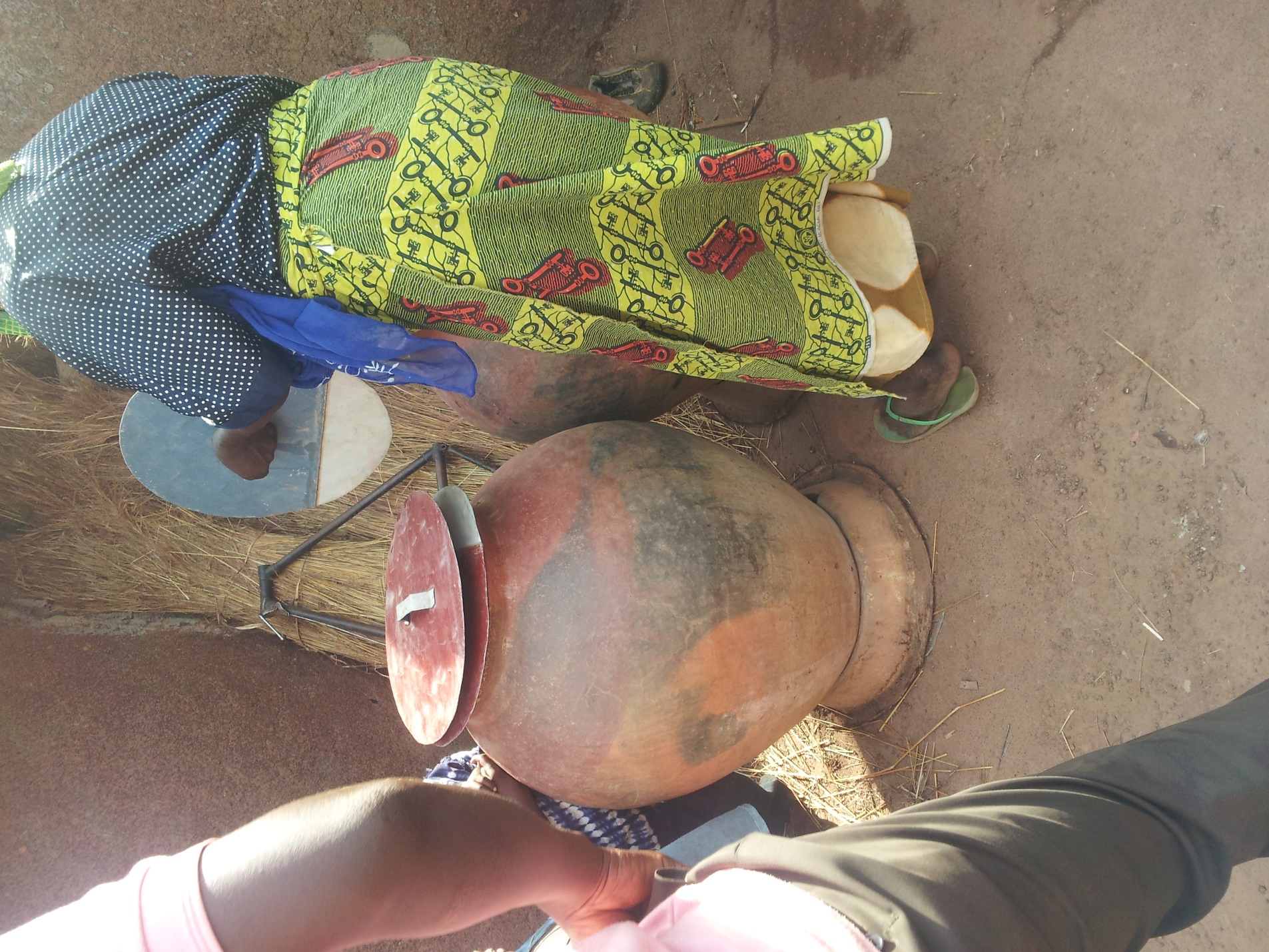

Supplement: S6 File — (DOCX) [file pone.0233679.s006.docx]
