# Supplementary material for: WaSH CQI: Applying continuous quality improvement methods to water service delivery in four districts of rural northern Ghana
Source: PLoS One. 2020 Jul 15;15(7):e0233679. doi: 10.1371/journal.pone.0233679 (PMC7363065; doi:10.1371/journal.pone.0233679)

WaSH CQI: Applying Continuous Quality Improvement methods to Water Service Delivery in four districts of rural northern Ghana

Authors: Michael B. Fisher^1^*; Leslie Danquah^2^; Zakariah Seidu^3^ Allison N. Fechter^4^; Bansaga Saga^5^; Jamie K. Bartram^1^; Kaida M. Liang^1^; Rohit Ramaswamy^6^*

1. The Water Institute at UNC, Department of Environmental Sciences and Engineering, University of North Carolina at Chapel Hill, Chapel Hill, NC USA

2. School of Geosciences, University of Energy and Natural Resources, Sunyani, Ghana.

3. West African Centre for Cell Biology of Infectious Pathogens, University of Ghana, Legon, Ghana.

4. The Water Project, Concord, NH USA

5. Solidarites International, Clichy, FRANCE

6. Public Health Leadership Program, Gillings School of Global Public Health, University of North Carolina, Chapel Hill, NC USA

*Correspondence: mbfisher@gmail.com (MBF); ramaswam@email.unc.edu (RR); Tel.: +1-919-966-2480

## File S7. Structured Decision-Making Tools

1 Table

1 Figure

Table S7.1. Pugh Matrix used to assess potential improvements against selected criteria

|  | Concept | 1 | 2 | 3 | 4 | Reference |
| --- | --- | --- | --- | --- | --- | --- |
| Criterion | Weight | Procure additional Tool kits | Train WaSH committees: repair skills | Monitor and repair boreholes quarterly | Rebuild WaSH committees to full 11 members | Semi-annual toolkit inspection |
| Ease | 4 | - | - | - | - |  |
| Speed | 3 | - | + | - | - |  |
| Cost | 8 | - | - | - | - |  |
| Sustainability/  Scalability | 9 | - | S | - | + |  |
| Impact | 10 | + | + | + | + |  |
|  | Total - | 4 | 2 | 4 | 3 |  |
|  | Total + | 1 | 2 | 1 | 2 |  |
|  | Total S | 0 | 1 | 0 | 0 | 5 |
|  | Weighted - | 24 | 12 | 24 | 15 |  |
|  | Weighted + | 10 | 13 | 10 | 19 |  |
|  | Weighted Score | -14 | **1** | -14 | **4** | 0 |

Figure S7.1. Pugh matrix used to assess potential improvements against selected criteria


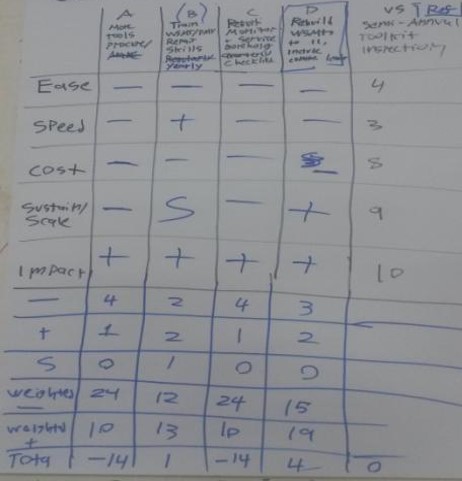

Supplement: S7 File — (DOCX) [file pone.0233679.s007.docx]
