# Supplementary material for: WaSH CQI: Applying continuous quality improvement methods to water service delivery in four districts of rural northern Ghana
Source: PLoS One. 2020 Jul 15;15(7):e0233679. doi: 10.1371/journal.pone.0233679 (PMC7363065; doi:10.1371/journal.pone.0233679)
Supplement: S8 File — (DOCX) [file pone.0233679.s008.docx]

WaSH CQI: Applying Continuous Quality Improvement methods to Water Service Delivery in four districts of rural northern Ghana

Authors: Michael B. Fisher^1^*; Leslie Danquah^2^; Zakariah Seidu^3^ Allison N. Fechter^4^; Bansaga Saga^5^; Jamie K. Bartram^1^; Kaida M. Liang^1^; Rohit Ramaswamy^6^*

1. The Water Institute at UNC, Department of Environmental Sciences and Engineering, University of North Carolina at Chapel Hill, Chapel Hill, NC USA

2. School of Geosciences, University of Energy and Natural Resources, Sunyani, Ghana.

3. West African Centre for Cell Biology of Infectious Pathogens, University of Ghana, Legon, Ghana.

4. The Water Project, Concord, NH USA

5. Solidarites International, Clichy, FRANCE

6. Public Health Leadership Program, Gillings School of Global Public Health, University of North Carolina, Chapel Hill, NC USA

*Correspondence: mbfisher@gmail.com (MBF); ramaswam@email.unc.edu (RR); Tel.: +1-919-966-2480

File S8. Final Improvement Package.

The Ghana WaSH CQI team met in June of 2014 to review the preliminary pilot monitoring data and identify improvement interventions to address the two improvement goals of the pilot: Increasing water source functionality and improving household water quality. Upon analysis of the data, the following interventions were identified as an initial improvement package:

- Distribution of safe water storage containers to reduce secondary microbial contamination of stored water at the household level.
- Refresher training and reactivation of WaSH committees in intervention communities
- Assessment of WaSH committee toolkits and replacement of missing tools

Both interventions evolved and developed over the course of the pilot. Full descriptions of these interventions are presented below.

# Safe water storage containers

Two varieties of safe water storage containers were piloted in Ghana: A locally produced plastic container, and a locally produced ceramic container. Both are described below.

| **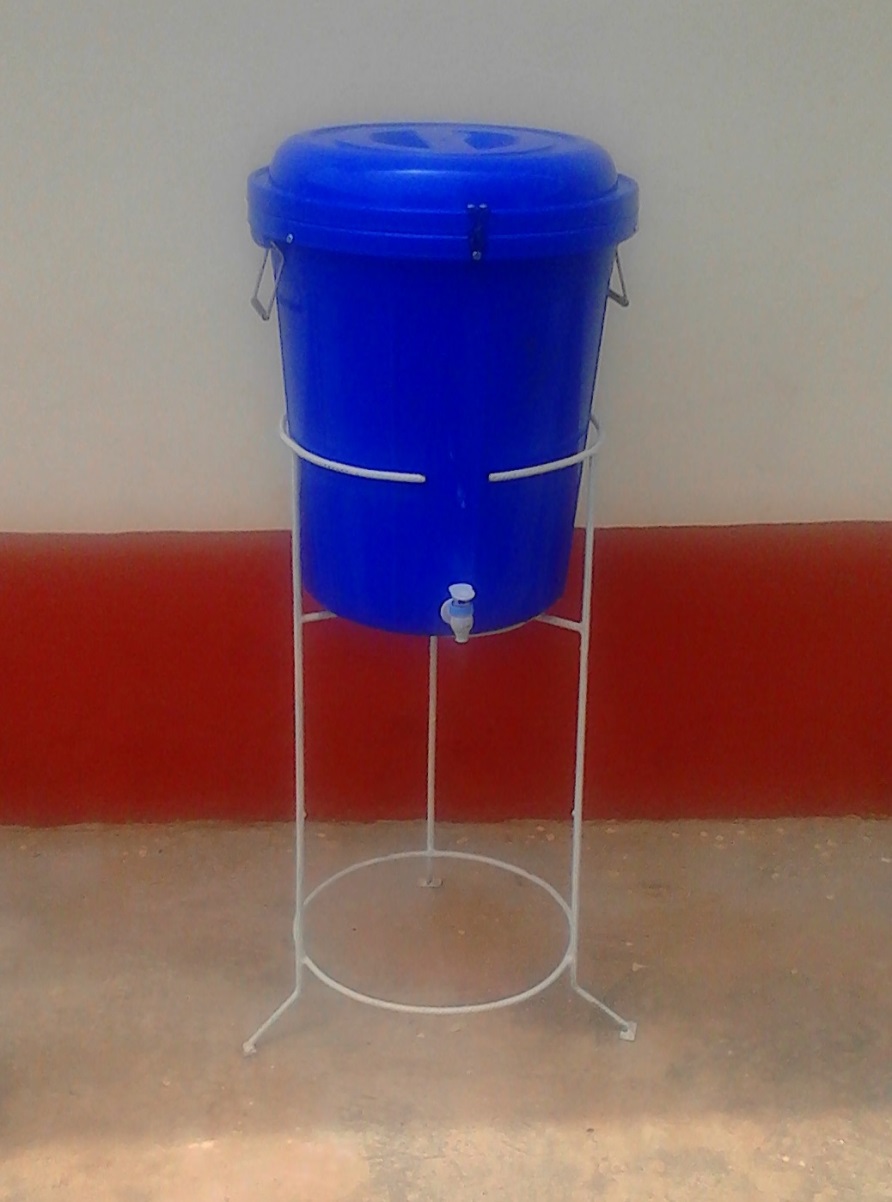**  Plastic safe drinking water storage container | |
| --- | --- |
| **Characteristic** | **Details** |
| Height | Height of stand = 30 inches  Height of stand with drum fitted = 44 inches |
| Diameter | Diameter of drum at the lid = 16.5 inches |
| Volume (liters) | 40 liters |
| Container material | Plastic |
| Tap description & materials | A plastic faucet is attached to the drum.  To open the tap, the faucet head is lifted and to close, it is tuned down.  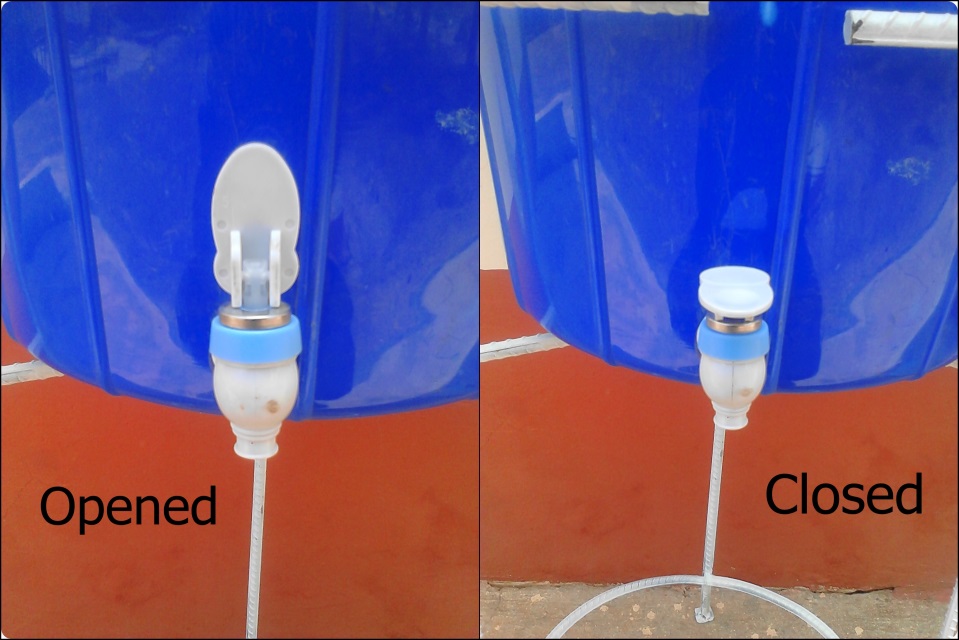 |
| Stand description & materials | Material = Iron, Painted white  Weight = 3.1 kg  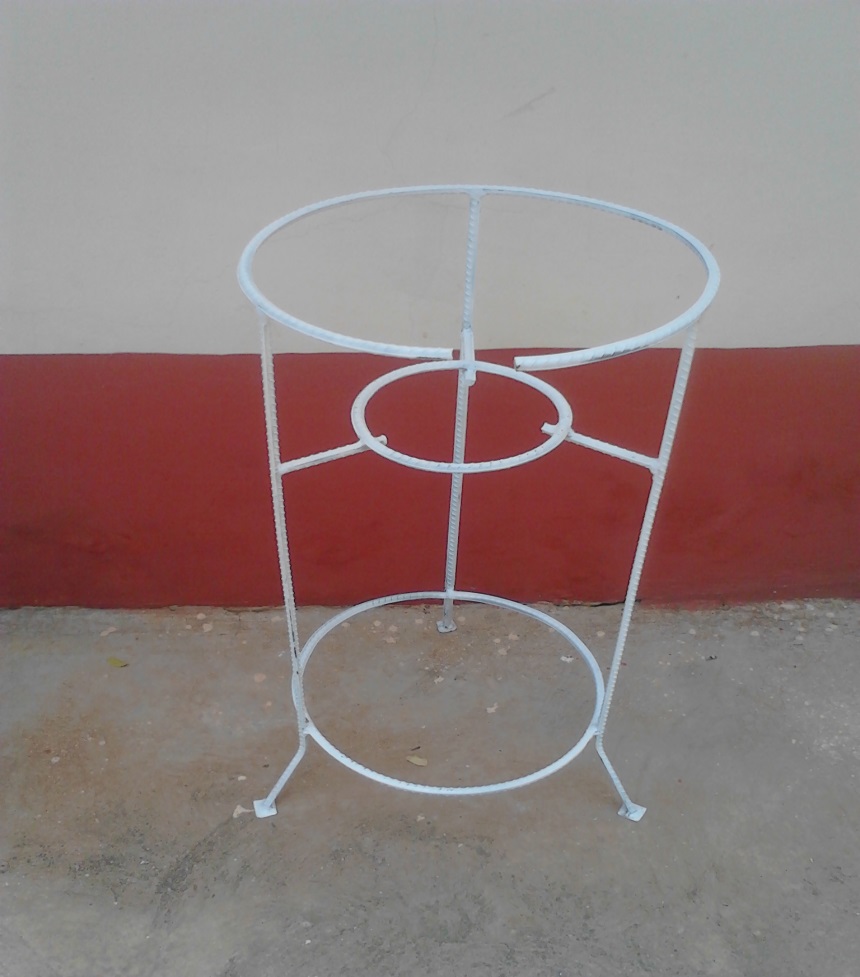 |
| Lid description & materials | Material = Plastic  Two lids are used in each container. A round opening 6.5 inches in diameter is cut in the inner lid, which is then attached to the drum with screws and adhesive. Thin metal bars are fitted to the inside of the drum to cover this opening in a “T” shape, with a gap on the side facing the tap just large enough to allow the tap to be fitted. This screened opening is designed to allow for filling with water, while preventing the dipping in of hands with cups, ladles, and other water collection vessels. The second lid is attached to the first by a hinge and it serves the purpose of keeping the container closed when not in use. A lock is provided to enable the user to lock the drum, if desired. |
| Pictures of the lid | 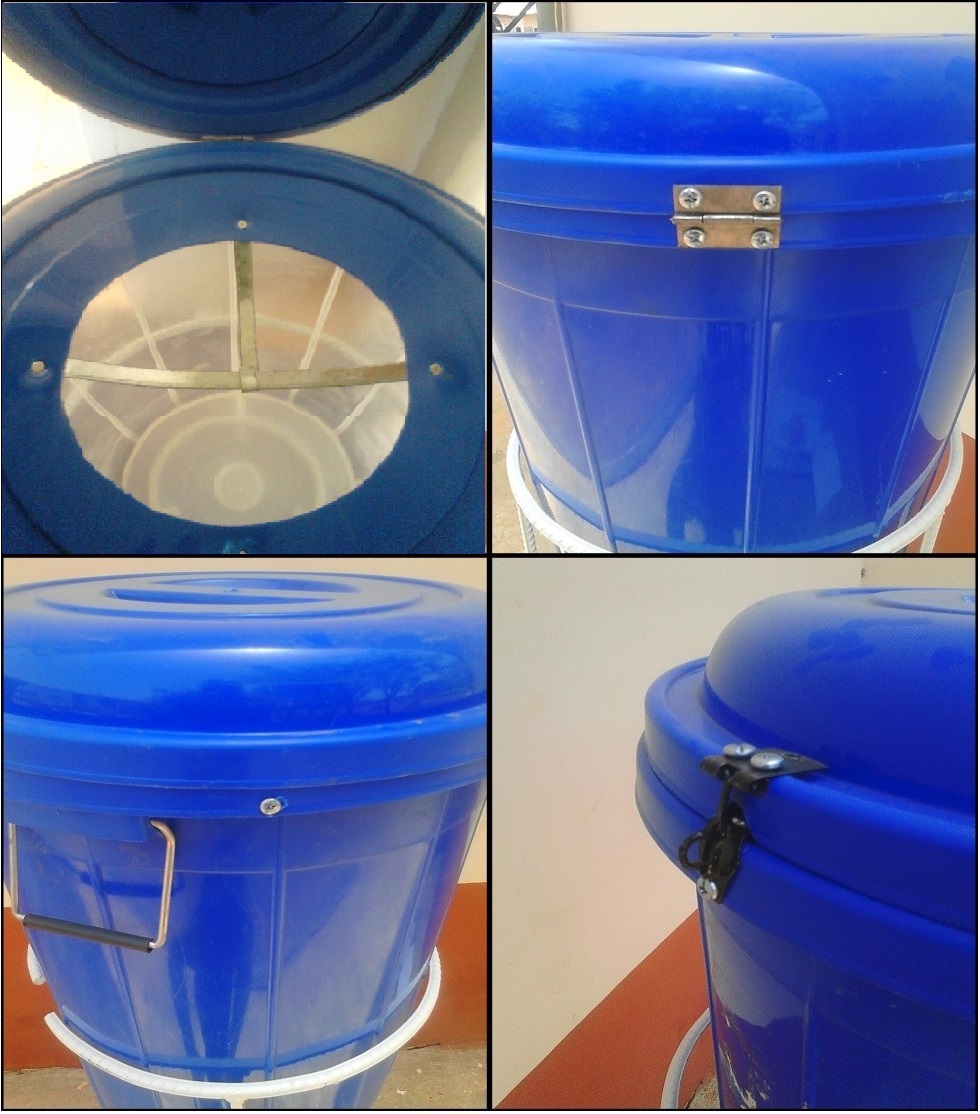 |
| Unit cost | GHC 83.50; $ 21. 91 |

| **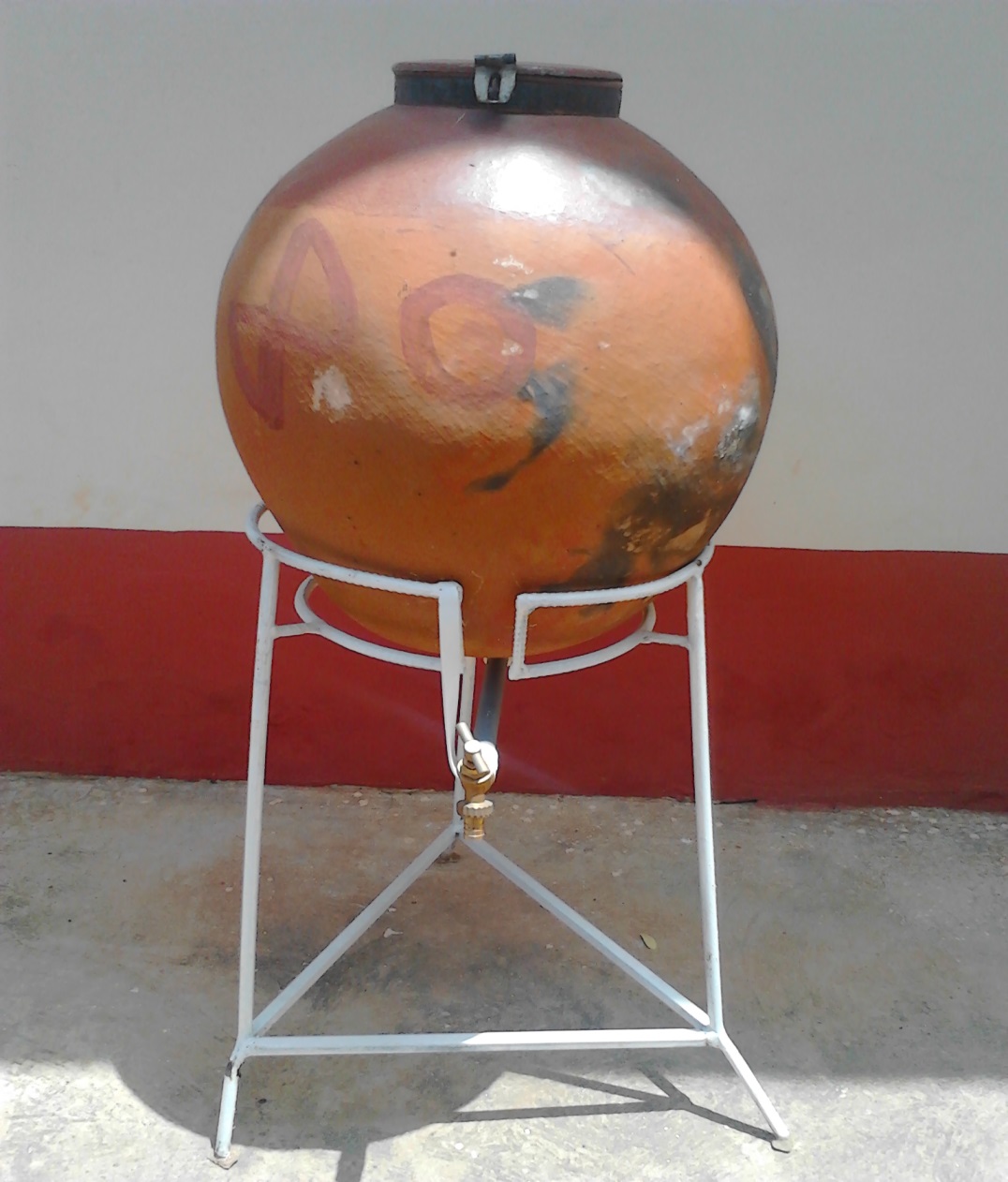**  Ceramic safe drinking water storage container | |
| --- | --- |
| **Characteristic** | **Details** |
| Height | Height of stand = 29.5 inches  Height of stand with drum fitted = 48 inches |
| Diameter | Diameter of drum at its mid-section = 19 inches (approx.) |
| Volume (liters) | 90 liters (Average) |
| Container material | Clay |
| Container weight | 21.3 kg |
| Tap description & materials | A brass faucet is attached to a PVC pipe outlet which is connected to the base of the container.  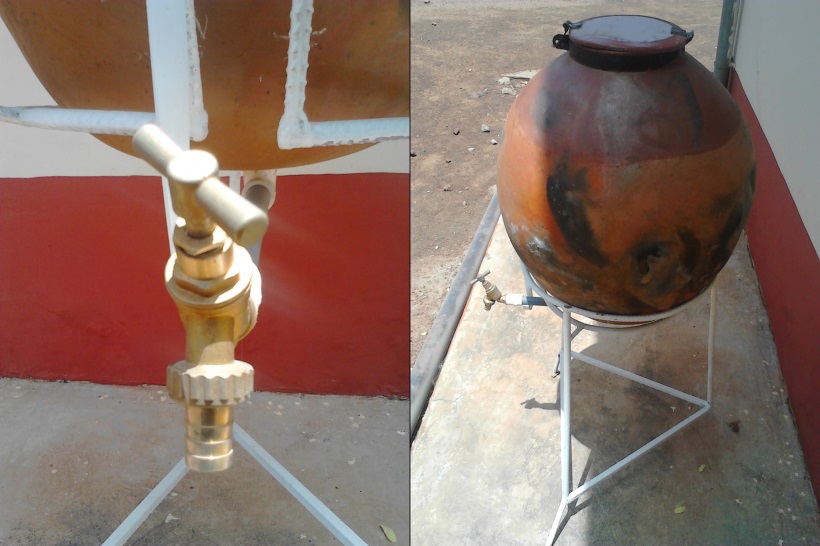  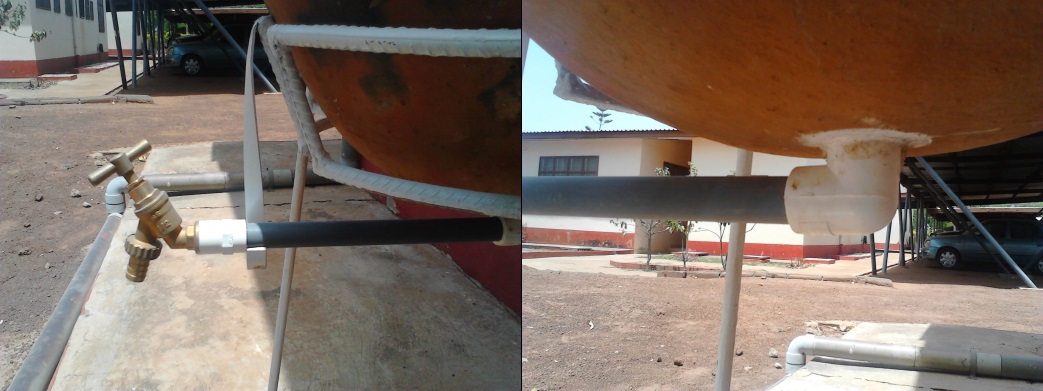 |
| Stand description & materials | Material = Iron, Painted white  Weight = 6.3 kg  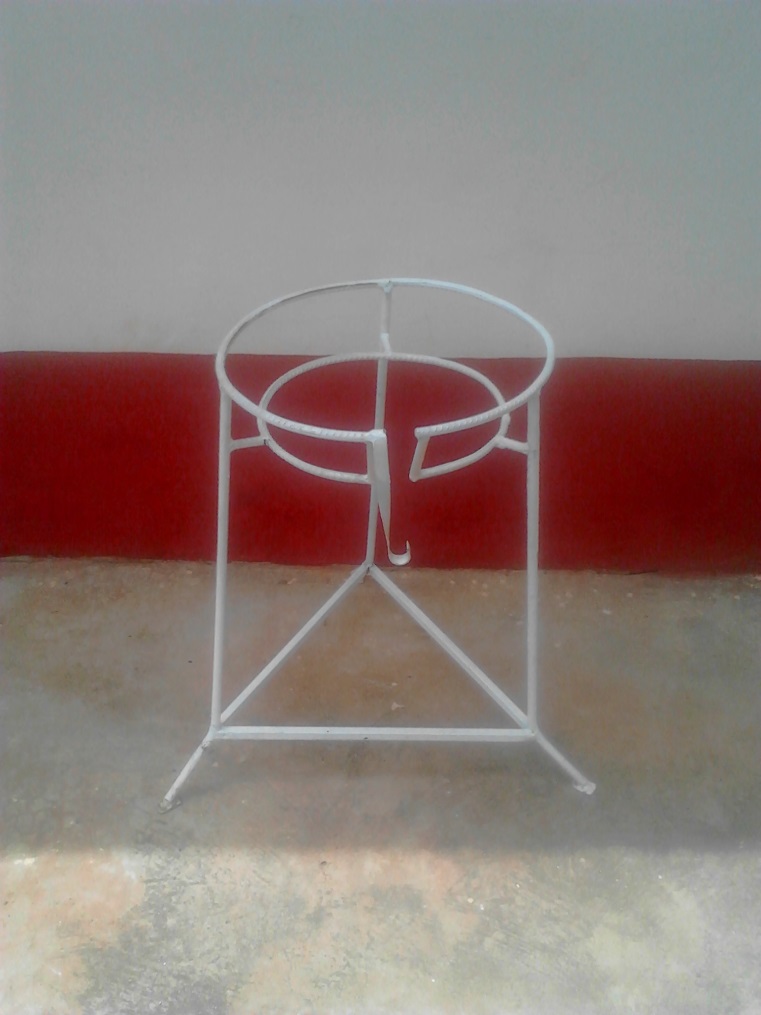 |
| Lid description & materials | Material = Galvalum (55% Al-Zn coated sheet steel)  Lid diameter = 6.57 inches  One metal lid is provided. The lid is secured to the mouth of the ceramic pot by a metal band. A hinge is provided to secure the aluminum plate to this band. The vessel mouth is narrow to prevent dipping. This is to allow for filling with water, yet small enough to prevent the dipping in of hands with cups and other water collection vessels. A lock is provided to enable the user to lock the container. |
| Pictures of the lid | 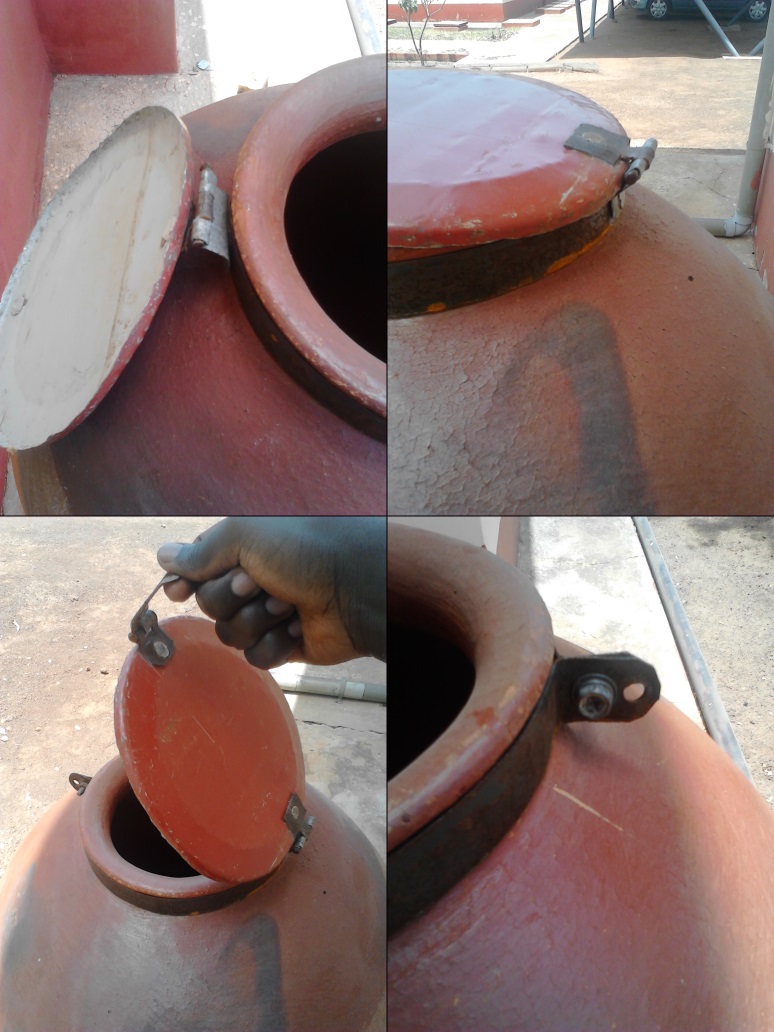 |
| Unit cost | GHC 80.00; USD $20.98 |

All Containers were cleaned and disinfected prior to distribution:

CLEANING PROTOCOL FOR PLASTIC & CERAMIC DRINKING WATER STORAGE CONTAINERS

Materials:

1. Sponge or scrub brush
2. Bucket with water
3. Rubber gloves
4. Dilute chlorine solution
5. Running tap water
6. Mild soap
7. Properly ventilated environment

PROCEDURE:

1. Wash hands with clean water and soap.
2. Dry hands and put on rubber gloves.
3. Clean the vessel that is used for carrying water with a cloth to remove dirt and debris.
4. Place the drinking water storage vessel on a clean surface above the floor.
5. Inspect the drinking water storage vessel to check for any stagnant water.
6. If there is water in the vessel, drain the water through the spigot.
7. Pour water into the water vessel and rinse the lid, interior and exterior walls.
8. Physically scrub or pressure‐wash the lid, interior and exterior walls with mild soap to remove any sediment.
9. Drain wash water.
10. Rinse the lid, interior and exterior walls with clean potable water.
11. Drain wash water.
12. Fill the water storage vessel with clean potable water.
13. Add the required amount of dilute chlorine solution (1:100 solution of bleach [5% sodium hypochlorite] in water)
14. To disinfect the plumbing lines and fixtures, open all taps in the distribution system until chlorine smell is apparent at each outlet. Close taps.
15. Let chlorine solution sit in the water storage vessel for at least 20 minutes.
16. Drain the chlorine solution in the water storage vessel after 20 minutes.
17. Wash lid with chlorine solution.
18. Refill with fresh potable water.
19. Open the pipe valve and allow running water to flow through the interior, the lid and exteriors until there is no smell of chlorine.
20. Empty the container and Allow to dry.
21. Store in a safe, dry, well ventilated place.

How to prepare a 1:100 dilute chlorine solution.

A 1:100 dilute chlorine solution is 5 ml (1 teaspoon) of chlorine solution + 500 ml (2 cups) of water.

DISTRIBUTION PROTOCOL FOR WATER STORAGE VESSELS

This protocol is intended facilitate the distribution of water storage vessels to CQI intervention communities. The distribution will be in two phases in order to afford consistent monitoring and evaluation.

1. Pre-distribution phase.
2. Identify the CQI intervention community, and obtain the GPS coordinates and main respondent names for selected intervention households.
3. Plan the day/date and time of distribution of the vessels. Take into consideration, farming activities, market days and important local festivities. Plan to distribute in the period you are most likely to reach the intervention/selected households.
4. Place in a requisition for water storage vessels; the required number of water storage vessels should be equal to the number of households surveyed in the intervention community, + an extra vessel in case of breakage.
5. The drinking water storage vessels should be appropriately cleaned. (See cleaning protocol)
6. After cleaning and drying, place lids on all the vessels.
7. Carefully place each vessel in a transparent plastic bag to prevent possible dust and introduction of foreign material during transportation.
8. Make the necessary transportation arrangements prior to the day of distribution.
9. The time period between cleaning and distribution should be no more than 1 week.
10. Distribution phase.
11. Inform the WaSH committee (WSMT) chairman or appropriate representative about the intended visit and the selective distribution.
12. Print out the GPS location of the selected households and the corresponding name of the main respondent.
13. Transport the storage vessels to the selected community.
14. Pay a courtesy call on the chief (with or without WSMT representative) explaining the rationale behind the selective distribution.
15. Use a GPS device to locate selected households.
16. Confirm household location by checking the household ID code on the structure.
17. At the household, greet appropriately and provide the vessel to the respondent with detailed instructions (See: Instructions to households).
18. In each selected household, physically use the vessel to demonstrate how to fill, how to dispense water and how to minimize strain on the tap when dispensing water. Allow the recipient to practice fetching water in your presence.
19. When done with the distribution, return to say thank you to the WSMT chairman or representative and the Chief of the community.

INSTRUCTIONS TO HOUSEHOLDS

Instructions to household users of plastic and ceramic water storage containers

1. The household does not have to accept the container, but it is theirs to use for drinking water storage if they want it.

2. The container is being given to those households that were randomly selected for survey questions about water and sanitation earlier.

3. It is designed to keep the water safe so that no contamination occurs, in order to help prevent illness.

4. It only works if water is always fetched using the tap— users should never serve water by dipping in from the top.

5. The container must be kept covered at all times, except when it is being filled

6. The container should be kept filled with water from a safe source, such as a borehole or a piped supply.

7. All members of the household should drink exclusively from the safe storage container when at home; they should refrain from drinking from other containers without lids and taps, as these are more likely to become contaminated.

8. Children who are old enough to drink water should also drink exclusively from this container when at home. If they are not able to fetch from this container themselves, an adult should serve them water from this container when they want to drink.

9. The UNC enumerators will return to ask more survey questions about water, sanitation, and hygiene in the future. The family should answer as honestly as possible so that we can learn if the safe water storage containers are working as intended.

10. Once the study is finished, if the results indicate that the containers are working as they should, it may help World Vision and the District Assembly learn how to better ensure that communities have safe drinking water in the future.

11. Cleaning should be done no more than once per month. You should be sure not to insert bare hands into the container when cleaning— you can fill part way with water, add two capfuls of chlorine if available, and scrub with a clean brush, if they have one. Otherwise, a clean piece of cloth can be used for cleaning.

12. Thank the household for their cooperation.

# WaSH Committee Refresher Training

The interventions include the following:

1. Refresher training of Water and Sanitation Management Teams (WSMTs).
2. Refresher training of Pump Maintenance Volunteers (PMVs).

This report is in two parts. Part one covers the refresher training of WSMTs; part two covers the PMV refresher training

1. Objectives of the WSMT refresher training

The general objective of the refresher training was to increase the capacity of the WSMTs with respect to knowledge, skills and confidence in managing water, sanitation and hygiene related issues in their communities

The specific workshop objective was to discuss the following topics with the WSMTs:

- COLLECTIVE TASKS of the WSMT
- INDIVIDUAL TASKS of committee members (eg. chairperson, secretary)
- ORGANISATION - how to work effectively together as a team
- MEETINGS - how to organize meetings
- TYPE OF FACILITIES - how to select appropriate facilities for the community
- SITING - how to identify good sites for the facilities
- MONEY- how to collect money and how to keep it safe
- CONSTRUCTION - how to assist with the construction
- MAINTENANCE - how to keep the new facility operating; storage of tools
- HYGIENE & SANITATION - how to improve your health
- ACTION PLAN – activities to be carried out
- PROBLEMS & SOLUTIONS - how to resolve problems within the WSMT and the community

At the end of the refresher training, WSMTs were expected to be able to

- Regularly organize WSMT meetings and community meetings
- Plan and help build the new water facilities.
- Regularly check on the use of facilities and distribution of water.
- Organize maintenance and repair of the facilities.
- Collect and manage money to pay for repairs.
- Keep a record of money collected and money spent.
- Organize regular cleaning and maintenance of water points.
- Promote the improvement of hygiene and sanitation in their communities.
- Develop links with other WSMT, DWST and others

1. Methodology

a. The following methods were employed for the WSMT Training

- Brainstorming
- Sharing of experiences
- Group discussions
- Role playing
- Group presentations
- Question and answer

1. Opening ceremony

The WSMT training workshop was opened by Mr. Bansaga Saga, the Project Manager of GI-WASH, Savelugu. He welcomed the participants and encouraged them to take full advantage of the training to upgrade their skills for the development of their communities. He indicated that their training was to build their capacities so that they could adequately function, properly maintain facilities so that the interventions made could serve future generations as well. Other participants also expressed their gratitude for the training and indicated that they were very grateful for the support that World Vision had rendered to their communities over the years especially with regards to water provision in their communities.

Fig. 1


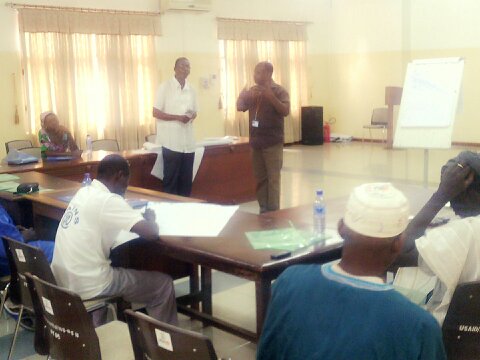


Mr. Bansaga Saga, Project Manager of GI-WASH, gives a welcome message to the participants.

1. Summary of presentations.

The following is a highlight of the major themes and sub issues that were discussed

Fig. 2


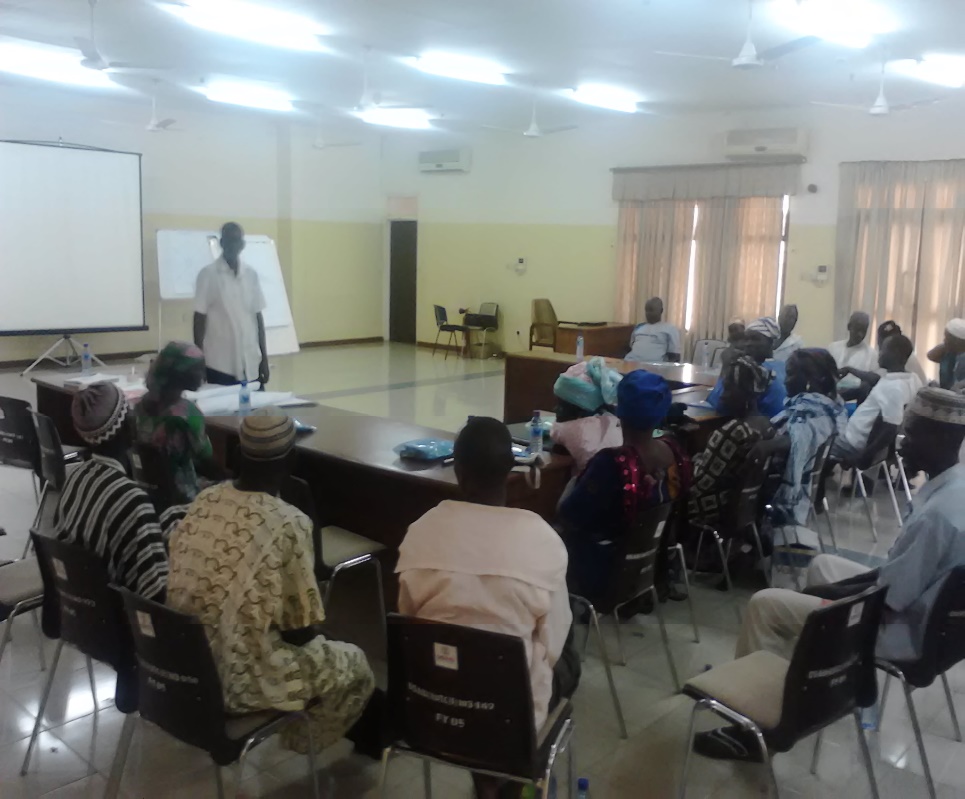


1. Collective tasks

- Organize WSMT meetings and meetings with the entire community.
- Plan and help build the new water facilities.
- Check on use of facilities and distribution of water.
- Organize maintenance and repair of the facilities.
- Collect and manage money to pay for repairs.
- Keep a record of money collected and money spent.
- Organize regular cleaning and maintenance of pump site.
- Promote the improvement of hygiene and sanitation.
- Develop links with other WSMTs, DWST and others.

1. Individual tasks

- What TASKS have been assigned to each member?
- What are your expectations of each person/position?

1. Organization

- How are you working as a team? Any problems or conflicts?
- How are relations with community leaders? Any problems?
- What problems have the community had with committees in the past?
- How will you organize yourselves to avoid/overcome these problems?
- How will you ensure that:

a) Women are actively involved - but not overloaded with work?

b) Every member is involved in discussion and decision-making?

c) Work gets done and is shared among the members?

D. Meetings

- How are you keeping the community informed and involved?
- How are your own meetings and the community meetings - attendance? participation? concrete results?
- How do you organize your meetings - your own meetings and meetings with the community - so that they are effective & efficient?

E. Type of facilities

- What technical option(s) did the community choose? Why? If the water table is low - only boreholes may be possible, most familiar technology; cost concerns; pressure by leaders.
- Which site(s) did the community choose? Why?
- How did you involve the community in these decisions? Did you have any problems in getting their input?
- Does the community really understand why they are choosing a particular option? If not, what can you do to discuss it further?

F. Siting

- “What things do you need to consider in siting the new facilities?” Walking distance, distribution of facilities, proximity to latrines/refuse dumps/cemeteries/cattle kraals, location of groundwater, etc.
- “Where would you like to site the new water supply?” Get them to suggest a number of possible sites. Use the COMMUNITY MAP to mark possible sites. Discuss the hygiene implications of different sites.
- “How would you organize a community meeting and site visits to get the community to agree on the siting?”

G. Money

- How much money have you collected for maintenance?
- How are you organizing the collection of money - how often? how much per person? who collects? how?
- How are you recording contributions?
- What problems do you have with collecting money?
- How can you solve those problems?
- What will you do if people refuse to contribute?
- Where do you keep the money? (home or bank)
- If at bank, who authorizes the withdrawal of money to buy things
- Any problems with keeping money safely? Solutions?
- How will you organize the purchase of spare parts?
- Where can they be purchased? How much?
- Which parts of the pump need regular replacement and what is the annual cost for spare parts.
- Strategy for regular purchase of spare parts.

Investing Money

- What are you going to do with the money -

a) buy and store spare parts?

b) leave the money in the bank to accumulate interest?

c) buy groundnuts during harvest and resell later?

Record-Keeping and Accountability

- Who is keeping the records? How are you doing it?
- Who is checking the record-keeping?
- How do you report on money to: a) WSMT? b) Community?

H. Maintenance

- Pump users using pump with care
- Caretakers inspecting pump regularly
- Stock of spare parts on hand
- Quick action when pump breaks down
- What are the pump parts that spoil quickly? How will you get spare parts?
- What will you do when the pump spoils?
- Regular cleaning and maintenance of pump site
- Maintenance schedule
- Replacement of worn parts, help from Area Mechanics, etc
- Who will you appoint as caretakers? What will be their roles?

I. Hygiene & sanitation

- What are the main health problems in using the old sources? Unsafe water - source of diarrhoea/guinea worm; people defecate near source; chemicals and other things polluting the water; water point is full of weeds and dirt, long distance to get water - women are very tired, etc.

b) How do your water sources get polluted?

c) What are the causes of diarrhoea? guinea worm?

d) What are the health advantages of the facilities? Safe water all year round - less diarrhoea/no guinea worm, use water for hand washing and keeping things clean, etc.

e) What is hand washing? Demonstrate clean hand washing. Who should wash, when to wash and why is hand washing important.

- Stop people from defecating near sources. Stop other forms of contamination.
- Filter or add alum to water before drinking. Clean and weed the site so it is easier for women to fetch water. Widen footpaths to water point.
- Why is ‘free-range’ (open defecation) a health problem?
- Why latrines? What are the benefits of latrines?
- What fears or concerns do you have about latrines?
- What are the problems with communal latrines?

J. Action plan

WSMT Group Discussion

WSMTs agreed on what is to be done for each activity - WHAT is to be done, WHO is

going to do it, and WHEN it will be done and it was recorded on their Action Plan.

- Selection and siting of water facilities
- Completing the Facility Management Plan
- Raising commitment funds for new water supply
- Opening a bank account
- Community discussion and action on hygiene and sanitation

PMV TRAINING

1.0 Objectives of the PMV refresher training

The general objective of the PMV refresher training was to strengthen the capacity of the PMVs to carry out repairs and routine maintenance on their water points with emphasis on India Mark II and AFRIDEV borehole handpumps.

The specific objectives of the refresher training were to discuss the following with the PMVs:

- TOOLS
- TYPES OF BOREHOLES
- HOW BOREHOLES WORK
- CONSTRUCTION REQUIREMENTS
- MAINTENANCE OF BOREHOLES
- TROUBLE SHOOTING
- PRACTICAL FIELD MAINTENANCE AND REPAIR

At the end of the training, PMVs were expected to be able to:

- Identify the tools used to repair and maintain hand pumps.
- Identify the parts of the India Mark II and AFRIDEV handpumps
- Adequately explain how the above-ground and below-ground components of the handpumps work
- Explain requirements for borehole construction
- Develop and implement a routine maintenance schedule
- Individually identify and propose solutions to problems with the usage of hand pumps
- Have hands-on experience with repair, dismantling, re-assembly and maintenance of India Mark II and AFRIDEV hand-pumps.

2.0. Methodology

The following methods were employed for the PMV training.

- Video presentations
- Brainstorming
- Sharing of experiences
- Group practice sessions
- Individual practice sessions
- Trouble-shooting, questions and answers

Fig. 3


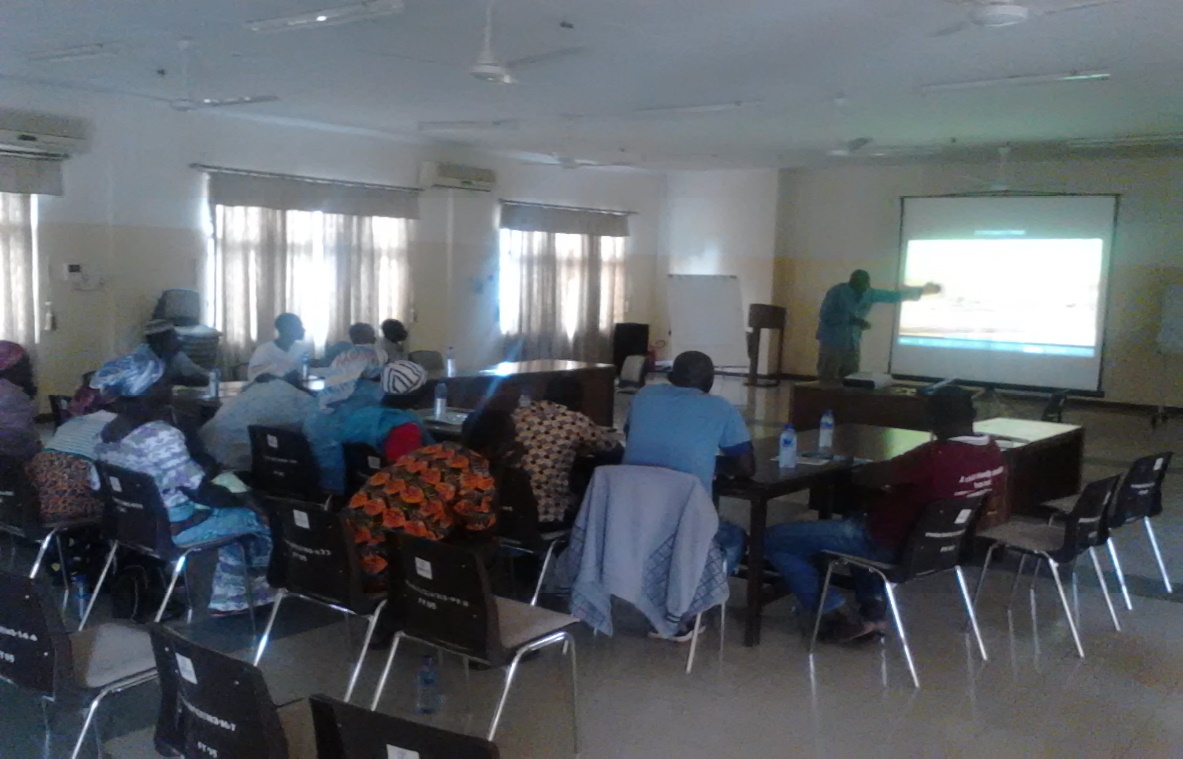


Mr. Adam Ussif, Facilitator of the PMV training, using a video presentation to explain how parts of the India Mark II handpump work.

3.0 Summary of presentations

- TOOLS

The PMVs were trained on use of the following tools.

| Pipe/rising mains/ lifting spanner | Hack saw |
| --- | --- |
| Pipe vice, self-locking clamp | Rod lifter |
| Fixed/open ended spanner 17 x 19.  (17 x 19 combination flat spanner) | Pipe wrenches (600mm) and (450mm) |
| Crank (axel rod) spanner 17 x 19 | Handle axle punch. |
| Connecting rod vice | Steel brush |
| Pipe Die | Chain supporting tool |
| Connecting Rod Die | Coupling spanner |
| Bearing pressing tool | Pipe lifter |
| Rod clamp | Ring spanner |
| Chain fork |  |

- TYPES OF BOREHOLES

Participants were introduced to the parts of the India Mark II and the AFRIDEV handpumps. The facilitator explained how the component parts work to draw water from the ground to the surface.

Parts of the India Mark II

1. The handle assembly
2. The pedestal
3. The water tank assembly
4. The pump head assembly
5. Connecting rods and rising mains
6. Pump cylinder

Parts of the AFRIDEV hand pump

1. Handle
2. Pump head
3. Spout
4. Pumpstand
5. Cement plat form
6. Socket
7. Pumprod
8. Plunder
9. Cylinder
10. Footvalve
11. Centraliser

- CONSTRUCTION REQUIREMENTS

The participants and the facilitator together discussed general construction requirements for the installation of boreholes. Participants understood that

a) The well should be in an elevated place, so that during the rainy season

the water will run away from it, rather than into it.

b) It should be at least 30 meters away from a latrine and uphill of the latrine.

c) It should be at least 30 meters away from a cattle enclosure

d) It should be well away from any depressed area in the ground, such as hollows that are used for rubbish tipping, hollows that are used for brick making or any other areas where water might collect.

- TROUBLE SHOOTING

Participants together with the facilitator discussed what to do when the borehole:

1. Draws rusty water
2. Pumps out water containing worms
3. Pump has no resistance and no water comes out when strokes are given
4. Pump weight is usual but no water comes out
5. Pump is very light but no water comes completely
6. Water takes very long to come even after a brief stopover.
7. Water only comes well during rapid pumping
8. Unusual noise when pumping
9. Muddy or silty water pumps out

- PRACTICAL FIELD MAINTENANCE AND REPAIR

Fig. 4


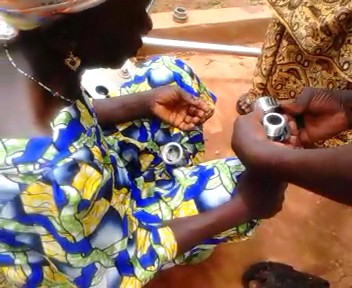


A woman from Zosali being taught how to assemble the India Mark II pump plunger.

Fig. 5


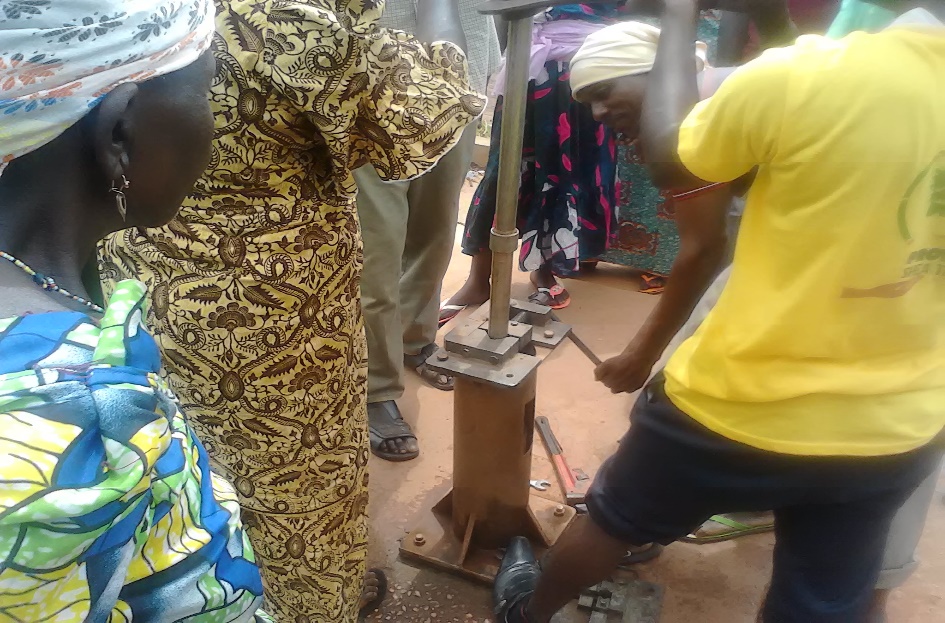


A man from Tigu taking his turn at using the pipe clamp to secure the pipe.

Fig. 6


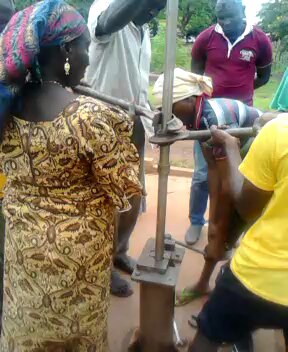


Participants take their turn in demonstrating how to use the pipe lifter to lift the pipe out of the metal casing.

Fig. 7


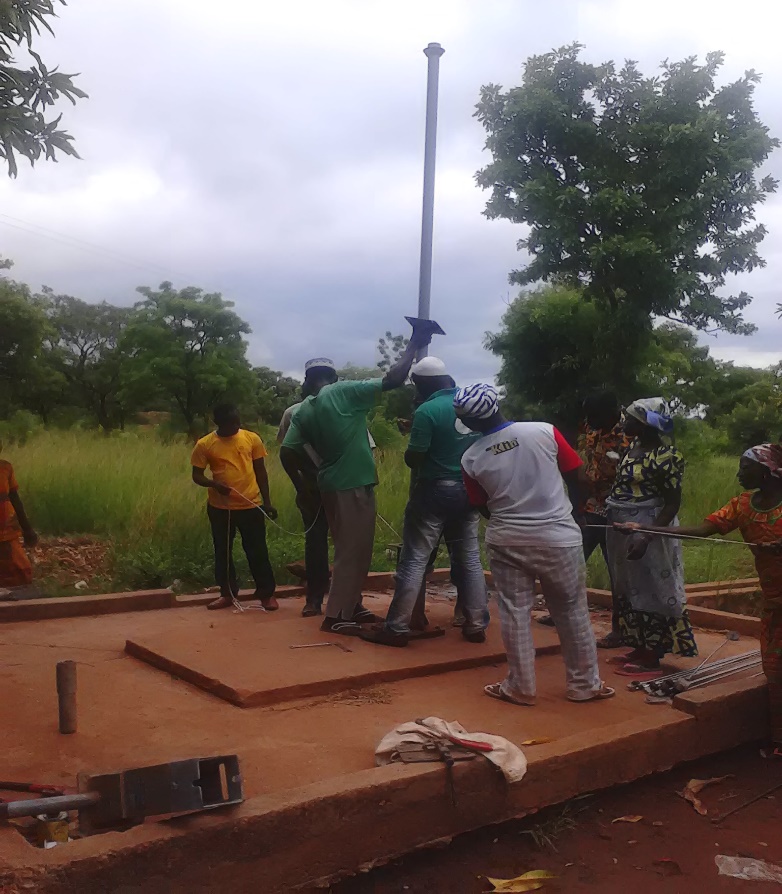


Participant help in the re-assembly of the AFRIDEV hand pump

- 1. Closing the refresher training

Mr. Dominic Dapaah, the WASH coordinator at World Vision GI-WASH, Savelugu closed the PMV training session on behalf of Mr. Bansaga Saga, the Project Manager of GI-WASH. He indicated that the training was part of a series of pilot interventions aimed at improving water, sanitation and hygiene services in the district. He encouraged the participants to apply what they learnt at the training to benefit the communities from which they came. Also he stressed the importance of application, saying other communities in the district will be observing them and that their performance will encourage an up-scaling of the interventions to communities in the district. He found out what participants had learnt at the end of their training.

Fig 8


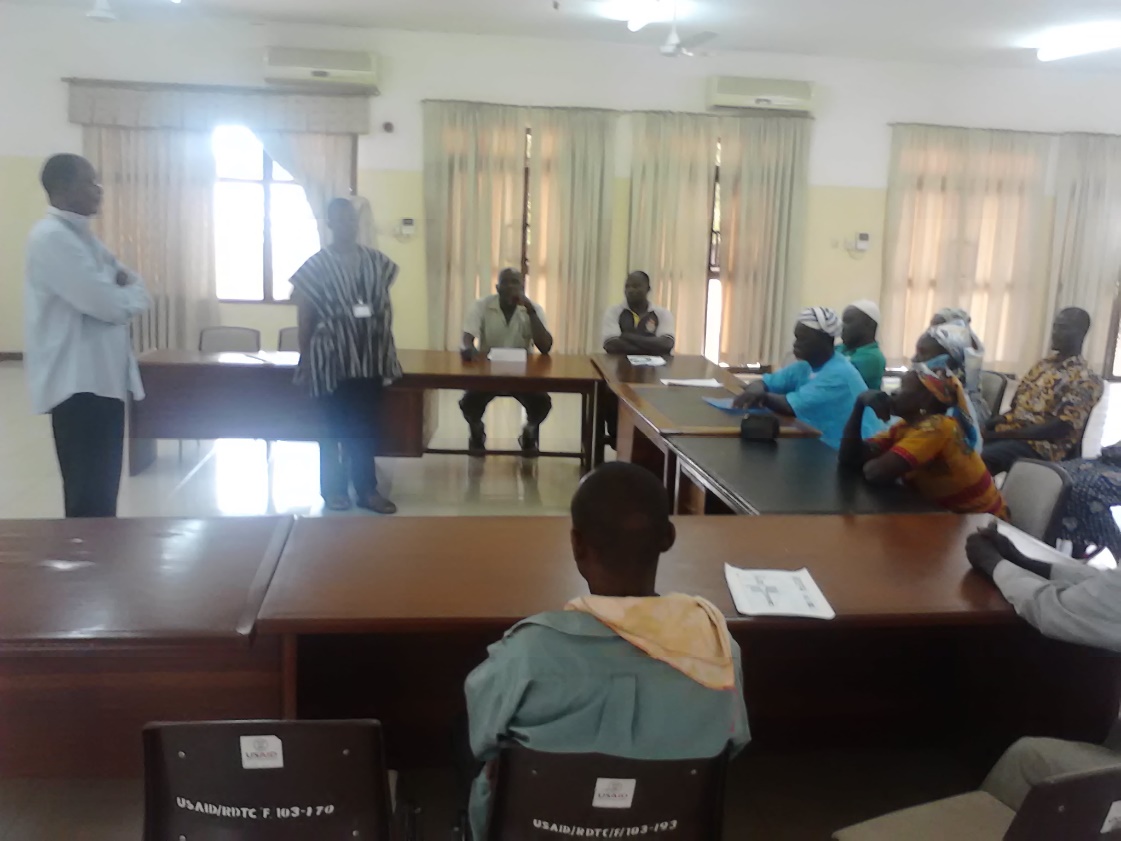


Mr. Dominic Dapaah, WaSH Coordinator, interacts with the PMVs at the end of their training session.

Appendix

EXAMPLE WSMT REFRESHER TRAINING PLAN

| **TARGET GROUP** | **DAY** | **START TIME** | **END TIME** | **EVENT** |
| --- | --- | --- | --- | --- |
| WSMTs | Day 1 | 8:00 am | 9:00 am | GATHERING AND WARMUP |
|  |  | 9:00 am | 9:30 am | GETTING STARTED |
|  |  | 9:30 am | 10:00 am | TASKS, ORGANISATION, AND RELATIONS |
|  |  | 10:00 am | 10:30 am | BREAK |
|  |  | 10:30 am | 11: 00 am | WATER SUPPLY OPTIONS AND SITING |
|  |  | 11: 00 | 1:00 pm | CONSTRUCTION & MAINTENANCE |
|  |  | 1:00 pm | 2:15 pm | LUNCH |
|  |  | 2:15 pm | 3: 30 pm | HYGIENE AND SANITATION |
|  |  | 3: 30 pm | 3:45 pm | ACTION PLANNING |
|  |  | 3:45 pm | 4: 00 pm | EVALUATION |
|  |  | 4:00 pm | | CLOSING |
|  | Day 2 | 8:00 am | 9:00 am | GATHERING AND WARMUP |
|  |  | 9:00 am | 9:30 am | LEARNING REVIEW |
|  |  | 9:30 am | 10:00 am | PROBLEM SOLVING |
|  |  | 10:00 am | 10:30 am | BREAK |
|  |  | 10:30 am | 11: 30 am | MEETINGS SKILLS |
|  |  | 11:30 am | 12:30 pm | RECORD-KEEPING |
|  |  | 12: 30 pm | 1:00 pm | COLLECTION AND MANAGEMENT OF MONEY |
|  |  | 1:00 pm | 2:15 pm | LUNCH |
|  |  | 2:15 pm | 3: 00 pm | HYGIENE EDUCATION  (Role playing and discussion) |
|  |  | 3: 00 pm | 3:30 pm | SANITATION  (large group - picture- discussion) |
|  |  | 3:30 pm | 3:45 pm | ACTION PLANNING |
|  |  | 3:45 pm | 4:00 pm | EVALUATION |
|  |  | 4:00 pm | | CLOSING & PICTURES |

**PMV REFRESHER TRAINING PLAN**

| **TARGET GROUP** | **DAY** | **START TIME** | | **END TIME** | **EVENT** |
| --- | --- | --- | --- | --- | --- |
| PMVs | Day 1 | 8:00 am | | 10:00 am | Introduction to Pump Maintenance Tools |
|  |  | 10:00 am | | 10:30 am | BREAK |
|  |  | 10:30 am | | 12:00 pm | How boreholes work |
|  |  | 12:00 pm | | 1:00 pm | Construction requirements |
|  |  | 1:00 pm | | 2:15 pm | LUNCH |
|  |  | 2:15 pm | | 3:30 pm | Maintenance – India Mark II |
|  |  | 3:30 pm | | 4:00 pm | Maintenance - AFRIDEV |
|  |  | 4:00 pm | | | CLOSING |
|  | Day 2  Field Practice – India Mark II | 8:00 am | | 10:00 am | Dismantling |
|  |  | 10:00 am | | 10:30 am | BREAK |
|  |  | 10:30 am | | 1:00 pm | Individual practice on component parts |
|  |  | 1:00 pm | | 2:15 pm | LUNCH |
|  |  | 2:15 pm | | 3:15 pm | Trouble shooting |
|  |  | 3:15 pm | | 4:00 pm | Reassembly |
|  |  | 4:00 pm | | | CLOSING |
|  | Day 3  Field Practice – AFRIDEV | 8:00 am | 10:00 am | | Dismantling |
|  |  | 10:00 am | 10:30 am | | BREAK |
|  |  | 10:30 am | 1:00 pm | | Individual practice on component parts |
|  |  | 1:00 pm | 2:15 pm | | LUNCH |
|  |  | 2:15 pm | 3:30 pm | | Trouble shooting |
|  |  | 3:30 pm | 4:00 pm | | Reassembly |
|  |  | 4:00 pm | | | CLOSING |

Sources of materials used for WSMT refresher training

- WATSAN Handbook. Community Water and Sanitation Agency, Ghana
- Trainers Guide for WATSAN training. Community Water and Sanitation Agency, Ghana

Sources of materials used for PMV refresher training

- Installation and Maintenance manual for the Afridev Handpump. Skat_Foundation. www. skat.ch.
- India Mark II installation and maintenance manual for hand pump technicians and borehole caretakers. Action Against Hunger (ACF) International. ACF-WASH, 2010.

# 3. Assessment of WaSH committee toolkits and replacement of missing tools

Distribution of tools to WSMTs was conducted in two phases

1. Assessment of existing tools and their condition during community uptake surveys
2. Distribution of missing tools to WSMTs

Following the distribution of safe water storage containers and the refresher training of WSMTs, uptake surveys were conducted to confirm that communities had received these interventions, and determine whether uptake of the improvement package was occurring (Appendix 9). As part of these uptake surveys, enumerators assessed the tools possessed by each WSMT for repair of water sources in the community, and the condition of these tools. Briefly, available tools were compared to a list of required tools for the maintenance of different types of water sources. In each community, the required tools were determined based on the types of water sources present in the community. For each community, any missing and/or broken tools were documented.

Tools included in WSMT tool kits, by system type

| Tool | System type | Pump Type |
| --- | --- | --- |
| Pipe wrench | All |  |
| Grip pliers (Vice grips) | All |  |
| Crank spanner | All |  |
| 17*19 Combination flat spanner | Borehole with Handpump | India Mark II |
| 19 Combination spanner | Borehole with Handpump | India Mark II |
| Rod lifter | Borehole with Handpump | India Mark II |
| Rod clamp | Borehole with Handpump | India Mark II |
| Pipe lifter | Borehole with Handpump | India Mark II |
| Pipe clamp | Borehole with Handpump | India Mark II |
| 22-24 Ring spanner | Borehole with Handpump | India Mark II |
| Axle punch | Borehole with Handpump | India Mark II |
| Chain fork | Borehole with Handpump | India Mark II |
| Chain support | Borehole with Handpump | India Mark II |
| Bearing Presser | Borehole with Handpump | India Mark II |
| Afridev Socket Spanner | Borehole with Handpump | Afridev |
| Afridev Fishing Tool | Borehole with Handpump | Afridev |
| Nira Allen key 10mm | Borehole with Handpump | Nira |
| Nira F-key. | Borehole with Handpump | Nira |

Following documentation of missing tools, replacement tools were procured from local and international suppliers. The quantity of tools procured was equal to the documented tools in need of replacement, plus a 10% surplus to address unforeseen contingencies. Procurement of the tools proved very time consuming, as some items were shipped from suppliers in India, and encountered extensive delays during shipping and customs clearance. Once tools arrived, they were distributed to all communities that had been documented as requiring additional tools. Distribution of tools was completed in September of 2016.
