# Supplementary material for: WaSH CQI: Applying continuous quality improvement methods to water service delivery in four districts of rural northern Ghana
Source: PLoS One. 2020 Jul 15;15(7):e0233679. doi: 10.1371/journal.pone.0233679 (PMC7363065; doi:10.1371/journal.pone.0233679)
Supplement: Attachment — Submitted filename: Response to Reviewers 5.7.2020 Fisher.docx [file pone.0233679.s011.docx]

Response to Reviewers Updated 5.7.2020

Dear Editor,

Editor’s and reviewers’ additional comments have been addressed and the manuscript and supporting information have been updated accordingly. Responses to each comment are listed below and underlined. These comments have improved the quality and readability of this manuscript.

If there are any questions about these changes or the responses below, please do not hesitate to contact us.

Best regards,

Michael Fisher.

*************************************

PONE-D-19-15425R1

WaSH CQI: Applying Continuous Quality Improvement methods to Water Service Delivery in four districts of rural northern Ghana

PLOS ONE

Additional Editor Comments (if provided):

Some numbers in main text should be corrected following the changes of tables.

L280 (in the file with marked changes)

84%=>86%

L282

52%=>57%

L347

26%=>30%

L348

42%=>43%

These changes have been made as suggested

Please carefully check all the numbers again.

OK

[Note: HTML markup is below. Please do not edit.]

Reviewers' comments:

Reviewer's Responses to Questions

Comments to the Author

1. If the authors have adequately addressed your comments raised in a previous round of review and you feel that this manuscript is now acceptable for publication, you may indicate that here to bypass the “Comments to the Author” section, enter your conflict of interest statement in the “Confidential to Editor” section, and submit your "Accept" recommendation.

Reviewer #1: All comments have been addressed

Reviewer #2: All comments have been addressed

________________________________________

2. Is the manuscript technically sound, and do the data support the conclusions?

The manuscript must describe a technically sound piece of scientific research with data that supports the conclusions. Experiments must have been conducted rigorously, with appropriate controls, replication, and sample sizes. The conclusions must be drawn appropriately based on the data presented.

Reviewer #1: Yes

Reviewer #2: (No Response)

________________________________________

3. Has the statistical analysis been performed appropriately and rigorously?

Reviewer #1: Yes

Reviewer #2: (No Response)

________________________________________

4. Have the authors made all data underlying the findings in their manuscript fully available?

The PLOS Data policy requires authors to make all data underlying the findings described in their manuscript fully available without restriction, with rare exception (please refer to the Data Availability Statement in the manuscript PDF file). The data should be provided as part of the manuscript or its supporting information, or deposited to a public repository. For example, in addition to summary statistics, the data points behind means, medians and variance measures should be available. If there are restrictions on publicly sharing data—e.g. participant privacy or use of data from a third party—those must be specified.

Reviewer #1: Yes

Reviewer #2: (No Response)

________________________________________

5. Is the manuscript presented in an intelligible fashion and written in standard English?

PLOS ONE does not copyedit accepted manuscripts, so the language in submitted articles must be clear, correct, and unambiguous. Any typographical or grammatical errors should be corrected at revision, so please note any specific errors here.

Reviewer #1: Yes

Reviewer #2: (No Response)

________________________________________

6. Review Comments to the Author

Please use the space provided to explain your answers to the questions above. You may also include additional comments for the author, including concerns about dual publication, research ethics, or publication ethics. (Please upload your review as an attachment if it exceeds 20,000 characters)

Reviewer #1: I confirmed all the revisions and agreed them.

It is better to use italic character, when showing statistics　(ex. p-value).

These changes have been made: p-values have been italicized.

Reviewer #2: The new versión is much more clear. Most of the comments has been properly considered.

One minor comment. I propose to reduce the number of decimals in Table 2 from 6 to 3. They are estimations and confidence intervals, not precis mechanical measurements. The table footnote with two stars could be removed (in this table).

This change has been made. Significant figures have been reduced as suggested.

________________________________________

7. PLOS authors have the option to publish the peer review history of their article (what does this mean?). If published, this will include your full peer review and any attached files.

If you choose “no”, your identity will remain anonymous but your review may still be made public.

Do you want your identity to be public for this peer review? For information about this choice, including consent withdrawal, please see our Privacy Policy.

Reviewer #1: No

Reviewer #2: No
